# Supplementary material for: Illness severity and risk of mental morbidities among patients recovering from COVID-19: a cross-sectional study in the Icelandic population
Source: BMJ Open. 2021 Jul 23;11(7):e049967. doi: 10.1136/bmjopen-2021-049967 (PMC8313306; doi:10.1136/bmjopen-2021-049967)
Supplement: Supplementary data [file bmjopen-2021-049967supp001.pdf]

Supplementary material

Supplementary Figure 1. The analytic sample within the COVID-19 National Resilience Cohort.

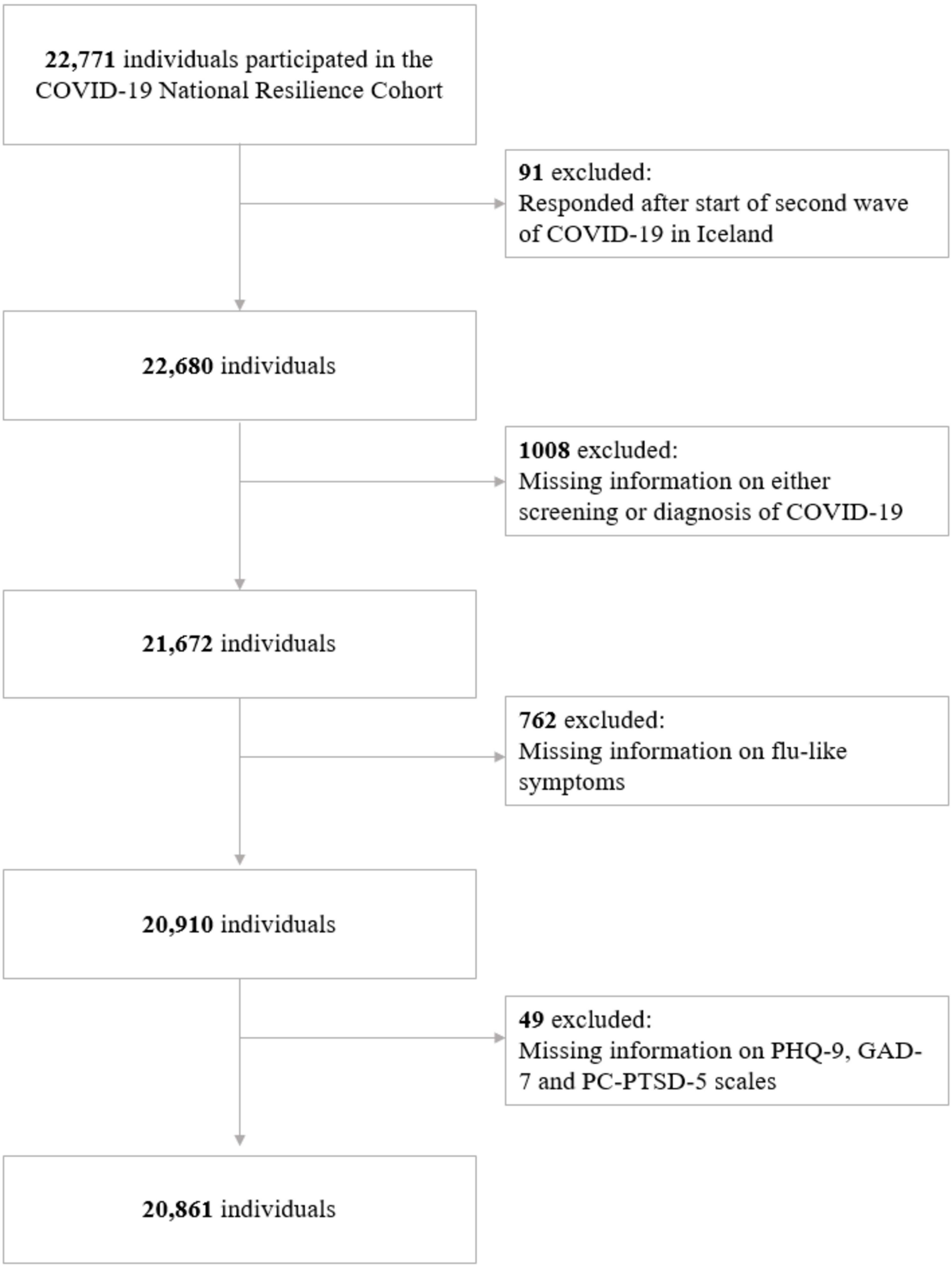

Supplementary Table 1. Mean and median scores on PHQ-9, GAD-7, and PC-PTSD-5 among individuals with and without a recent COVID-19 diagnosis.

	Mean scores		Median scores		P-value	Adj. P-value
	Individuals not diagnosed with COVID-19	Individuals diagnosed with COVID-19	Individuals not diagnosed with COVID-19	Individuals diagnosed with COVID-19		
**Depression**	5.07	6.11	4	5	<.001	<.001
**Anxiety**	4.12	4.52	3	4	.07	.08
**PTSD**	1.72	1.90	2	2	.007	<.001

[a] Adjusted for age (continuous variable), gender (male, female, or other), educational level (compulsory education, high school/trade school/other education, bachelor's degree/diploma certificate, or master's/Ph.D. degree), income (continuous variable), current smoking (yes or no), previous diagnosis of a psychiatric disorder (yes or no), number of previously diagnosed somatic diseases (0, 1, or ≥ 2), and timing of responding to the baseline questionnaire (April 24-30, May 1-7, May 8-23, or May 24-July 22).

Supplementary Table 2. The prevalence, crude and multivariable adjusted relative risks and absolute differences in symptoms of depression (PHQ-9), anxiety (GAD-7) and PTSD (PC-PTSD-5) surpassing screening thresholds among individuals with and without a confirmed diagnosis of COVID-19. The data are presented with stratification of all covariates used in the multivariable adjusted models along with the p-values for the effect modification.

	No. (%)						
	Individuals not diagnosed with COVID-19	Individuals diagnosed with COVID-19	cRR (95% CI)	aRR (95% CI)[a]	Absolute difference Crude %	Absolute difference Adjusted [a] %	P-value for effect modification
**Depression**	2992 (16.2)	75 (22.1)	1.36 (1.11 to 1.67)	1.48 (1.20 to 1.82)	5.9	8.4	
**Female**	2421 (18.8)	56 (24.8)	1.32 (1.05 to 1.66)	1.43 (1.13 to 1.81)	6.0	8.1	0.110
**Male**	559 (10.0)	17 (15.2)	1.52 (0.97 to 2.37)	1.54 (0.96 to 2.47)	5.2	7.2	--
**18-39 years**	954 (33.3)	23 (27.1)	0.81 (0.57 to 1.16)	0.94 (0.65 to 1.36)	-6.2	-1.8	0.025
**40-59 years**	1367 (16.8)	40 (22.3)	1.33 (1.01 to 1.75)	1.72 (1.31 to 2.27)	5.5	14.7	--
**≥60 years**	671 (8.9)	12 (15.8)	1.77 (1.05 to 2.98)	2.57 (1.52 to 4.36)	6.9	21.8	--
**Compulsory education**	540 (21.5)	6 (23.1)	1.07 (0.53-2.17)	1.24 (0.61-2.53)	1.6	4.7	<.001
**Upper secondary, vocational or other education**	996 (17.7)	15 (14.7)	0.83 (0.52-1.33)	0.83 (0.51-1.33)	-2.9	-2.5	--
**Bachelor's degree or diploma certificate**	935 (15.7)	30 (24.2)	1.54 (1.12-2.12)	1.56 (1.16-2.08)	8.5	10.2	--
**Master's or Ph.D. degree**	521 (11.9)	24 (27.3)	2.28 (1.61-3.24)	2.95 (2.03-4.29)	15.3	34.5	--
**Low income**	966 (29.8)	15 (31.3)	1.05 (0.69-1.60)	0.97 (0.61-1.55)	1.5	-0.8	0.059
**Low-medium income**	956 (18.1)	21 (25.3)	1.40 (0.96-2.04)	1.36 (0.97-1.89)	7.2	8.3	--
**Medium income**	637 (13.4)	18 (23.4)	1.75 (1.16-2.64)	1.66 (1.09-2.55)	10.0	12.6	--
**Medium-high income**	316 (9.3)	13 (15.1)	1.63 (0.98-2.72)	2.04 (1.24-3.35)	5.8	13.1	--
**High income**	117 (6.6)	8 (17.3)	2.64 (1.37-5.08)	2.90 (1.56-5.37)	10.8	15.3	--

**Not current smoker**	2351 (14.5)	71 (22.0)	1.52 (1.23-1.87)	1.53 (1.23-1.89)	7.6	8.0	0.234
**Current smoker**	641 (28.3)	4 (22.2)	0.79 (0.33-1.87)	0.91 (0.36-2.33)	-6.1	-1.6	--
**No previous diagnosis of psychiatric disorder**	1053 (8.0)	38 (14.6)	1.83 (1.36-2.47)	1.55 (1.13-2.12)	6.6	5.6	0.176
**Previous diagnosis of psychiatric disorder**	1939 (37.0)	37 (46.8)	1.27 (1.00-1.61)	1.36 (1.04-1.77)	9.9	13.6	--
**No previously diagnosed somatic diseases**	1776 (16.2)	54 (22.4)	1.38 (1.09-1.75)	1.52 (1.19-1.95)	6.2	7.7	0.923
**1 previously diagnosed somatic disease**	785 (14.6)	17 (20.7)	1.42 (0.92-2.18)	1.38 (0.91-2.10)	6.1	6.2	--
**≥ 2 previously diagnosed somatic diseases**	431 (19.8)	4 (23.5)	1.19 (0.50-2.81)	1.88 (0.70-5.06)	3.7	14.7	--
**Response date: April 24-May 7**	2206 (18.3)	45 (27.4)	1.50 (1.16-1.93)	1.62 (1.24-2.11)	9.1	12.6	0.531
**Response date: May 8-July 22**	786 (12.2)	30 (17.0)	1.40 (1.00-1.95)	1.24 (0.89-1.71)	4.9	3.2	--
**Anxiety**	2120 (11.3)	45 (13.1)	1.16 (0.88 to 1.53)	1.24 (0.93 to 1.64)	1.8	2.6	
**Female**	1759 (13.4)	34 (14.8)	1.11 (0.81 to 1.51)	1.17 (0.84 to 1.62)	1.4	2.2	0.007
**Male**	353 (6.2)	10 (8.9)	1.43 (0.78 to 2.60)	1.39 (0.76 to 2.54)	2.6	3.0	--
**18-39 years**	770 (26.2)	13 (14.9)	0.57 (0.34 to 0.95)	0.61 (0.36 to 1.04)	-11.3	-9.3	0.049
**40-59 years**	951 (11.5)	27 (14.7)	1.28 (0.90 to 1.82)	1.70 (1.20 to 2.41)	3.2	10.5	--
**≥60 years**	399 (5.2)	5 (6.9)	1.31 (0.56 to 3.06)	2.13 (0.91 to 5.00)	1.7	7.5	--
**Compulsory education**	378 (14.7)	2 (8.0)	0.54 (0.14-2.06)	0.75 (0.21-2.69)	-6.7	-2.7	0.009
**Upper secondary, vocational or other education**	690 (12.0)	11 (10.5)	0.87 (0.50-1.54)	0.84 (0.48-1.48)	-1.5	-1.4	--
**Bachelor's degree or diploma certificate**	658 (10.9)	17 (13.4)	1.23 (0.79-1.93)	1.18 (0.74-1.86)	2.5	2.0	--
**Master's or Ph.D. degree**	394 (8.9)	15 (17.2)	1.94 (1.21-3.11)	2.47 (1.53-3.99)	8.4	19.6	--
**Low income**	719 (21.6)	6 (12.5)	0.58 (0.27-1.23)	0.52 (0.24-1.12)	-9.1	-8.9	0.082
**Low-medium income**	657 (12.2)	17 (19.8)	1.62 (1.06-2.50)	1.44 (0.95-2.20)	7.6	6.6	--

**Medium income**	432 (8.9)	11 (14.5)	1.62 (0.93-2.82)	1.58 (0.89-2.81)	5.5	6.1	--
**Medium-high income**	223 (6.5)	9 (10.3)	1.60 (0.85-3.01)	2.05 (1.03-4.09)	3.9	8.0	--
**High income**	89 (5.0)	2 (4.3)	0.86 (0.22-3.38)	0.97 (0.25-3.73)	-0.7	-0.1	--
**Not current smoker**	1641 (10.0)	43 (13.2)	1.33 (1.00-1.76)	1.31 (0.98-1.76)	3.3	3.0	0.193
**Current smoker**	479 (20.6)	2 (10.5)	0.51 (0.14-1.90)	0.51 (0.13-1.98)	-10.1	-6.1	--
**No previous diagnosis of psychiatric disorder**	709 (5.3)	24 (9.2)	1.73 (1.18-2.55)	1.46 (0.97-2.19)	3.9	2.5	0.130
**Previous diagnosis of psychiatric disorder**	1411 (26.1)	21 (25.6)	0.98 (0.68-1.42)	1.03 (0.70-1.52)	-0.5	0.8	--
**No previously diagnosed somatic diseases**	1320 (11.9)	34 (13.9)	1.17 (0.85-1.60)	1.22 (0.88-1.71)	2.0	2.2	0.651
**1 previously diagnosed somatic disease**	519 (9.5)	10 (12.3)	1.30 (0.72-2.34)	1.31 (0.71-2.41)	2.9	3.0	--
**≥ 2 previously diagnosed somatic diseases**	281 (12.6)	1 (5.6)	0.44 (0.07-2.97)	0.75 (0.22-2.52)	-7.0	-2.7	--
**Response date: April 24-May 7**	1594 (13.0)	28 (16.7)	1.28 (0.91-1.80)	1.37 (0.97-1.94)	3.7	4.7	0.622
**Response date: May 8-July 22**	526 (8.0)	17 (9.7)	1.21 (0.76-1.91)	0.93 (0.57-1.52)	1.7	-0.6	--
**PTSD**	2699 (15.6)	59 (19.5)	1.25 (0.99 to 1.57)	1.38 (1.09 to 1.75)	3.9	7.2	
**Female**	2198 (18.6)	46 (22.7)	1.22 (0.94 to 1.58)	1.32 (1.02 to 1.71)	4.1	6.7	<.001
**Male**	493 (9.1)	12 (12.1)	1.33 (0.78 to 2.27)	1.60 (0.92 to 2.77)	3.0	7.1	--
**18-39 years**	597 (24.2)	11 (15.3)	0.63 (0.37 to 1.09)	0.77 (0.45 to 1.32)	-8.9	-6.2	0.003
**40-59 years**	1268 (16.7)	31 (19.3)	1.15 (0.84 to 1.59)	1.36 (0.99 to 1.88)	2.6	7.8	--
**≥60 years**	834 (11.6)	17 (24.3)	2.09 (1.38 to 3.18)	2.67 (1.78 to 4.01)	12.7	27.9	--
**Compulsory education**	367 (16.1)	7 (33.3)	2.08 (1.13-3.83)	2.41 (1.19-4.89)	17.2	24.8	0.239
**Upper secondary, vocational or other education**	809 (15.5)	20 (21.1)	1.36 (0.92-2.02)	1.44 (0.96-2.18)	5.6	9.1	--
**Bachelor's degree or diploma certificate**	912 (16.4)	23 (21.1)	1.29 (0.89-1.86)	1.34 (0.93-1.94)	4.7	4.8	--

**Master's or Ph.D. degree**	611 (14.7)	9 (11.5)	0.79 (0.42-1.46)	1.01 (0.55-1.84)	-3.1	0.2	--
**Low income**	602 (21.0)	10 (22.7)	1.08 (0.62-1.87)	1.11 (0.64-1.93)	1.7	2.2	0.756
**Low-medium income**	773 (15.8)	17 (23.6)	1.49 (0.98-2.27)	1.53 (0.98-2.37)	7.8	9.9	--
**Medium income**	726 (16.1)	12 (17.4)	1.08 (0.64-1.82)	1.16 (0.68-1.98)	1.3	2.8	--
**Medium-high income**	403 (12.3)	14 (17.9)	1.46 (0.90-2.36)	1.77 (1.14-2.76)	5.6	16.4	--
**High income**	195 (11.4)	6 (15.0)	1.32 (0.62-2.79)	1.53 (0.72-3.26)	3.6	6.7	--
**Not current smoker**	2244 (14.7)	51 (17.9)	1.22 (0.95-1.56)	1.28 (0.99-1.65)	3.2	4.5	0.054
**Current smoker**	455 (22.6)	8 (44.4)	1.97 (1.17-3.32)	2.83 (1.57-5.10)	21.9	37.4	--
**No previous diagnosis of psychiatric disorder**	1540 (12.2)	42 (17.6)	1.45 (1.10-1.92)	1.55 (1.17-2.06)	5.5	7.5	0.137
**Previous diagnosis of psychiatric disorder**	1159 (25.3)	17 (26.2)	1.04 (0.69-1.56)	1.11 (0.73-1.68)	0.9	3.0	--
**No previously diagnosed somatic diseases**	1611 (15.8)	41 (19.4)	1.23 (0.93-1.62)	1.44 (1.09-1.90)	3.6	7.6	0.325
**1 previously diagnosed somatic disease**	751 (14.8)	12 (16.0)	1.08 (0.64-1.82)	1.11 (0.66-1.87)	1.2	1.8	--
**≥ 2 previously diagnosed somatic diseases**	337 (16.7)	6 (35.3)	2.11 (1.10-4.04)	2.03 (0.94-4.40)	18.6	17.5	--
**Response date: April 24-May 7**	1996 (18.1)	32 (22.5)	1.25 (0.92-1.70)	1.24 (0.91-1.70)	4.5	5.8	0.256
**Response date: May 8-July 22**	703 (11.4)	27 (16.8)	1.47 (1.04-2.09)	1.41 (0.99-2.01)	5.4	6.0	--

[a] Adjusted for age (continuous variable), gender (male, female, or other), educational level (compulsory education, high school/trade school/other education, bachelor's degree/diploma certificate, or master's/Ph.D. degree), income (continuous variable), current smoking (yes or no), previous diagnosis of a psychiatric disorder (yes or no), number of previously diagnosed somatic diseases (0, 1, or ≥ 2), and timing of responding to the baseline questionnaire (April 24-30, May 1-7, May 8-23, or May 24-July 22). Adjustments are not made for the variable that is being stratified by.

Supplementary Table 3. The prevalence, crude and multivariable adjusted relative risks and absolute differences in symptoms of depression (PHQ-9), anxiety (GAD-7) and PTSD (PC-PTSD-5) surpassing screening thresholds among individuals with and without a confirmed diagnosis of COVID-19 (*diagnosed at least two weeks before responding)*.

	No. (%)					
	Individuals not diagnosed with COVID-19	Individuals diagnosed with COVID-19	cRR (95% CI)	aRR (95% CI)[a]	Absolute difference Crude %	Absolute difference Adjusted[a] %
**Depression**	2992 (16.2)	73 (22.0)	1.36 (1.11 to 1.67)	1.49 (1.20 to 1.84)	5.8	8.6
**Female**	2421 (18.8)	55 (24.9)	1.32 (1.05 to 1.67)	1.45 (1.14 to 1.83)	6.1	8.4
**Male**	559 (10.0)	16 (14.7)	1.47 (0.93 to 2.33)	1.52 (0.93 to 2.49)	4.7	6.9
**18-39 years**	954 (33.3)	22 (26.8)	0.81 (0.56 to 1.16)	0.95 (0.65 to 1.39)	-6.5	-1.5
**40-59 years**	1367 (16.8)	39 (22.3)	1.32 (1.00 to 1.75)	1.72 (1.30 to 2.28)	5.5	14.7
**≥60 years**	671 (8.9)	12 (16.0)	1.79 (1.06 to 3.02)	2.59 (1.53 to 4.39)	7.1	22.0
**Anxiety**	2120 (11.3)	45 (13.1)	1.16 (0.88 to 1.54)	1.25 (0.94 to 1.67)	1.8	2.9
**Female**	1759 (13.4)	33 (14.7)	1.10 (0.80 to 1.51)	1.17 (0.84 to 1.64)	1.3	2.3
**Male**	353 (6.2)	10 (9.1)	1.46 (0.80 to 2.67)	1.46 (0.80 to 2.64)	2.9	3.5
**18-39 years**	770 (26.2)	13 (15.5)	0.59 (0.36 to 0.98)	0.64 (0.38 to 1.09)	-10.7	-8.6
**40-59 years**	951 (11.5)	26 (14.4)	1.26 (0.88 to 1.80)	1.68 (1.18 to 2.41)	2.9	10.2
**≥60 years**	399 (5.2)	5 (6.9)	1.32 (0.57 to 3.10)	2.15 (0.91 to 5.03)	1.7	7.5
**PTSD**	2699 (15.6)	56 (18.9)	1.21 (0.95 to 1.54)	1.35 (1.06 to 1.73)	3.3	6.7
**Female**	2198 (18.6)	43 (21.7)	1.17 (0.89 to 1.53)	1.27 (0.97 to 1.67)	3.1	5.7
**Male**	493 (9.1)	12 (12.4)	1.36 (0.79 to 2.32)	1.64 (0.95 to 2.83)	3.3	7.5
**18-39 years**	597 (24.2)	10 (14.3)	0.59 (0.33 to 1.05)	0.72 (0.41 to 1.28)	-9.9	-7.4
**40-59 years**	1268 (16.7)	29 (18.5)	1.11 (0.79 to 1.54)	1.31 (0.94 to 1.84)	1.8	6.7
**≥60 years**	834 (11.6)	17 (24.6)	2.12 (1.40 to 3.23)	2.70 (1.80 to 4.05)	13.0	28.3

[a] Adjusted for age (continuous variable), gender (male, female, or other), educational level (compulsory education, high school/trade school/other education, bachelor's degree/diploma certificate, or master's/Ph.D. degree), income (continuous variable), current smoking (yes or no), previous diagnosis of a psychiatric disorder (yes or no), number of previously diagnosed somatic diseases (0, 1, or ≥ 2), and timing of responding to the baseline questionnaire (April 24-30, May 1-7, May 8-23, or May 24-July 22). Adjustments are not made for the variable that is being stratified by.

Supplementary Table 4. The prevalence, crude and multivariable adjusted relative risks and absolute differences in symptoms of depression (PHQ-9), anxiety (GAD-7) and PTSD (PC-PTSD-5) surpassing screening thresholds among individuals with and without a confirmed diagnosis of COVID-19 (*using multiple imputation*).

	No. (%)					
	Individuals not diagnosed with COVID-19	Individuals diagnosed with COVID-19	cRR (95% CI)	aRR (95% CI)[a]	Absolute difference Crude %	Absolute difference Adjusted [a] %
**Depression**	3487 (17.0)	83 (22.3)	1.31 (1.08 to 1.59)	1.44 (1.18 to 1.75)	5.3	8.1
**Female**	2819 (19.7)	64 (25.6)	1.29 (1.05 to 1.61)	1.43 (1.14 to 1.78)	5.9	8.3
**Male**	655 (10.7)	17 (14.1)	1.32 (0.85 to 2.08)	1.35 (0.85 to 2.16)	3.4	4.6
**18-39 years**	1105 (35.2)	26 (28.9)	0.82 (0.59 to 1.14)	0.95 (0.67 to 1.33)	-6.3	-1.8
**40-59 years**	1565 (17.5)	445(22.5)	1.29 (0.99 to 1.68)	1.68 (1.29 to 2.18)	5.0	14.8
**≥60 years**	817 (9.7)	12 (14.8)	1.52 (0.90 to 2.58)	2.23 (1.31 to 3.78)	5.1	17.2
**Anxiety**	2328 (11.3)	45 (12.1)	1.06 (0.81 to 1.40)	1.15 (0.87 to 1.53)	0.8	1.8
**Female**	1940 (13.5)	34 (13.6)	1.01 (0.73 to 1.38)	1.08 (0.78 to 1.50)	0.1	1.0
**Male**	378 (6.2)	10 (8.3)	1.34 (0.73 to 2.45)	1.34 (0.74 to 2.43)	2.1	2.6
**18-39 years**	830 (26.5)	13 (14.4)	0.55 (0.33 to 0.91)	0.61 (0.36 to 1.03)	-12.1	-10.0
**40-59 years**	1037 (11.6)	27 (13.6)	1.18 (0.83 to 1.68)	1.57 (1.11 to 2.23)	2.0	9.0
**≥60 years**	461 (5.5)	5 (5.9)	1.08 (0.46 to 2.53)	1.69 (0.72 to 3.97)	0.4	4.6
**PTSD**	2915 (15.8)	62 (19.2)	1.22 (0.97 to 1.53)	1.36 (1.08 to 1.71)	3.4	6.7
**Female**	2375 (18.7)	48 (22.1)	1.18 (0.92 to 1.52)	1.29 (1.00 to 1.66)	3.4	6.1
**Male**	532 (9.2)	13 (12.4)	1.34 (0.80 to 2.25)	1.57 (0.93 to 2.67)	3.2	6.9
**18-39 years**	642 (24.5)	11 (14.9)	0.61 (0.35 to 1.05)	0.71 (0.41 to 1.22)	-9.6	-7.9
**40-59 years**	1360 (16.8)	33 (19.3)	1.15 (0.84 to 1.57)	1.37 (1.00 to 1.87)	2.5	7.9
**≥60 years**	913 (11.8)	18 (23.1)	1.96 (1.30 to 2.96)	2.47 (1.65 to 3.69)	11.3	23.9

[a] Adjusted for age (continuous variable), gender (male, female, or other), educational level (compulsory education, high school/trade school/other education, bachelor's degree/diploma certificate, or master's/Ph.D. degree), income (continuous variable), current smoking (yes or no), previous diagnosis of a psychiatric disorder (yes or no), number of previously diagnosed somatic diseases (0, 1, or ≥ 2), and timing of responding to the baseline questionnaire (April 24-30, May 1-7, May 8-23, or May 24-July 22). Adjustments are not made for the variable that is being stratified by.

Supplementary Table 5. The prevalence, crude and multivariable adjusted relative risks and absolute differences in symptoms of depression (PHQ-9), anxiety (GAD-7) and PTSD (PC-PTSD-5) surpassing screening thresholds among individuals with and without a confirmed diagnosis of COVID-19 (*analysis limited to individuals who were tested for COVID-19*).

	No. (%)					
	Individuals that have been tested for COVID-19 but not diagnosed	Individuals that have been tested for COVID-19 and diagnosed	cRR (95% CI)	aRR (95% CI)[a]	Absolute difference Crude %	Absolute difference Adjusted [a] %
**Depression**	885 (19.4)	75 (22.1)	1.14 (0.92 to 1.40)	1.31 (1.06 to 1.62)	2.7	6.1
**Female**	712 (21.7)	56 (24.8)	1.14 (0.90 to 1.45)	1.30 (1.02 to 1.65)	3.1	6.2
**Male**	168 (13.2)	17 (15.2)	1.15 (0.72 to 1.82)	1.22 (0.75 to 1.97)	2.0	3.7
**18-39 years**	275 (37.6)	23 (27.1)	0.72 (0.50 to 1.03)	0.85 (0.58 to 1.23)	-10.5	-5.2
**40-59 years**	413 (17.8)	40 (22.3)	1.26 (0.94 to 1.68)	1.64 (1.23 to 2.17)	4.5	13.6
**≥60 years**	197 (13.1)	12 (15.8)	1.21 (0.71 to 2.07)	1.86 (1.09 to 3.18)	2.7	16.4
**Anxiety**	640 (13.8)	45 (13.1)	0.95 (0.72 to 1.26)	1.08 (0.81 to 1.44)	-0.7	1.1
**Female**	531 (15.9)	34 (14.8)	0.94 (0.68 to 1.29)	1.04 (0.75 to 1.45)	-1.1	0.6
**Male**	106 (8.2)	10 (8.9)	1.08 (0.58 to 2.01)	1.11 (0.60 to 2.04)	0.7	1.0
**18-39 years**	237 (31.3)	13 (14.9)	0.48 (0.29 to 0.80)	0.52 (0.31 to 0.89)	-16.4	-13.4
**40-59 years**	286 (12.1)	27 (14.7)	1.21 (0.84 to 1.75)	1.62 (1.13 to 2.31)	2.6	9.7
**≥60 years**	117 (7.7)	5 (6.9)	0.90 (0.38 to 2.12)	1.56 (0.66 to 3.70)	-0.8	5.1
**PTSD**	747 (17.8)	59 (19.5)	1.09 (0.86 to 1.39)	1.26 (0.99 to 1.60)	1.7	5.4
**Female**	626 (21.1)	46 (22.7)	1.08 (0.83 to 1.40)	1.19 (0.91 to 1.56)	1.6	4.5
**Male**	119 (9.7)	12 (12.1)	1.25 (0.71 to 2.17)	1.52 (0.86 to 2.67)	2.4	6.5
**18-39 years**	168 (27.6)	11 (15.3)	0.55 (0.32 to 0.97)	0.68 (0.39 to 1.18)	-12.3	-9.6
**40-59 years**	376 (17.4)	31 (19.3)	1.11 (0.80 to 1.54)	1.31 (0.94 to 1.83)	1.9	6.9
**≥60 years**	203 (14.2)	17 (24.3)	1.70 (1.11 to 2.63)	2.22 (1.46 to 3.37)	10.1	24.6

[a] Adjusted for age (continuous variable), gender (male, female, or other), educational level (compulsory education, high school/trade school/other education, bachelor's degree/diploma certificate, or master's/Ph.D. degree), income (continuous variable), current smoking (yes or no), previous diagnosis of a psychiatric disorder (yes or no), number of previously diagnosed somatic diseases (0, 1, or ≥ 2), and timing of responding to the baseline questionnaire (April 24-30, May 1-7, May 8-23, or May 24-July 22). Adjustments are not made for the variable that is being stratified by.

Supplementary Table 6. Raw prevalence of flu-like symptoms during the preceding two months among individuals with and without a recent COVID-19 diagnosis.

	No. (%)		
	**Individuals not diagnosed with COVID-19 (n = 20488)**	**Individuals diagnosed with COVID-19 (n = 373)**	**P-value**
**No reported symptoms**			
Yes	3023 (14.8)	12 (3.2)	<.001
No	17465 (85.2)	361 (96.8)	
**Cough**			
Never	13181 (64.3)	122 (32.7)	<.001
1-2 days	3658 (17.9)	57 (15.3)	
3-6 days	1734 (8.5)	55 (14.7)	
1-2 weeks	754 (3.7)	54 (14.5)	
Over 2 weeks	1161 (5.7)	85 (22.8)	
**Fatigue and weakness**			
Never	7535 (36.8)	32 (8.6)	<.001
1-2 days	6044 (29.5)	39 (10.5)	
3-6 days	3350 (16.4)	54 (14.5)	
1-2 weeks	1276 (6.2)	59 (15.8)	
Over 2 weeks	2283 (11.1)	189 (50.7)	
**Fever**			
Never	17362 (84.7)	127 (34.0)	<.001
1-2 days	1907 (9.3)	58 (15.5)	
3-6 days	831 (4.1)	74 (19.8)	
1-2 weeks	227 (1.1)	53 (14.2)	
Over 2 weeks	161 (0.8)	61 (16.4)	
**Gastrointestinal symptoms**			
Never	12589 (61.4)	162 (43.4)	<.001
1-2 days	4565 (22.3)	69 (18.5)	
3-6 days	1941 (9.5)	61 (16.4)	
1-2 weeks	585 (2.9)	42 (11.3)	
Over 2 weeks	808 (3.9)	39 (10.5)	
**Headache**			
Never	8583 (41.9)	73 (19.6)	<.001
1-2 days	6754 (33.0)	57 (15.3)	
3-6 days	3381 (16.5)	95 (25.5)	
1-2 weeks	816 (4.0)	62 (16.6)	
Over 2 weeks	954 (4.7)	86 (23.1)	

	No. (%)		
	**Individuals not diagnosed with COVID-19 (n = 20488)**	**Individuals diagnosed with COVID-19 (n = 373)**	**P-value**
**Impaired sense of taste and/or smell**			
Never	19514 (95.2)	140 (37.5)	<.001
1-2 days	411 (2.0)	25 (6.7)	
3-6 days	220 (1.1)	44 (11.8)	
1-2 weeks	111 (0.5)	55 (14.7)	
Over 2 weeks	232 (1.1)	109 (29.2)	
**Myalgia**			
Never	12253 (59.8)	105 (28.2)	<.001
1-2 days	3512 (17.1)	54 (14.5)	
3-6 days	2072 (10.1)	72 (19.3)	
1-2 weeks	708 (3.5)	81 (21.7)	
Over 2 weeks	1943 (9.5)	61 (16.4)	
**Shortness of breath**			
Never	17111 (83.5)	169 (45.3)	<.001
1-2 days	1809 (8.8)	42 (11.3)	
3-6 days	775 (3.8)	55 (14.7)	
1-2 weeks	312 (1.5)	41 (11.0)	
Over 2 weeks	481 (2.3)	66 (17.7)	
**Sore throat**			
Never	12987 (63.4)	153 (41.0)	<.001
1-2 days	4323 (21.1)	62 (16.6)	
3-6 days	1901 (9.3)	63 (16.9)	
1-2 weeks	632 (3.1)	39 (10.5)	
Over 2 weeks	645 (3.1)	56 (15.0)	

Supplementary Table 7. Prevalence, crude and adjusted relative risks with 95% confidence intervals of flu-like symptoms lasting for 1 week or more, during the preceding two months, among individuals who tested negative for SARS-CoV-2 and those who were never tested.

Symptoms	No. (%) Individuals not tested for SARS-CoV-2 (n = 15442)	No. (%) Individuals tested negative for SARS-CoV-2 (n = 5046)	cRR (95% CI)	aRR (95% CI)[a]
Cough	1249 (8.1)	666 (13.2)	1.60 (1.46 to 1.76)	1.59 (1.45 to 1.75)
Fatigue and weakness	2420 (15.7)	1139 (22.3)	1.45 (1.36 to 1.55)	1.37 (1.29 to 1.46)
Fever	176 (1.1)	212 (4.2)	3.71 (3.02 to 4.56)	3.49 (2.84 to 4.29)
Gastrointestinal symptoms	946 (6.1)	447 (8.9)	1.48 (1.33 to 1.66)	1.37 (1.23 to 1.53)
Headache	1134 (7.3)	636 (12.6)	1.74 (1.58 to 1.91)	1.60 (1.46 to 1.75)
Impaired sense of taste or smell	229 (1.5)	114 (2.3)	1.53 (1.21 to 1.93)	1.64 (1.30 to 2.07)
Myalgia	1881 (12.2)	770 (15.3)	1.26 (1.16 to 1.36)	1.29 (1.19 to 1.39)
Shortness of breath	503 (3.3)	290 (5.7)	1.88 (1.62 to 2.17)	1.84 (1.59 to 2.13)
Sore throat	778 (5.0)	499 (9.9)	1.98 (1.77 to 2.21)	1.91 (1.71 to 2.14)

[a] Adjusted for age (continuous variable), gender (male, female, or other), educational level (compulsory education, high school/trade school/other education, bachelor's degree/diploma certificate, or master's/Ph.D. degree), income (continuous variable), current smoking (yes or no), previous diagnosis of a psychiatric disorder (yes or no), number of previously diagnosed somatic diseases (0, 1, or ≥ 2), and timing of responding to the baseline questionnaire (April 24-30, May 1-7, May 8-23, or May 24-July 22).

Supplementary Table 8. Prevalence, crude and adjusted relative risks with 95% confidence intervals of flu-like symptoms lasting for 1 week or more, during the preceding two months, among individuals with and without a confirmed diagnosis of COVID-19.

	**No. (%)**			
**Symptoms**	**Individuals not diagnosed with COVID-19 (n = 20488)**	**Individuals diagnosed with COVID-19 (n = 373)**	**cRR (95% CI)**	**aRR (95% CI)[a]**
Cough	1915 (9.4)	139 (37.3)	4.15 (3.60 to 4.76)	5.02 (4.35 to 5.81)
Fatigue and weakness	3559 (17.4)	248 (66.5)	3.93 (3.63 to 4.26)	4.16 (3.77 to 4.60)
Fever	388 (1.9)	114 (30.6)	16.77 (13.93 to 20.19)	19.10 (15.49 to 23.48)
Gastrointestinal symptoms	1393 (6.8)	81 (21.7)	3.29 (2.69 to 4.06)	3.49 (2.82 to 4.32)
Headache	1770 (8.6)	148 (39.7)	4.67 (4.10 to 5.37)	4.64 (3.99 to 5.39)
Impaired sense of taste or smell	343 (1.7)	164 (44.0)	27.04 (23.10 to 31.82)	32.52 (27.22 to 38.86)
Myalgia	2651 (12.9)	142 (38.1)	3.03 (2.65 to 3.49)	3.66 (3.16 to 4.23)
Shortness of breath	793 (3.9)	107 (28.7)	7.53 (6.05 to 9.03)	9.26 (7.61 to 11.25)
Sore throat	1277 (6.2)	95 (25.5)	4.17 (3.46 to 5.00)	4.58 (3.78 to 5.54)

[a] Adjusted for age (continuous variable), gender (male, female, or other), educational level (compulsory education, high school/trade school/other education, bachelor's degree/diploma certificate, or master's/Ph.D. degree), income (continuous variable), current smoking (yes or no), previous diagnosis of a psychiatric disorder (yes or no), number of previously diagnosed somatic diseases (0, 1, or ≥ 2), and timing of responding to the baseline questionnaire (April 24-30, May 1-7, May 8-23, or May 24-July 22).

Supplementary Table 9. Adjusted mean symptom scores of depression, anxiety and PTSD by flu-like symptom burden in tertiles among individuals with a recent COVID-19 diagnosis, those who tested negative and those never tested for SARS-CoV-2.

	Multivariable adjusted mean value by tertile of flu-like symptoms[a]				
	Lowest tertile	Mid tertile	Highest tertile	Beta (95% CI)	P-value
**Depression**					
Patients with COVID-19	5.49	7.30	10.89	0.24 (0.17 to 0.30)	<.001
Tested negative for SARS-CoV-2	6.33	9.48	10.75	0.29 (0.25 to 0.33)	<.001
Not tested for SARS-CoV-2	6.04	9.79	11.34	0.34 (0.33 to 0.36)	<.001
**Anxiety**					
Patients with COVID-19	4.31	5.29	7.02	0.12 (0.06 to 0.17)	<.001
Tested negative for SARS-CoV-2	5.06	7.16	8.02	0.19 (0.16 to 0.23)	.03
Not tested for SARS-CoV-2	4.81	7.04	8.17	0.22 (0.20 to 0.23)	<.001
**PTSD**					
Patients with COVID-19	1.85	2.26	2.50	0.03 (0.01 to 0.05)	<.001
Tested negative for SARS-CoV-2	1.88	2.43	2.21	0.05 (0.04 to 0.06)	.02
Not tested for SARS-CoV-2	1.82	2.31	2.59	0.06 (0.06 to 0.07)	<.001

[a] Adjusted for age (continuous variable), gender (male, female, or other), educational level (compulsory education, high school/trade school/other education, bachelor's degree/diploma certificate, or master's/Ph.D. degree), income (continuous variable), current smoking (yes or no), previous diagnosis of a psychiatric disorder (yes or no), number of previously diagnosed somatic diseases (0, 1, or ≥ 2), and timing of responding to the baseline questionnaire (April 24-30, May 1-7, May 8-23, or May 24-July 22).
