# Supplementary material for: Illness severity and risk of mental morbidities among patients recovering from COVID-19: a cross-sectional study in the Icelandic population
Source: BMJ Open. 2021 Jul 23;11(7):e049967. doi: 10.1136/bmjopen-2021-049967 (PMC8313306; doi:10.1136/bmjopen-2021-049967)
Supplement: Author's manuscript [file bmjopen-2021-049967.draft_revisions.pdf]

BMJ Open is committed to open peer review. As part of this commitment we make the peer review history of every article we publish publicly available.

When an article is published we post the peer reviewers' comments and the authors' responses online. We also post the versions of the paper that were used during peer review. These are the versions that the peer review comments apply to.

The versions of the paper that follow are the versions that were submitted during the peer review process. They are not the versions of record or the final published versions. They should not be cited or distributed as the published version of this manuscript.

BMJ Open is an open access journal and the full, final, typeset and author-corrected version of record of the manuscript is available on our site with no access controls, subscription charges or pay-per-view fees (http://bmjopen.bmj.com).

If you have any questions on BMJ Open's open peer review process please email info.bmjopen@bmj.com

BMJ Open

Illness severity and risk of mental morbidities among patients recovering from COVID-19: a cross-sectional study in the Icelandic population

Journal:	*BMJ Open*
Manuscript ID	bmjopen-2021-049967
Article Type:	Original research
Date Submitted by the Author:	14-Feb-2021

Complete List of Authors:	Sævarsdóttir, Karen ; University of Iceland School of Health Sciences, Centre of Public Health Sciences, Faculty of Medicine<br>Hilmarsdóttir, Hildur; University of Iceland School of Health Sciences, Centre of Public Health Sciences, Faculty of Medicine<br>Magnúsdóttir, Ingibjörg; University of Iceland School of Health Sciences, Centre of Public Health Sciences, Faculty of Medicine<br>Hauksdóttir, Arna; University of Iceland School of Health Sciences, Centre of Public Health Sciences, Faculty of Medicine<br>Thordardottir, Edda; University of Iceland School of Health Sciences, Center of Public Health Sciences, Faculty of Medicine<br>Guðjónsdóttir, Ásdís; University of Iceland School of Health Sciences, Center of Public Health Sciences, Faculty of Medicine<br>Tomasson , Gunnar ; University of Iceland School of Health Sciences, Center of Public Health Sciences, Faculty of Medicine; Landspitali<br>Rúnarsdóttir, Harpa; University of Iceland School of Health Sciences, Center of Public Health Sciences, Faculty of Medicine<br>Jónsdóttir, Harpa; University of Iceland School of Health Sciences, Center of Public Health Sciences, Faculty of Medicine<br>Guðmundsdóttir, Berglind; University of Iceland School of Health Sciences, Faculty of Medicine<br>Pétursdóttir, Guðrún; University of Iceland, Institute for Sustainability Studies<br>Petersen, Pétur; University of Iceland School of Health Sciences, Faculty of Medicine<br>Kristinsson, Sigurður; University of Iceland School of Health Sciences, Faculty of Medicine<br>Love, Thorvardur ; University of Iceland School of Health Sciences, Faculty of Medicine; Landspitali<br>Hansdóttir, Sif; Landspitali<br>Harðardóttir, Hrönn; Landspitali<br>Gudmundsson, Gunnar; University of Iceland School of Health Sciences, Faculty of Medicine; Landspitali<br>Eythorsson, Elias; Landspitali<br>Guðmundsdóttir, Dóra ; The Directorate of Health<br>Sigbjörnsdóttir, Hildur; The Directorate of Health<br>Haraldsdóttir, Sigríður; The Directorate of Health<br>Möller, Alma; The Directorate of Health<br>Palsson, Runolfur; Landspitali; University of Iceland School of Health Sciences, Faculty of Medicine

Jakobsdóttir, Jóhanna; University of Iceland School of Health Sciences, Center of Public Health Sciences, Faculty of Medicine
Aspelund, Thor ; University of Iceland School of Health Sciences, Center of Public Health Sciences, Faculty of Medicine; Icelandic Heart Association
Valdimarsdottir, Unnur; University of Iceland School of Health Sciences, Center of Public Health Sciences, Faculty of Medicine; Karolinska Institute, Department of Medical Epidemiology and Biostatistics

Keywords: COVID-19, Epidemiology < INFECTIOUS DISEASES, Public health < INFECTIOUS DISEASES, MENTAL HEALTH

SCHOLARONE™
Manuscripts

I, the Submitting Author has the right to grant and does grant on behalf of all authors of the Work (as defined in the below author licence), an exclusive licence and/or a non-exclusive licence for contributions from authors who are: i) UK Crown employees; ii) where BMJ has agreed a CC-BY licence shall apply, and/or iii) in accordance with the terms applicable for US Federal Government officers or employees acting as part of their official duties; on a worldwide, perpetual, irrevocable, royalty-free basis to BMJ Publishing Group Ltd ("BMJ") its licensees and where the relevant Journal is co-owned by BMJ to the co-owners of the Journal, to publish the Work in this journal and any other BMJ products and to exploit all rights, as set out in our licence.

The Submitting Author accepts and understands that any supply made under these terms is made by BMJ to the Submitting Author unless you are acting as an employee on behalf of your employer or a postgraduate student of an affiliated institution which is paying any applicable article publishing charge ("APC") for Open Access articles. Where the Submitting Author wishes to make the Work available on an Open Access basis (and intends to pay the relevant APC), the terms of reuse of such Open Access shall be governed by a Creative Commons licence – details of these licences and which Creative Commons licence will apply to this Work are set out in our licence referred to above.

Other than as permitted in any relevant BMJ Author's Self Archiving Policies, I confirm this Work has not been accepted for publication elsewhere, is not being considered for publication elsewhere and does not duplicate material already published. I confirm all authors consent to publication of this Work and authorise the granting of this licence.

For peer review only

Illness severity and risk of mental morbidities among patients recovering from COVID-19: a cross-sectional study in the Icelandic population

Karen Sól Sævarsdóttir,*[1] medical student, Hildur Ýr Hilmarsdóttir,*[1] psychology student, Ingibjörg Magnúsdóttir,*[1] biostatistics student, Arna Hauksdóttir,[1] professor of epidemiology, Edda Bjork Thordardottir,[1] postdoctoral fellow of epidemiology, Ásdís Braga Guðjónsdóttir,[1] physician, Gunnar Tómasson,[1,2] consultant in reumatology and associate professor of medicine, Harpa Rúnarsdóttir,[1] data manager, Harpa Lind Jónsdóttir,[1] postdoctoral fellow of epidemiology, Berglind Guðmundsdóttir,[2] professor of psychology, Guðrún Pétursdóttir,[3] associate professor of physiology and director of Saemundur Frodi Center for Sustainability Studies, Pétur Henry Petersen,[2] professor of medicine, Sigurður Yngvi Kristinsson,[2] consultant in haematology and professor of medicine, Thorvardur Jon Love,[2,4] consultant of rheumatology and professor of medicine, Sif Hansdóttir,[4] consultant in pulmonology, Hrönn Harðardóttir,[4] consultant in pulmonology, Gunnar Gudmundsson,[2,4] consultant in pulmonology and professor of medicine, Elias Eythorsson,[4] internal medicine trainee, Dóra Guðrún Guðmundsdóttir,[5] director of health promotion, Hildur Sigbjörnsdóttir,[5] data manager, Sigríður Haralds Elínardóttir,[5] director of health statistics, Alma D. Möller,[5] surgeon general, Runólfur Pálsson,[2,4] professor of medicine and director of internal medicine and rehabilitation services, Jóhanna Jakobsdóttir,*[1] assistant professor of biostatistics, Thor Aspelund,*[1,6] professor of biostatistics, and Unnur Valdimarsdottir,*[1,7,8] professor of epidemiology

*Equal contribution.

[1]Center of Public Health Sciences, Faculty of Medicine, School of Health Sciences, University of Iceland, Reykjavik, Iceland.

[2]Faculty of Medicine, School of Health Sciences, University of Iceland, Reykjavik, Iceland.

[3]Institute of Sustainability Studies, University of Iceland, Reykjavik, Iceland.

For peer review only - http://bmjopen.bmj.com/site/about/guidelines.xhtml

[4]Landspitali – The National University Hospital of Iceland, Reykjavik, Iceland.

[5]The Directorate of Health, Reykjavik, Iceland.

[6]The Icelandic Heart Association, Reykjavik, Iceland.

[7]Department of Medical Epidemiology and Biostatistics, Karolinska Institutet, Stockholm, Sweden.

[8]Department of Epidemiology, Harvard TH Chan School of Public Health, Boston, Massachusetts, USA.

Correspondence to:

Prof. Unnur Anna Valdimarsdóttir, unnurav@hi.is, (ORCID 0000-0001-5382-946X)

Center of Public Health Sciences, Faculty of Medicine, School of Health Sciences, University of Iceland, Sturlugata 8, 101 Reykjavik, Iceland.

Word count (manuscript): 3069

For peer review only

Abstract

Objective

To test if patients recovering from COVID-19 are at increased risk of mental morbidities and to what extent such risk is exacerbated by illness severity.

Design

Population-based cross-sectional study.

Setting

Iceland.

Participants

A total of 22,861 individuals were recruited through invitations to existing nationwide cohorts and a social media campaign from April 24 to July 22 2020, of which 373 were patients recovering from COVID-19.

Main outcome measures

Symptoms of depression (Patient Health Questionnaire [PHQ-9]), anxiety (General Anxiety Disorder Scale [GAD-7]), and PTSD (modified Primary Care PTSD Screen for DSM-5 [PC-PTSD-5]) above screening thresholds. Adjusting for multiple covariates and comorbidities, multivariable Poisson regression was used to assess the association between COVID-19 severity and mental morbidities.

Results

Compared to individuals without a diagnosis of COVID-19, patients recovering from COVID-19 had increased risk of depression (22.1% vs. 16.2%; aRR 1.48, 95% CI 1.20 to 1.82) and PTSD (19.5% vs.

For peer review only

15.6%; aRR 1.38, 95% CI 1.09 to 1.75) but not anxiety (13.1% vs. 11.3%; aRR 1.24, 95% CI 0.93 to 1.64). Elevated relative risks were limited to recovering COVID-19 patients 40 years or older and were particularly high among patients 60 years or older. Among recovering COVID-19 patients, symptoms of depression were particularly common among those in the highest, compared to the lowest tertile of flu-like symptom burden (47.1% vs. 5.8%; aRR 6.42, 95% CI 2.77 to 14.87), among patients confined to bed for seven days or longer compared to those never confined to bed (33.3% vs. 10.9%; aRR 3.67, 95% CI 1.97 to 6.86), and among patients hospitalized for COVID-19 compared to those never admitted to hospital (48.1% vs. 19.9%; aRR 2.72, 95% CI 1.67 to 4.44).

Conclusions

Severe disease course is associated with increased risk of depression and PTSD among patients recovering from COVID-19.

For peer review only

Strengths and limitations of this study

- To our knowledge, this is the first study addressing mental morbidities among recovering COVID-19 patients in a general population setting, including outpatients.
- This study includes more than 20% of all patients diagnosed with COVID-19 by RT-PCR during the first wave of the epidemic in Iceland, where the diagnostic rate was among the highest worldwide, allowing us to perform sensitivity analyses limited to individuals who all had been screened for SARS-CoV-2.
- The extensive and well-piloted questionnaire allowed us to obtain validated symptom assessment of mental morbidities along with detailed information on flu-like symptom burden for all individuals during the flu season in Iceland.
- The cross-sectional design is less informative on the directionality of the studied associations, and as the study is nested within a small, economically and socially secure society, the generalizability of the findings may be limited.
- It is possible that individuals with severe symptoms of depression and anxiety were more likely to report high levels of flu-like symptoms during the preceding two months when responding to the questionnaire, yielding a outcome-dependent misclassification.

For peer review only

For peer review only - http://bmjopen.bmj.com/site/about/guidelines.xhtml

INTRODUCTION

With almost 100 million reported cases and 2.2 million deaths globally,[1] the coronavirus disease 2019 (COVID-19) pandemic remains a major public health challenge worldwide. Beyond the extensive socioeconomic impact and suffering of patients during the illness, there are rising concerns about long-term somatic and psychological impact on recovering patients.[2-6]

Mental health consequences among patients in recent epidemics are well documented.[7, 8] With respect to COVID-19, a meta-analysis of 31 studies suggested a 45-47% pooled prevalence of mild to severe symptoms of depression and anxiety in patients with COVID-19, mostly inpatients.[9] Moreover, extreme rates of posttraumatic stress have been reported among COVID-19 inpatients in China.[10, 11] Previous studies are mostly small, limited to inpatients, and rarely include a control group. Few studies have reported symptoms of mental morbidities after recovery from COVID-19 with existing data suggesting high levels of symptoms[12] and elevated rates of diagnosed psychiatric disorders among COVID-19 inpatients during the first weeks after hospital discharge.[13] Flu-like symptoms, such as myalgia, cough and sore throat, have been associated with mental morbidities in the general population during the COVID-19 pandemic.[14, 15] To what extent flu-like symptoms are associated with psychological distress in recently recovered COVID-19 patients remains unknown.

The first wave of COVID-19 in Iceland started on February 28. Through modest but systematic mitigation strategies[16] the incidence of COVID-19 had effectively been attenuated by the end of April 2020, with only sporadic cases occurring until the onset of the second wave of infections on July 22. During this period, more than 20% of the total population was tested using reverse transcription-polymerase chain reaction (RT-PCR); a total of 1820 tested positive for SARS-CoV-2 (0.5% of the total population), of whom 113 were hospitalized (6%), and ten died (0.5%).[17] Here

For peer review only - http://bmjopen.bmj.com/site/about/guidelines.xhtml

we explore mental health indicators in patients recovering from COVID-19 in the first wave of the pandemic and the potential role of disease severity on the risk of mental morbidities.

METHODS

Study population and design

The COVID-19 National Resilience Cohort was established on April 24, 2020, with the overarching aim of understanding the long-term public health impact of the COVID-19 epidemic in Iceland.[18] Eligible were all Icelandic and English speaking individuals 18 years or older who had an Icelandic electronic ID (total population ≥18 years on January 1, 2020 was 282,770[19]). Recruitment was mainly through social media and public media coverage, though participants in ongoing cohort studies (The SAGA cohort [N = 31,795 women]; iStopMM [N = 80,730 men and women], and Health and well-being of Icelanders [N = 6,102 men and women]) received email or postal invitations to participate. In addition, all individuals in Iceland who tested positive by RT-PCR for SARS-CoV-2 through May 2020 (N = 1,800) received a text message invitation, on June 3, 2020. All participants signed an electronic informed consent and subsequently answered a web-based questionnaire. The study was approved by the National Bioethics Committee (NBC no. 20-073) and the Data Protection Authority.

We performed a cross-sectional analysis of participants responding to the baseline questionnaire from April 24 through July 22, 2020. A total of 22,680 individuals had participated at that time point. We excluded individuals who did not state whether or not they had undergone a diagnostic test (n = 989) or been diagnosed with COVID-19 (n = 19), and those who did not answer all questions on flu-like symptoms (n = 762) or all items in any one of the three measures of depression, anxiety and posttraumatic stress (n = 49) (see supplementary Figure 1). The final analytic sample consisted of 20,861 participants.

For peer review only - http://bmjopen.bmj.com/site/about/guidelines.xhtml

Measures

Sociodemographic and health-related factors

Sociodemographic information included the date of response to the baseline questionnaire, gender, age, education, average monthly income during the past year, relationship status, and residency (capital vs. other regions). We defined relationship status as either being in a relationship (married, cohabiting, or in a relationship yet not cohabiting) or not (single, widowed or divorced). Highest level of education was defined as 1) compulsory; 2) upper secondary/vocational/other; 3) university exam (bachelor's degree or diploma certificate); or 4) master's or doctoral degree. Monthly income categories were defined as 1) low income, <1.653 GBP; 2) low-medium income, 1.653-2.755 GBP; 3) medium income, 2.756-3.858 GBP; 4) medium-high income, 3.859-5.511 GBP; 5) high income, >5.511 GBP (conversion rates according to The Central Bank of Iceland, April 24, 2020[20]).

Health-related information included current smoking status (yes/no), binge drinking during the past two months (defined as ≥4 drinks for women and ≥5 drinks for men or other genders[21]), a previous diagnosis of a psychiatric disorder (made by physicians or psychologists; yes/no), and previous diagnosis of somatic comorbidities including hypertension, diabetes, heart disease, lung disease, chronic kidney disease, cancer, and immunosuppressive state or immunosuppressive therapy (none, one, or two or more comorbid conditions). Additionally, participants reported their height (m) and weight (kg), which was used to estimate body mass index (BMI) as kg/m^2.

Diagnosis of COVID-19 and illness severity

We asked participants if they had been tested for COVID-19 regardless of testing method. If they responded yes, we asked if they had been diagnosed with COVID-19, and if so, how long ago they had been diagnosed, how long they were confined to bed due to COVID-19, and whether they had been hospitalized.

For peer review only - http://bmjopen.bmj.com/site/about/guidelines.xhtml

As the first wave of COVID-19 coincided with the flu season in Iceland, we asked all participants how many days they had experienced nine specific flu-like symptoms over the preceding two months. The symptoms, previously identified as relevant for COVID-19,[22, 23] were: fever, cough, sore throat, impaired sense of taste or smell, headache, myalgia (body aches), gastrointestinal symptoms (abdominal pain, nausea, vomiting, diarrhea), shortness of breath, fatigue and weakness. Response options were: “never”, “1-2 days”, “3-6 days”, “1-2 weeks”, and “more than 2 weeks”, which were scored from “0” (never) to “4” (≥2 weeks). We calculated flu-like symptom burden by summing all symptom scores indicated by each participant (range 0-36 points) and then divided scores into tertiles of the distribution within the COVID-19 cohort (lowest 0-11, mid 12-23, highest 24-36 points).

Symptoms of mental illness

We used the 9-item-Patient Health Questionnaire (PHQ-9) to measure symptoms of depression with the recommended cut-off of ≥10 points serving as a screening indicator for depression in an adult primary care sample.[24] We used the 7-item Generalized Anxiety Disorder scale (GAD-7) to assess symptoms of anxiety. A cut-off of ≥10 was employed as an indicator of moderate to severe symptoms of a generalized anxiety disorder in an adult primary care sample.[25] The 5-item Primary Care PTSD Screen for DSM-5 (PC-PTSD-5) was used to measure symptoms of posttraumatic stress,[26] with a modified version tailored to COVID-19 *(e.g., “Had nightmares about COVID-19?”).* We scored the responses as “0” (never) or “1” (seldom, sometimes, often, very often) with a total score ranging from 0-5. We defined COVID-19-specific PTSD as a PC-PTSD-DSM-5 score of ≥4, which has previously been reported as optimal.[26]

Statistical analysis

First, we contrasted the distribution of sociodemographic and health-related factors between individuals with and without a COVID-19 diagnosis. We then ran univariable and multivariable linear

For peer review only - http://bmjopen.bmj.com/site/about/guidelines.xhtml

regression to detect potential differences in continuous symptoms of depression (PHQ-9), anxiety (GAD-7) and PTSD (PC-PTSD-5) across groups with and without a confirmed COVID-19 diagnosis. We used robust (modified) Poisson regression, with the classical sandwich estimator,[27] to determine the association between a confirmed diagnosis of COVID-19 and risk of depression, anxiety, or PTSD surpassing cut-offs of potential clinical significance. The association is presented as unadjusted and adjusted relative risks (aRRs) with 95% confidence intervals (CIs) and as prevalence differences. All multivariable models included age (continuous), gender (male, female, or other), educational level (classification described above), income (continuous), current smoking (yes/no), previous diagnosis of a psychiatric disorder (yes/no), number of previously diagnosed somatic diseases (0, 1, or ≥2), and the time period of responding to the baseline questionnaire (April 24-30, May 1-7, May 8-23, or May 24-July 22). We then performed these analyses stratified by sex and age (18-39, 40-59, 60 years or older).

We performed several sensitivity analyses. As we were concerned for a potential overlap between physical symptoms of COVID-19 and some items on the mental health assessments (e.g. fatigue), we performed a sensitivity analysis excluding COVID-19 patients diagnosed within 14 days of responding to the questionnaire. We further, reran the analysis using individuals tested but not positive for COVID-19 as a control group. Finally, as our primary Poisson regression analysis included individuals with complete responses to PHQ-9 (n = 18,822), GAD-7 (n = 19,163) and PC-PTSD-5 (n = 17,562), we repeated these analyses, including the entire analytic sample (n = 20,861), using multiple imputation by creating 10 imputed data sets with 10 iterations each.[28]

Limited to individuals diagnosed with COVID-19, we used multivariable Poisson regression to evaluate the association between indicators of COVID-19 severity and mental morbidities. Adjusting for the same covariates as listed above, we explored the association between time confined to bed

For peer review only - http://bmjopen.bmj.com/site/about/guidelines.xhtml

due to COVID-19 (never vs. 1-6 days or 7 days or more), flu-like symptom burden (in tertiles) and whether or not patients had been hospitalized, and mental morbidities among recovering patients.

Next we compared the proportion of individuals with and without COVID-19 (tested and not tested for SARS-CoV-2) who reported each of nine flu-like symptoms for one week or more. We used robust Poisson regression to calculate unadjusted and multivariable-adjusted relative risks of each symptom among individuals tested vs. not tested for SARS-CoV-2, and then diagnosed vs. not diagnosed with COVID-19. The multivariable models included the same covariates as listed above. Using linear regression, we then tested the association between flu-like symptom burden and mental morbidities both among individuals with and without a diagnosis of COVID-19 (tested and not tested for SARS-CoV-2). We then tested whether flu-like symptoms mediated the association between a confirmed COVID-19 diagnosis and mental morbidities by adding flu-like symptoms to the multivariable models. All analyses were conducted in R (Version 3.6.2)

Patient and public involvement

No patients were involved in putting forward the research question or the outcome measures, nor were they involved in developing plans for design or implementation of the study. Dissemination of the results to study participants and the Icelandic population will be obtained through a media outreach (e.g., press release and communication on our study website) on publication of this study.

RESULTS

Background characteristics

Of the 20,861 participants, 5,419 individuals had been tested for SARS-CoV-2, of whom 373 reported having been diagnosed with COVID-19 (97.9% more than two weeks before responding to the questionnaire; Table 1). Compared to other participants, individuals previously diagnosed with COVID-19 were younger (mean age: 48.3 vs. 54.8 years), had a higher educational level and income

For peer review only

and were more likely to binge drink alcohol. They were furthermore less likely to be current smokers and have previously been diagnosed with psychiatric disorders or somatic diseases.

Table 1: Characteristics of the study population, with and without a diagnosis of COVID-19.

	No. (%)		
	**Individuals not diagnosed with COVID-19 (n = 20488)**	**Individuals diagnosed with COVID-19 (n = 373)**	**P-value**
**Gender**			
Male	6133 (29.9)	121 (32.4)	.15
Female	14306 (69.8)	250 (67.0)	
Other	36 (0.2)	2 (0.5)	
Missing	13 (0.1)	0 (0)	
**Age**			
18-29 years	1191 (5.8)	51 (13.7)	<.001
30-39 years	1949 (9.5)	39 (10.5)	
40-49 years	3644 (17.8)	99 (26.5)	
50-59 years	5309 (25.9)	99 (26.5)	
60-69 years	5359 (26.2)	74 (19.8)	
70 years or older	3036 (14.8)	11 (2.9)	
**Highest educational level**			
Compulsory education	2865 (14.0)	30 (8.0)	.008
Upper secondary, vocational or other education	6303 (30.8)	117 (31.4)	
Bachelor's degree or diploma certificate	6517 (31.8)	135 (36.2)	
Master's or Ph.D. degree	4685 (22.9)	91 (24.4)	
Missing	118 (0.6)	0 (0)	
**Average monthly income**[a]			
Low income	3527 (17.2)	50 (13.4)	<.001
Low-medium income	5642 (27.5)	88 (23.6)	
Medium income	4993 (24.4)	82 (22.0)	
Medium-high income	3527 (17.2)	91 (24.4)	
High income	1825 (8.9)	47 (12.6)	
Missing	974 (4.8)	15 (4.0)	
**Residence**			
Capital area	13986 (68.3)	270 (72.4)	.18
Elsewhere in Iceland	6352 (31.0)	102 (27.3)	
Abroad	140 (0.7)	1 (0.3)	

For peer review only - http://bmjopen.bmj.com/site/about/guidelines.xhtml

Missing	10 (0.0)	0 (0)	
**Marital status**			
In a relationship	15735 (76.8)	302 (81.0)	.09
Single	4671 (22.8)	71 (19.0)	
Missing	82 (0.4)	0 (0)	
**BMI category**			
< 25, Normal weight	5931 (28.9)	112 (30.0)	.70
25-30, Overweight	7931 (38.7)	138 (37.0)	
> 30, Obese	6127 (29.9)	118 (31.6)	
Missing	499 (2.4)	5 (1.3)	
**Smoking status**			
No	17873 (87.2)	345 (92.5)	.002
Yes	2521 (12.3)	26 (7.0)	
Missing	94 (0.5)	2 (0.5)	
**Binge drinking**			
No	18255 (89.1)	312 (83.6)	.02
Yes	2233 (10.9)	61 (16.4)	
**Previous diagnosis of psychiatric disorder**			
No	14510 (70.8)	285 (76.4)	.02
Yes	5776 (28.2)	84 (22.5)	
Missing	202 (1.0)	4 (1.1)	
**Physical diseases**			
No comorbidities	11958 (58.4)	263 (70.5)	<.001
One comorbidity	5964 (29.1)	86 (23.1)	
2 or more comorbidities	2453 (12.0)	21 (5.6)	
Missing	113 (0.6)	3 (0.8)	
**Questionnaire answer date**			
April 24-30	7841 (38.3)	102 (27.3)	<.001
May 1-7	5413 (26.4)	76 (20.4)	
May 8-23	4999 (24.4)	53 (14.2)	
May 23-July 22	2235 (10.9)	142 (38.1)	
**Time since diagnosis**			
Less than 2 weeks	-	8 (2.1)	-
2-4 weeks	-	60 (16.1)	
More than 4 weeks	-	305 (81.8)	

[a] Income categories were defined as: Low income, <1.653 GBP; Low-medium income, 1.653-2.755 GBP; Medium income, 2.756-3.858 GBP; Medium-high income, 3.859-5.511 GBP; High income, >5.511 GBP (Conversion rates according to Central Bank of Iceland, April 24, 2020)

For peer review only

COVID-19 and mental morbidities

Individuals diagnosed with COVID-19 reported, compared to others, higher mean scores of depression (6.11 vs. 5.07; *P*<.001) and PTSD (1.90 vs. 1.72; *P*<.001), but not anxiety (4.52 vs. 4.12; *P* = 0.08; see supplementary Table 1). Similarly, multivariable-adjusted relative risks of depression (22.1% vs. 16.2%; aRR 1.48, 95% CI 1.20 to 1.82) and PTSD (19.5% vs. 15.6%; aRR 1.38, 95% CI 1.09 to 1.75) above symptom thresholds were increased among those recovering from COVID-19 compared to others, while not on anxiety (13.1% vs. 11.3%; aRR 1.24, 95% CI 0.93 to 1.64). Risk elevations in mental morbidity in COVID-19 patients was limited to those 40 years or older and was highest among individuals 60 years or older (Table 2). Limiting the analysis to individuals who were diagnosed with COVID-19 more than two weeks before responding to the questionnaire and repeating the analysis using multiple imputation, yielded virtually identical results (see supplementary Table 2 and 3, respectively). Finally, limiting the analysis to individuals who were tested for COVID-19 generated a similar pattern yet slightly lower point estimate (see supplementary Table 4).

For peer review only - http://bmjopen.bmj.com/site/about/guidelines.xhtml

Table 2: The prevalence, crude and multivariable adjusted relative risks and absolute differences in symptoms of depression (PHQ-9), anxiety (GAD-7) and PTSD (PC-PTSD-5) surpassing screening thresholds among individuals with and without a confirmed diagnosis of COVID-19.

	No. (%)					
	Individuals not diagnosed with COVID-19	Individuals diagnosed with COVID-19	cRR (95% CI)	aRR (95% CI)[a]	Absolute difference Crude %	Absolute difference Adjusted[a] %
**Depression**	2992 (16.2)	75 (22.1)	1.36 (1.11 to 1.67)	1.48 (1.20 to 1.82)	5.9	8.4
**Female**	2421 (18.8)	56 (24.8)	1.32 (1.05 to 1.66)	1.43 (1.13 to 1.81)	6.0	8.1
**Male**	559 (10.0)	17 (15.2)	1.52 (0.97 to 2.37)	1.54 (0.96 to 2.47)	5.2	7.2
**18-39 years**	954 (33.3)	23 (27.1)	0.81 (0.57 to 1.16)	0.94 (0.65 to 1.36)	-6.2	-1.8
**40-59 years**	1367 (16.8)	40 (22.3)	1.33 (1.01 to 1.75)	1.72 (1.31 to 2.27)	5.5	14.7
**≥60 years**	671 (8.9)	12 (15.8)	1.77 (1.05 to 2.98)	2.57 (1.52 to 4.36)	6.9	21.8
**Anxiety**	2120 (11.3)	45 (13.1)	1.16 (0.88 to 1.53)	1.24 (0.93 to 1.64)	1.8	2.6
**Female**	1759 (13.4)	34 (14.8)	1.11 (0.81 to 1.51)	1.17 (0.84 to 1.62)	1.4	2.2
**Male**	353 (6.2)	10 (8.9)	1.43 (0.78 to 2.60)	1.39 (0.76 to 2.54)	2.6	3.0
**18-39 years**	770 (26.2)	13 (14.9)	0.57 (0.34 to 0.95)	0.61 (0.36 to 1.04)	-11.3	-9.3
**40-59 years**	951 (11.5)	27 (14.7)	1.28 (0.90 to 1.82)	1.70 (1.20 to 2.41)	3.2	10.5
**≥60 years**	399 (5.2)	5 (6.9)	1.31 (0.56 to 3.06)	2.13 (0.91 to 5.00)	1.7	7.5
**PTSD**	2699 (15.6)	59 (19.5)	1.25 (0.99 to 1.57)	1.38 (1.09 to 1.75)	3.9	7.2
**Female**	2198 (18.6)	46 (22.7)	1.22 (0.94 to 1.58)	1.32 (1.02 to 1.71)	4.1	6.7
**Male**	493 (9.1)	12 (12.1)	1.33 (0.78 to 2.27)	1.60 (0.92 to 2.77)	3.0	7.1
**18-39 years**	597 (24.2)	11 (15.3)	0.63 (0.37 to 1.09)	0.77 (0.45 to 1.32)	-8.9	-6.2
**40-59 years**	1268 (16.7)	31 (19.3)	1.15 (0.84 to 1.59)	1.36 (0.99 to 1.88)	2.6	7.8
**≥60 years**	834 (11.6)	17 (24.3)	2.09 (1.38 to 3.18)	2.67 (1.78 to 4.01)	12.7	27.9

[a] Adjusted for age (continuous variable), gender (male, female, or other), educational level (compulsory education, high school/trade school/other education, bachelor's degree/diploma certificate, or master's/Ph.D. degree), income (continuous variable), current smoking (yes or no), previous diagnosis of a psychiatric disorder (yes or no), number of previously diagnosed somatic diseases (0, 1, or ≥ 2), and timing of responding to the baseline questionnaire (April 24-30, May 1-7, May 8-23, or May 24-July 22).

For peer review only - http://bmjopen.bmj.com/site/about/guidelines.xhtml

COVID-19 illness severity and mental morbidities

In Table 3, we show the prevalence and adjusted relative risks of mental morbidities among individuals with a diagnosis of COVID-19 by number of days confined to bed, hospitalization and severity of COVID-19 symptoms. We observed dose-dependent associations between these indices of COVID-19 severity and risk of mental morbidities. Risk elevations by days confined to bed and hospitalization due to COVID-19 were statistically significant for depression and anxiety, while flu-like symptom burden was associated with all measures of psychological morbidity.

Table 3: Prevalence and adjusted relative risks (95% confidence intervals) of mental morbidities among recently recovered patients from COVID-19 by disease and symptom severity.

		Depression		Anxiety		PTSD	
	n	% ≥10 PHQ-9	aRR [a] (95%CI)	% ≥10 GAD-7	aRR [a] (95%CI)	% ≥4 PC-PTSD-5	aRR [a] (95%CI)
**Confined to bed due to COVID-19**							
Never	136	10.9	Ref.	9.9	Ref.	15.6	Ref.
1-6 days	131	24.2	2.12 (1.11 to 4.02)	11.3	1.13 (0.55 to 2.32)	18.2	0.94 (0.53 to 1.69)
7 days or more	105	33.3	3.67 (1.97 to 6.86)	19.4	2.58 (1.29 to 5.15)	26.2	1.65 (0.92 to 2.96)
**Hospitalized for COVID-19**							
No	341	19.9	Ref.	12.7	Ref.	18.3	Ref.
Yes	32	48.1	2.72 (1.67 to 4.44)	17.2	1.74 (0.85 to 3.57)	32.0	2.05 (0.97 to 4.32)
**COVID-19 symptom severity**							
Lowest tertile	115	5.8	Ref.	4.9	Ref.	9.6	Ref.
Mid tertile	161	18.8	2.70 (1.16 to 6.28)	12.1	2.15 (0.81 to 5.70)	20.1	1.76 (0.88 to 3.55)
Highest tertile	97	47.1	6.42 (2.77 to 14.87)	23.9	3.91 (1.45 to 10.51)	30.7	2.70 (1.30 to 5.59)

[a] Adjusted for age (continuous variable), gender (male, female, or other), educational level (compulsory education, high school/trade school/other education, bachelor's degree/diploma certificate, or master's/Ph.D. degree), income (continuous variable), current smoking (yes or no), previous diagnosis of a psychiatric disorder (yes or no), number of previously diagnosed somatic diseases (0, 1, or ≥ 2), and timing of responding to the baseline questionnaire (April 24-30, May 1-7, May 8-23, or May 24-July 22).

For peer review only - http://bmjopen.bmj.com/site/about/guidelines.xhtml

Flu-like symptoms and mental morbidities

In Figure 1, we present the proportions of individuals with flu-like symptoms lasting at least one week during the preceding two months among recovering COVID-19 patients, those who tested negative and those never tested for SARS-CoV-2 (more detailed in supplementary Table 5). Individuals who tested negative reported 29-91% higher prevalence of symptoms than those never tested for SARS-CoV-2 (see supplementary Table 6), while individuals with a confirmed COVID-19 diagnosis had, compared to all others, dramatically increased adjusted risk ratios of all symptoms: 3.49 for gastrointestinal symptoms, 3.66 for myalgia, 4.16 for fatigue, 4.58 for sore throat, 4.64 for headache, 9.26 for shortness of breath, 19.10 for fever, and 32.52 for impaired sense of taste or smell (see supplementary Table 7).

We found that flu-like symptom burden was positively associated with symptom levels of depression, anxiety and PTSD, regardless of COVID-19 diagnosis (Figure 2 and supplementary Table 8). A stepwise increase across tertiles in flu-like symptom burden was associated with a rise in mean levels of depression, anxiety and PTSD among patients recovering from COVID-19, individuals who tested negative and those never tested for SARS-CoV-2. Recovering COVID-19 patients reporting low to medium flu-like symptom burden (low and mid tertiles) presented with lower levels of depression and anxiety compared to those with same levels of flu-like symptom burden but without a diagnosis of COVID-19 (Figure 2 and supplementary Table 8).

Finally, when flu-like symptom burden was added to the multivariable models (presented in Table 2), the direction of the adjusted relative risks of depression (0.63, 95% CI 0.51 to 0.77) and PTSD (0.73, 95% CI, 0.57 to 0.93) reversed in patients recovering from COVID-19 compared to others, indicating that flu-like symptoms mediated the risks of mental morbidity in this otherwise pre-pandemic healthy population.

For peer review only - http://bmjopen.bmj.com/site/about/guidelines.xhtml

DISCUSSION

The findings of this study suggest that patients recovering from COVID-19 may experience elevated risks of depression and PTSD, particularly if recovering from a severe disease. We found that mental morbidities among recovering COVID-19 patients were strongly associated with older age, greater flu-like symptom burden, extended time confined to bed and hospitalization due to COVID-19.

To our knowledge, this is the first study addressing mental morbidities among recovering COVID-19 patients in a general population setting, including outpatients. Our findings are in line with the vast literature reporting high rates of mental morbidities among current or discharged COVID-19 inpatients.[9, 10, 12, 13] Several mechanisms may play a role in the increased risk of mental health morbidities among patients recovering from a severe COVID-19 illness. These include worries and fear of infecting others, shame or stigma associated with being infected with COVID-19,[29] and uncertainty of the prognosis.[30] Indeed, the diagnosis of potentially life-threatening diseases, e.g. cancer, has previously been associated with a dramatic rise in risks of psychiatric disorders[31] and suicide.[32] In addition, dysregulated immune response, e.g. the documented cytokine storm associated with COVID-19,[33] may independently affect risks of psychiatric disorders in COVID-19 patients,[34] as has been documented in patients with other infections.[35]

Our mediation analysis suggests that flu-like symptom burden mediated the risk elevations in psychiatric morbidities among patients recovering from COVID-19. Moreover, flu-like symptoms among individuals without a confirmed COVID-19 diagnosis were also strongly associated with symptoms of mental morbidities, suggesting that the association is not limited to COVID-19. Indeed, COVID-19 patients reporting low-medium flu-like symptom burden, had lower levels of depression and anxiety than individuals without a diagnosis of COVID-19 but with similar levels of flu-like symptoms. While the healthy pre-pandemic profile of the COVID-19 population may be an indicator of psychological resilience during recovery from COVID-19, it is also possible that the systematic,

For peer review only - http://bmjopen.bmj.com/site/about/guidelines.xhtml

daily surveillance calls to all COVID-19 patients from the specialized COVID-19 outpatient clinic at the National University Hospital had a positive mental health impact on patients.

This population-based study includes more than 20% of all patients diagnosed with COVID-19 by RT-PCR during the first wave of the epidemic in Iceland. The diagnostic rate in Iceland was among the highest worldwide during the first wave of the epidemic,[36, 37] allowing us to perform sensitivity analyses limited to individuals who all had been screened for SARS-CoV-2. The extensive and well-piloted questionnaire allowed us to obtain validated symptom assessment of mental morbidities along with detailed information on flu-like symptom burden for all individuals during the flu season in Iceland, together with a wide range of potential confounders in the association between COVID-19 and mental morbidities.

However, our study is cross-sectional and therefore less informative on the directionality of the studied associations. Indeed, a newly published study using data from the UK Biobank suggests that individuals with a history of pre-pandemic psychiatric disorders are more likely to be hospitalized and die from COVID-19.[38] Thus, it is possible that patients who were already experiencing psychological symptoms suffered the most severe COVID-19-related symptomology yielding the observed associations. However, we included previous diagnoses of psychiatric disorders in all our models which should, to some extent, alleviate this concern. Nevertheless, it is possible that individuals with severe symptoms of depression and anxiety were more likely to report high levels of flu-like symptoms during the preceding two months when responding to the questionnaire, yielding a detrimental outcome-dependent misclassification. Indeed, the instrument to assess flu-like symptom burden included similar items (e.g. fatigue) as are included in the PHQ-9 instrument which, to some extent, could explain the association between COVID-19 and depression.

For peer review only - http://bmjopen.bmj.com/site/about/guidelines.xhtml

Yet, more objective assessments of illness severity, e.g. the number of days confined to bed or hospitalized due to COVID-19 -- both of which were strongly associated with mental morbidities among patients -- might to a lesser extent be influenced by the participants' mental status.

Participants recovering from COVID-19 may differ in terms of their mental health status from COVID-19 patients who did not participate in our study. While we were not able to disentangle the direction of such selection, our observed prevalence of depression and anxiety among recovering COVID-19 patients resembles prevalence rates recently reported in a meta-analysis of diverse COVID-19 patient populations elsewhere.[9] Finally, our study is nested within a small, economically and socially secure society with virtually free and high-quality health care for all, thus the findings may not be generalizable to other settings.

In conclusion, the relatively healthy first-wave COVID-19 population in Iceland presented with increased risk of depression and PTSD in the early weeks of recovery from their illness, particularly those recovering from a severe disease. These findings motivate further follow-up studies of mental health among patients recovering from COVID-19 and other serious infections, and heightened clinical surveillance of those recovering from a severe illness.

For peer review only

Contributors: UAV, TA, and JJ had full access to all of the data in the study and take responsibility for the integrity of the data and the accuracy of the data analysis. UAV, AH, KSS, IM, JJ, TA conceived and design the study. UAV, AH, KSS, IM, JJ, TA analysed the data, and all authors interpreted the data. UAV, KSS, HYH, IM drafted the manuscript. All authors provided critical revision of the manuscript for important intellectual content. UAV, AH, HR, GT, TA, JJ, SYK, ÞL, SHE, DGG, ADM provided administrative, technical, or material support. The corresponding author attests that all listed authors meet authorship criteria and that no others meeting the criteria have been omitted.

Funding: This study was funded by the Icelandic government and a grant from NordForsk (Mental morbidity trajectories in COVID-19 across risk populations of five nations, grant 105668, Dr. UAV). The funding sources had no role in the design and implementation of the study; in data collection, management, analysis, and interpretation of the data; preparation, review, or approval of the manuscript; and decision to submit the manuscript for publication.

Competing interests: All authors complete the ICMJE uniform disclosure form at www.icmje.org/coi_disclosure.pdf and declare no support from any organisation for the submitted work (except those described above); no financial relationships with any organisations that might have an interest in the submitted work in the previous three years; no other relationships or activities that could appear to have influenced the submitted work.

Ethical approval: The study was approved by the National Bioethics Committee of Iceland, Reykjavik (NBC no. 20-073) and the Data Protection Authority.

Data sharing: The original data and R codes are available on request upon approval of the National Bioethics Committee and the Data Protection Authority.

For peer review only

The lead author/guarantor of the study (UAV) affirms that this manuscript is an honest, accurate, and transparent account of the study being reported; that no important aspects of the study have been omitted; and that any discrepancies from the study as planned (and, if relevant, registered) have been explained.

The Corresponding Author (UAV) has the right to grant on behalf of all authors and does grant on behalf of all authors, an exclusive licence on a worldwide basis to the BMJ Publishing Group Ltd to permit this article (if accepted) to be published in BMJ editions and any other BMJPGL products and sublicences such use and exploit all subsidiary rights, as set out in our licence.

For peer review only

References

1. World Health Organization [WHO]. WHO Coronavirus Disease (COVID-19) Dashboard. 2020. Available from: https://covid19.who.int/
2. Carfì A, Bernabei R, Landi F. Persistent Symptoms in Patients After Acute COVID-19. *JAMA* 2020;324:603-05. doi: 10.1001/jama.2020.12603
3. Carvalho-Schneider C, Laurent E, Lemaignen A, et al. Follow-up of adults with non-critical COVID-19 two months after symptoms' onset. *Clin Microbiol Infect* 2020. doi: 10.1016/j.cmi.2020.09.052
4. Halpin SJ, McIvor C, Whyatt G, et al. Postdischarge symptoms and rehabilitation needs in survivors of COVID-19 infection: A cross-sectional evaluation. *J Med Virol* 2020. doi: 10.1002/jmv.26368
5. Tolba M, Abo Omirah M, Hussein A, et al. Assessment and characterisation of post-COVID-19 manifestations. *Int J Clin Pract* 2020:e13746. doi: 10.1111/ijcp.13746
6. Xiong Q, Xu M, Li J, et al. Clinical sequelae of COVID-19 survivors in Wuhan, China: a single-centre longitudinal study. *Clin Microbiol Infect* 2020. doi: 10.1016/j.cmi.2020.09.023
7. Moldofsky H, Patcai J. Chronic widespread musculoskeletal pain, fatigue, depression and disordered sleep in chronic post-SARS syndrome; a case-controlled study. *BMC Neurol* 2011;11:37. doi: 10.1186/1471-2377-11-37
8. Ahmed H, Patel K, Greenwood DC, et al. Long-term clinical outcomes in survivors of severe acute respiratory syndrome and Middle East respiratory syndrome coronavirus outbreaks after hospitalisation or ICU admission: A systematic review and meta-analysis. *J Rehabil Med* 2020;52:jrm00063. doi: 10.2340/16501977-2694
9. Deng J, Zhou F, Hou W, et al. The prevalence of depression, anxiety, and sleep disturbances in COVID-19 patients: a meta-analysis. *Ann N Y Acad Sci* 2020. doi: 10.1111/nyas.14506
10. Bo HX, Li W, Yang Y, et al. Posttraumatic stress symptoms and attitude toward crisis mental health services among clinically stable patients with COVID-19 in China. *Psychol Med* 2020:1-2. doi: 10.1017/s0033291720000999
11. Krishnamoorthy Y, Nagarajan R, Saya GK, et al. Prevalence of psychological morbidities among general population, healthcare workers and COVID-19 patients amidst the COVID-19 pandemic: A systematic review and meta-analysis. *Psychiatry Res* 2020;293:113382. doi: 10.1016/j.psychres.2020.113382
12. Mazza MG, De Lorenzo R, Conte C, et al. Anxiety and depression in COVID-19 survivors: Role of inflammatory and clinical predictors. *Brain Behav Immun* 2020;89:594-600. doi: 10.1016/j.bbi.2020.07.037
13. Taquet M, Luciano S, Geddes JR, et al. Bidirectional associations between COVID-19 and psychiatric disorder: retrospective cohort studies of 62 354 COVID-19 cases in the USA. *Lancet Psychiatry* 2020. doi: 10.1016/s2215-0366(20)30462-4
14. Wang C, Pan R, Wan X, et al. Immediate Psychological Responses and Associated Factors during the Initial Stage of the 2019 Coronavirus Disease (COVID-19) Epidemic among the General Population in China. *Int J Environ Res Public Health* 2020;17:1729. doi: 10.3390/ijerph17051729
15. Wang C, Pan R, Wan X, et al. A longitudinal study on the mental health of general population during the COVID-19 epidemic in China. *Brain Behav Immun* 2020;87:40-48. doi: 10.1016/j.bbi.2020.04.028
16. Helgason D, Eythorsson E, Olafsdottir LB, et al. Beating the odds with systematic individualized care: Nationwide prospective follow-up of all patients with COVID-19 in Iceland. *J Intern Med* 2020. doi: 10.1111/joim.13135
17. Icelandic Directorate of Health and the Department of Civil Protection and Emergency Management. COVID-19 in Iceland – Statistics 28 Feb - 14 June 2020. 2020. Available from: https://www.covid.is/data-old

For peer review only

For peer review only - http://bmjopen.bmj.com/site/about/guidelines.xhtml

18. COVID-19 National Resilience Cohort. About the study. 2020. Available from: https://lidanicovid.is/about-the-study/
19. Statistics Iceland. Population by sex and age 1841-2020. 2020. Available from: https://px.hagstofa.is/pxen/pxweb/en/Ibuar/Ibuar__mannfjoldi__1_yfirlit__yfirlit_mannfjolda/MAN00101.px/?rxid=f6dc8cfa-1b31-43cb-8f39-cec23335bd55
20. Central Bank of Iceland. Official exchange rate. 2020. Available from: https://www.cb.is/statistics/official-exchange-rate/
21. Centers for Disease Control and Prevention. Binge Drinking. 2019. Available from: https://www.cdc.gov/alcohol/fact-sheets/binge-drinking.htm
22. Chen N, Zhou M, Dong X, et al. Epidemiological and clinical characteristics of 99 cases of 2019 novel coronavirus pneumonia in Wuhan, China: a descriptive study. *Lancet* 2020;395:507-13. doi: 10.1016/s0140-6736(20)30211-7
23. Wang D, Hu B, Hu C, et al. Clinical Characteristics of 138 Hospitalized Patients With 2019 Novel Coronavirus-Infected Pneumonia in Wuhan, China. *JAMA* 2020;323:1061-69. doi: 10.1001/jama.2020.1585
24. Kroenke K, Spitzer RL, Williams JB. The PHQ-9: Validity of a brief depression severity measure. *J Gen Intern Med* 2001;16:606-13. doi: 10.1046/j.1525-1497.2001.016009606.x
25. Spitzer RL, Kroenke K, Williams JBW, et al. A brief measure for assessing generalized anxiety disorder: The GAD-7. *Arch Intern Med* 2006;166:1092-97. doi: 10.1001/archinte.166.10.1092
26. Prins A, Bovin MJ, Smolenski DJ, et al. The Primary Care PTSD Screen for DSM-5 (PC-PTSD-5): Development and Evaluation Within a Veteran Primary Care Sample. *J Gen Intern Med* 2016;31:1206-11. doi: 10.1007/s11606-016-3703-5
27. Zou G. A modified poisson regression approach to prospective studies with binary data. *Am J Epidemiol* 2004;159:702-6. doi: 10.1093/aje/kwh090
28. van Buuren S, Groothuis-Oudshoorn K. mice: Multivariate Imputation by Chained Equations in R. *J Stat Softw* 2011;45:1-67.
29. Liu D, Baumeister RF, Veilleux JC, et al. Risk factors associated with mental illness in hospital discharged patients infected with COVID-19 in Wuhan, China. *Psychiatry Res* 2020;292:113297. doi: 10.1016/j.psychres.2020.113297
30. Sahoo S, Mehra A, Dua D, et al. Psychological experience of patients admitted with SARS-CoV-2 infection. *Asian J Psychiatr* 2020;54:102355. doi: 10.1016/j.ajp.2020.102355
31. Lu D, Andersson TM, Fall K, et al. Clinical Diagnosis of Mental Disorders Immediately Before and After Cancer Diagnosis: A Nationwide Matched Cohort Study in Sweden. *JAMA Oncol* 2016;2:1188-96. doi: 10.1001/jamaoncol.2016.0483
32. Fang F, Fall K, Mittleman MA, et al. Suicide and cardiovascular death after a cancer diagnosis. *N Engl J Med* 2012;366:1310-8. doi: 10.1056/NEJMoa1110307
33. Huang C, Wang Y, Li X, et al. Clinical features of patients infected with 2019 novel coronavirus in Wuhan, China. *Lancet* 2020;395:497-506. doi: 10.1016/s0140-6736(20)30183-5
34. Dantzer R, O'Connor JC, Freund GG, et al. From inflammation to sickness and depression: when the immune system subjugates the brain. *Nat Rev Neurosci* 2008;9:46-56. doi: 10.1038/nrn2297
35. Raison CL, Capuron L, Miller AH. Cytokines sing the blues: inflammation and the pathogenesis of depression. *Trends Immunol* 2006;27:24-31. doi: 10.1016/j.it.2005.11.006
36. Hasell J O-OE, Mathieu E, Ritchie H, Beltekian D, Roser M. To understand the global pandemic, we need global testing – the Our World in Data COVID-19 Testing dataset. Our World Data; March 31, 2020. Available from: https://ourworldindata.org/covid-testing
37. Gudbjartsson DF, Norddahl GL, Melsted P, et al. Humoral Immune Response to SARS-CoV-2 in Iceland. *N Engl J Med* 2020;383:1724-34. doi: 10.1056/NEJMoa2026116
38. Yang H, Chen W, Hu Y, et al. Pre-pandemic psychiatric disorders and risk of COVID-19: a UK Biobank cohort analysis. *Lancet Healthy Longevity* 2020;1:e69-e79. doi: 10.1016/S2666-7568(20)30013-1

For peer review only - http://bmjopen.bmj.com/site/about/guidelines.xhtml

Figure legends

Figure 1: The proportion of individuals with and without (tested and not tested) a confirmed diagnosis of COVID-19 with flu-like symptoms of one week or more during the last two months before responding to the baseline questionnaire.

Figure 2: Adjusted symptom scores of depression, anxiety and PTSD by flu-like symptom burden among individuals with a recent COVID-19 diagnosis, those who tested negative and those never tested for SARS-CoV-2.

For peer review only

For peer review only - http://bmjopen.bmj.com/site/about/guidelines.xhtml

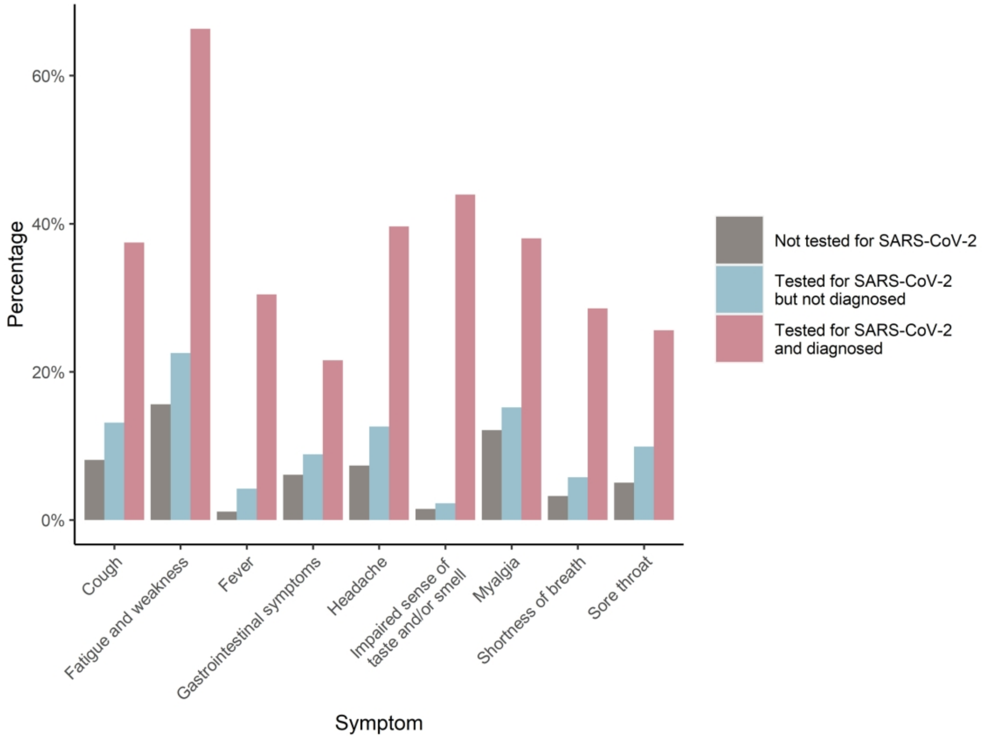

Figure 1 / The proportion of individuals with and without (tested and not tested) a confirmed diagnosis of COVID-19 with flu-like symptoms of one week or more during the last two months before responding to the baseline questionnaire.

203x152mm (300 x 300 DPI)

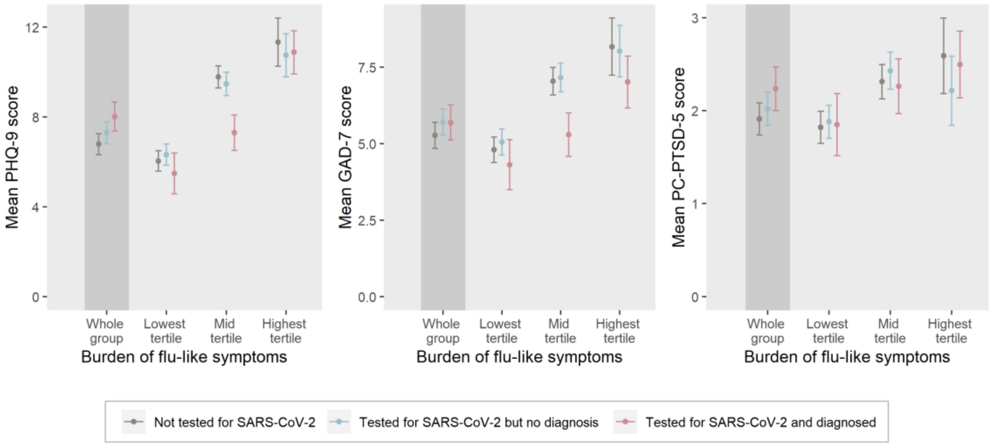

Figure 2 / Adjusted symptom scores of depression, anxiety and PTSD by flu-like symptom burden among individuals with a recent COVID-19 diagnosis, those who tested negative and those never tested for SARS-CoV-2.

254x127mm (300 x 300 DPI)

Supplementary material

Supplementary Figure 1. The analytic sample within the COVID-19 National Resilience Cohort.

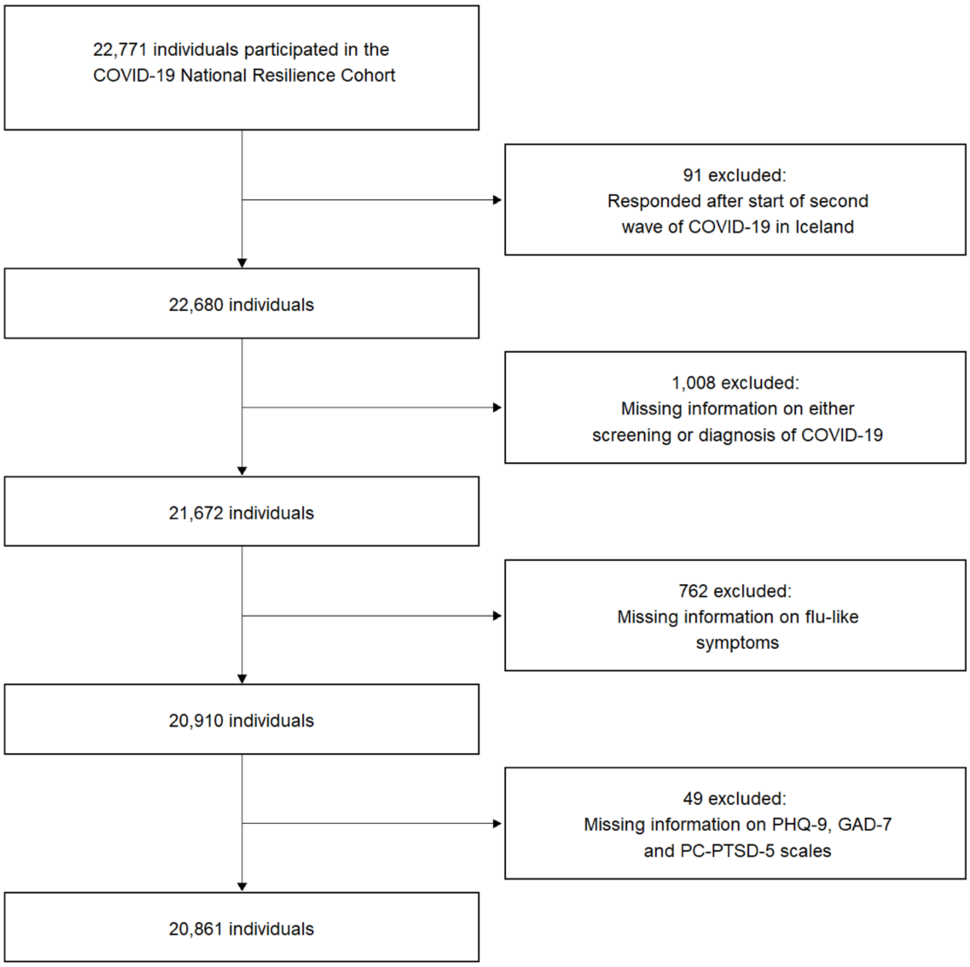

Supplementary Table 1. Mean and median scores on PHQ-9, GAD-7, and PC-PTSD-5 among individuals with and without a recent COVID-19 diagnosis.

	Mean scores		Median scores		P-value	Adj. P-value
	**Individuals not diagnosed with COVID-19**	**Individuals diagnosed with COVID-19**	**Individuals not diagnosed with COVID-19**	**Individuals diagnosed with COVID-19**		
**Depression**	5.07	6.11	4	5	<.001	<.001
**Anxiety**	4.12	4.52	3	4	.07	.08
**PTSD**	1.72	1.90	2	2	.007	<.001

[a] Adjusted for age (continuous variable), gender (male, female, or other), educational level (compulsory education, high school/trade school/other education, bachelor's degree/diploma certificate, or master's/Ph.D. degree), income (continuous variable), current smoking (yes or no), previous diagnosis of a psychiatric disorder (yes or no), number of previously diagnosed somatic diseases (0, 1, or ≥ 2), and timing of responding to the baseline questionnaire (April 24-30, May 1-7, May 8-23, or May 24-July 22).

For peer review only

Supplementary Table 2. The prevalence, crude and multivariable adjusted relative risks and absolute differences in symptoms of depression (PHQ-9), anxiety (GAD-7) and PTSD (PC-PTSD-5) surpassing screening thresholds among individuals with and without a confirmed diagnosis of COVID-19 (*diagnosed at least two weeks before responding)*.

	No. (%)					
	**Individuals not diagnosed with COVID-19**	**Individuals diagnosed with COVID-19**	**cRR (95% CI)**	**aRR (95% CI)[a]**	**Absolute difference Crude %**	**Absolute difference Adjusted[a] %**
**Depression**	2992 (16.2)	73 (22.0)	1.36 (1.11 to 1.67)	1.49 (1.20 to 1.84)	5.8	8.6
**Female**	2421 (18.8)	55 (24.9)	1.32 (1.05 to 1.67)	1.45 (1.14 to 1.83)	6.1	8.4
**Male**	559 (10.0)	16 (14.7)	1.47 (0.93 to 2.33)	1.52 (0.93 to 2.49)	4.7	6.9
**18-39 years**	954 (33.3)	22 (26.8)	0.81 (0.56 to 1.16)	0.95 (0.65 to 1.39)	-6.5	-1.5
**40-59 years**	1367 (16.8)	39 (22.3)	1.32 (1.00 to 1.75)	1.72 (1.30 to 2.28)	5.5	14.7
**≥60 years**	671 (8.9)	12 (16.0)	1.79 (1.06 to 3.02)	2.59 (1.53 to 4.39)	7.1	22.0
**Anxiety**	2120 (11.3)	45 (13.1)	1.16 (0.88 to 1.54)	1.25 (0.94 to 1.67)	1.8	2.9
**Female**	1759 (13.4)	33 (14.7)	1.10 (0.80 to 1.51)	1.17 (0.84 to 1.64)	1.3	2.3
**Male**	353 (6.2)	10 (9.1)	1.46 (0.80 to 2.67)	1.46 (0.80 to 2.64)	2.9	3.5
**18-39 years**	770 (26.2)	13 (15.5)	0.59 (0.36 to 0.98)	0.64 (0.38 to 1.09)	-10.7	-8.6
**40-59 years**	951 (11.5)	26 (14.4)	1.26 (0.88 to 1.80)	1.68 (1.18 to 2.41)	2.9	10.2
**≥60 years**	399 (5.2)	5 (6.9)	1.32 (0.57 to 3.10)	2.15 (0.91 to 5.03)	1.7	7.5
**PTSD**	2699 (15.6)	56 (18.9)	1.21 (0.95 to 1.54)	1.35 (1.06 to 1.73)	3.3	6.7
**Female**	2198 (18.6)	43 (21.7)	1.17 (0.89 to 1.53)	1.27 (0.97 to 1.67)	3.1	5.7
**Male**	493 (9.1)	12 (12.4)	1.36 (0.79 to 2.32)	1.64 (0.95 to 2.83)	3.3	7.5
**18-39 years**	597 (24.2)	10 (14.3)	0.59 (0.33 to 1.05)	0.72 (0.41 to 1.28)	-9.9	-7.4
**40-59 years**	1268 (16.7)	29 (18.5)	1.11 (0.79 to 1.54)	1.31 (0.94 to 1.84)	1.8	6.7
**≥60 years**	834 (11.6)	17 (24.6)	2.12 (1.40 to 3.23)	2.70 (1.80 to 4.05)	13.0	28.3

[a] Adjusted for age (continuous variable), gender (male, female, or other), educational level (compulsory education, high school/trade school/other education, bachelor's degree/diploma certificate, or master's/Ph.D. degree), income (continuous variable), current smoking (yes or no), previous diagnosis of a psychiatric disorder (yes or no), number of previously diagnosed somatic diseases (0, 1, or ≥ 2), and timing of responding to the baseline questionnaire (April 24-30, May 1-7, May 8-23, or May 24-July 22).

For peer review only - http://bmjopen.bmj.com/site/about/guidelines.xhtml

Supplementary Table 3. The prevalence, crude and multivariable adjusted relative risks and absolute differences in symptoms of depression (PHQ-9), anxiety (GAD-7) and PTSD (PC-PTSD-5) surpassing screening thresholds among individuals with and without a confirmed diagnosis of COVID-19 (*using multiple imputation*).

	No. (%)					
	Individuals not diagnosed with COVID-19	Individuals diagnosed with COVID-19	cRR (95% CI)	aRR (95% CI)[a]	Absolute difference Crude %	Absolute difference Adjusted[a] %
**Depression**	3487 (17.0)	83 (22.3)	1.31 (1.08 to 1.59)	1.44 (1.18 to 1.75)	5.3	8.1
**Female**	2819 (19.7)	64 (25.6)	1.29 (1.05 to 1.61)	1.43 (1.14 to 1.78)	5.9	8.3
**Male**	655 (10.7)	17 (14.1)	1.32 (0.85 to 2.08)	1.35 (0.85 to 2.16)	3.4	4.6
**18-39 years**	1105 (35.2)	26 (28.9)	0.82 (0.59 to 1.14)	0.95 (0.67 to 1.33)	-6.3	-1.8
**40-59 years**	1565 (17.5)	445(22.5)	1.29 (0.99 to 1.68)	1.68 (1.29 to 2.18)	5.0	14.8
**≥60 years**	817 (9.7)	12 (14.8)	1.52 (0.90 to 2.58)	2.23 (1.31 to 3.78)	5.1	17.2
**Anxiety**	2328 (11.3)	45 (12.1)	1.06 (0.81 to 1.40)	1.15 (0.87 to 1.53)	0.8	1.8
**Female**	1940 (13.5)	34 (13.6)	1.01 (0.73 to 1.38)	1.08 (0.78 to 1.50)	0.1	1.0
**Male**	378 (6.2)	10 (8.3)	1.34 (0.73 to 2.45)	1.34 (0.74 to 2.43)	2.1	2.6
**18-39 years**	830 (26.5)	13 (14.4)	0.55 (0.33 to 0.91)	0.61 (0.36 to 1.03)	-12.1	-10.0
**40-59 years**	1037 (11.6)	27 (13.6)	1.18 (0.83 to 1.68)	1.57 (1.11 to 2.23)	2.0	9.0
**≥60 years**	461 (5.5)	5 (5.9)	1.08 (0.46 to 2.53)	1.69 (0.72 to 3.97)	0.4	4.6
**PTSD**	2915 (15.8)	62 (19.2)	1.22 (0.97 to 1.53)	1.36 (1.08 to 1.71)	3.4	6.7
**Female**	2375 (18.7)	48 (22.1)	1.18 (0.92 to 1.52)	1.29 (1.00 to 1.66)	3.4	6.1
**Male**	532 (9.2)	13 (12.4)	1.34 (0.80 to 2.25)	1.57 (0.93 to 2.67)	3.2	6.9
**18-39 years**	642 (24.5)	11 (14.9)	0.61 (0.35 to 1.05)	0.71 (0.41 to 1.22)	-9.6	-7.9
**40-59 years**	1360 (16.8)	33 (19.3)	1.15 (0.84 to 1.57)	1.37 (1.00 to 1.87)	2.5	7.9
**≥60 years**	913 (11.8)	18 (23.1)	1.96 (1.30 to 2.96)	2.47 (1.65 to 3.69)	11.3	23.9

[a] Adjusted for age (continuous variable), gender (male, female, or other), educational level (compulsory education, high school/trade school/other education, bachelor's degree/diploma certificate, or master's/Ph.D. degree), income (continuous variable), current smoking (yes or no), previous diagnosis of a psychiatric disorder (yes or no), number of previously diagnosed somatic diseases (0, 1, or ≥ 2), and timing of responding to the baseline questionnaire (April 24-30, May 1-7, May 8-23, or May 24-July 22).

For peer review only - http://bmjopen.bmj.com/site/about/guidelines.xhtml

Supplementary Table 4. The prevalence, crude and multivariable adjusted relative risks and absolute differences in symptoms of depression (PHQ-9), anxiety (GAD-7) and PTSD (PC-PTSD-5) surpassing screening thresholds among individuals with and without a confirmed diagnosis of COVID-19 (*analysis limited to individuals who were tested for COVID-19*).

	No. (%)					
	Individuals that have been tested for COVID-19 but not diagnosed	Individuals that have been tested for COVID-19 and diagnosed	cRR (95% CI)	aRR (95% CI)[a]	Absolute difference Crude %	Absolute difference Adjusted[a] %
**Depression**	885 (19.4)	75 (22.1)	1.14 (0.92 to 1.40)	1.31 (1.06 to 1.62)	2.7	6.1
**Female**	712 (21.7)	56 (24.8)	1.14 (0.90 to 1.45)	1.30 (1.02 to 1.65)	3.1	6.2
**Male**	168 (13.2)	17 (15.2)	1.15 (0.72 to 1.82)	1.22 (0.75 to 1.97)	2.0	3.7
**18-39 years**	275 (37.6)	23 (27.1)	0.72 (0.50 to 1.03)	0.85 (0.58 to 1.23)	-10.5	-5.2
**40-59 years**	413 (17.8)	40 (22.3)	1.26 (0.94 to 1.68)	1.64 (1.23 to 2.17)	4.5	13.6
**≥60 years**	197 (13.1)	12 (15.8)	1.21 (0.71 to 2.07)	1.86 (1.09 to 3.18)	2.7	16.4
**Anxiety**	640 (13.8)	45 (13.1)	0.95 (0.72 to 1.26)	1.08 (0.81 to 1.44)	-0.7	1.1
**Female**	531 (15.9)	34 (14.8)	0.94 (0.68 to 1.29)	1.04 (0.75 to 1.45)	-1.1	0.6
**Male**	106 (8.2)	10 (8.9)	1.08 (0.58 to 2.01)	1.11 (0.60 to 2.04)	0.7	1.0
**18-39 years**	237 (31.3)	13 (14.9)	0.48 (0.29 to 0.80)	0.52 (0.31 to 0.89)	-16.4	-13.4
**40-59 years**	286 (12.1)	27 (14.7)	1.21 (0.84 to 1.75)	1.62 (1.13 to 2.31)	2.6	9.7
**≥60 years**	117 (7.7)	5 (6.9)	0.90 (0.38 to 2.12)	1.56 (0.66 to 3.70)	-0.8	5.1
**PTSD**	747 (17.8)	59 (19.5)	1.09 (0.86 to 1.39)	1.26 (0.99 to 1.60)	1.7	5.4
**Female**	626 (21.1)	46 (22.7)	1.08 (0.83 to 1.40)	1.19 (0.91 to 1.56)	1.6	4.5
**Male**	119 (9.7)	12 (12.1)	1.25 (0.71 to 2.17)	1.52 (0.86 to 2.67)	2.4	6.5
**18-39 years**	168 (27.6)	11 (15.3)	0.55 (0.32 to 0.97)	0.68 (0.39 to 1.18)	-12.3	-9.6
**40-59 years**	376 (17.4)	31 (19.3)	1.11 (0.80 to 1.54)	1.31 (0.94 to 1.83)	1.9	6.9
**≥60 years**	203 (14.2)	17 (24.3)	1.70 (1.11 to 2.63)	2.22 (1.46 to 3.37)	10.1	24.6

[a] Adjusted for age (continuous variable), gender (male, female, or other), educational level (compulsory education, high school/trade school/other education, bachelor's degree/diploma certificate, or master's/Ph.D. degree), income (continuous variable), current smoking (yes or no), previous diagnosis of a psychiatric disorder (yes or no), number of previously diagnosed somatic diseases (0, 1, or ≥ 2), and timing of responding to the baseline questionnaire (April 24-30, May 1-7, May 8-23, or May 24-July 22).

Supplementary Table 5. Raw prevalence of flu-like symptoms during the preceding two months among individuals with and without a recent COVID-19 diagnosis.

	No. (%)		
	**Individuals not diagnosed with COVID-19 (n = 20488)**	**Individuals diagnosed with COVID-19 (n = 373)**	**P-value**
**No reported symptoms**			
Yes	3023 (14.8)	12 (3.2)	<.001
No	17465 (85.2)	361 (96.8)	
**Cough**			
Never	13181 (64.3)	122 (32.7)	<.001
1-2 days	3658 (17.9)	57 (15.3)	
3-6 days	1734 (8.5)	55 (14.7)	
1-2 weeks	754 (3.7)	54 (14.5)	
Over 2 weeks	1161 (5.7)	85 (22.8)	
**Fatigue and weakness**			
Never	7535 (36.8)	32 (8.6)	<.001
1-2 days	6044 (29.5)	39 (10.5)	
3-6 days	3350 (16.4)	54 (14.5)	
1-2 weeks	1276 (6.2)	59 (15.8)	
Over 2 weeks	2283 (11.1)	189 (50.7)	
**Fever**			
Never	17362 (84.7)	127 (34.0)	<.001
1-2 days	1907 (9.3)	58 (15.5)	
3-6 days	831 (4.1)	74 (19.8)	
1-2 weeks	227 (1.1)	53 (14.2)	
Over 2 weeks	161 (0.8)	61 (16.4)	
**Gastrointestinal symptoms**			
Never	12589 (61.4)	162 (43.4)	<.001
1-2 days	4565 (22.3)	69 (18.5)	
3-6 days	1941 (9.5)	61 (16.4)	
1-2 weeks	585 (2.9)	42 (11.3)	
Over 2 weeks	808 (3.9)	39 (10.5)	
**Headache**			
Never	8583 (41.9)	73 (19.6)	<.001
1-2 days	6754 (33.0)	57 (15.3)	
3-6 days	3381 (16.5)	95 (25.5)	
1-2 weeks	816 (4.0)	62 (16.6)	
Over 2 weeks	954 (4.7)	86 (23.1)	

For peer review only

	No. (%)		
	Individuals not diagnosed with COVID-19 (n = 20488)	Individuals diagnosed with COVID-19 (n = 373)	P-value
**Impaired sense of taste and/or smell**			
Never	19514 (95.2)	140 (37.5)	<.001
1-2 days	411 (2.0)	25 (6.7)	
3-6 days	220 (1.1)	44 (11.8)	
1-2 weeks	111 (0.5)	55 (14.7)	
Over 2 weeks	232 (1.1)	109 (29.2)	
**Myalgia**			
Never	12253 (59.8)	105 (28.2)	<.001
1-2 days	3512 (17.1)	54 (14.5)	
3-6 days	2072 (10.1)	72 (19.3)	
1-2 weeks	708 (3.5)	81 (21.7)	
Over 2 weeks	1943 (9.5)	61 (16.4)	
**Shortness of breath**			
Never	17111 (83.5)	169 (45.3)	<.001
1-2 days	1809 (8.8)	42 (11.3)	
3-6 days	775 (3.8)	55 (14.7)	
1-2 weeks	312 (1.5)	41 (11.0)	
Over 2 weeks	481 (2.3)	66 (17.7)	
**Sore throat**			
Never	12987 (63.4)	153 (41.0)	<.001
1-2 days	4323 (21.1)	62 (16.6)	
3-6 days	1901 (9.3)	63 (16.9)	
1-2 weeks	632 (3.1)	39 (10.5)	
Over 2 weeks	645 (3.1)	56 (15.0)	

For peer review only

Supplementary Table 6. Prevalence, crude and adjusted relative risks with 95% confidence intervals of flu-like symptoms lasting for 1 week or more, during the preceding two months, among individuals who tested negative for SARS-CoV-2 and those who were never tested.

Symptoms	No. (%) Individuals not tested for SARS-CoV-2 (n = 15442)	No. (%) Individuals tested negative for SARS-CoV-2 (n = 5046)	cRR (95% CI)	aRR (95% CI)[a]
Cough	1249 (8.1)	666 (13.2)	1.60 (1.46 to 1.76)	1.59 (1.45 to 1.75)
Fatigue and weakness	2420 (15.7)	1139 (22.3)	1.45 (1.36 to 1.55)	1.37 (1.29 to 1.46)
Fever	176 (1.1)	212 (4.2)	3.71 (3.02 to 4.56)	3.49 (2.84 to 4.29)
Gastrointestinal symptoms	946 (6.1)	447 (8.9)	1.48 (1.33 to 1.66)	1.37 (1.23 to 1.53)
Headache	1134 (7.3)	636 (12.6)	1.74 (1.58 to 1.91)	1.60 (1.46 to 1.75)
Impaired sense of taste or smell	229 (1.5)	114 (2.3)	1.53 (1.21 to 1.93)	1.64 (1.30 to 2.07)
Myalgia	1881 (12.2)	770 (15.3)	1.26 (1.16 to 1.36)	1.29 (1.19 to 1.39)
Shortness of breath	503 (3.3)	290 (5.7)	1.88 (1.62 to 2.17)	1.84 (1.59 to 2.13)
Sore throat	778 (5.0)	499 (9.9)	1.98 (1.77 to 2.21)	1.91 (1.71 to 2.14)

[a] Adjusted for age (continuous variable), gender (male, female, or other), educational level (compulsory education, high school/trade school/other education, bachelor's degree/diploma certificate, or master's/Ph.D. degree), income (continuous variable), current smoking (yes or no), previous diagnosis of a psychiatric disorder (yes or no), number of previously diagnosed somatic diseases (0, 1, or ≥ 2), and timing of responding to the baseline questionnaire (April 24-30, May 1-7, May 8-23, or May 24-July 22).

For peer review only

Supplementary Table 7. Prevalence, crude and adjusted relative risks with 95% confidence intervals of flu-like symptoms lasting for 1 week or more, during the preceding two months, among individuals with and without a confirmed diagnosis of COVID-19.

Symptoms	No. (%)		cRR (95% CI)	aRR (95% CI)[a]
	Individuals not diagnosed with COVID-19 (n = 20488)	Individuals diagnosed with COVID-19 (n = 373)		
Cough	1915 (9.4)	139 (37.3)	4.15 (3.60 to 4.76)	5.02 (4.35 to 5.81)
Fatigue and weakness	3559 (17.4)	248 (66.5)	3.93 (3.63 to 4.26)	4.16 (3.77 to 4.60)
Fever	388 (1.9)	114 (30.6)	16.77 (13.93 to 20.19)	19.10 (15.49 to 23.48)
Gastrointestinal symptoms	1393 (6.8)	81 (21.7)	3.29 (2.69 to 4.06)	3.49 (2.82 to 4.32)
Headache	1770 (8.6)	148 (39.7)	4.67 (4.10 to 5.37)	4.64 (3.99 to 5.39)
Impaired sense of taste or smell	343 (1.7)	164 (44.0)	27.04 (23.10 to 31.82)	32.52 (27.22 to 38.86)
Myalgia	2651 (12.9)	142 (38.1)	3.03 (2.65 to 3.49)	3.66 (3.16 to 4.23)
Shortness of breath	793 (3.9)	107 (28.7)	7.53 (6.05 to 9.03)	9.26 (7.61 to 11.25)
Sore throat	1277 (6.2)	95 (25.5)	4.17 (3.46 to 5.00)	4.58 (3.78 to 5.54)

[a] Adjusted for age (continuous variable), gender (male, female, or other), educational level (compulsory education, high school/trade school/other education, bachelor's degree/diploma certificate, or master's/Ph.D. degree), income (continuous variable), current smoking (yes or no), previous diagnosis of a psychiatric disorder (yes or no), number of previously diagnosed somatic diseases (0, 1, or ≥ 2), and timing of responding to the baseline questionnaire (April 24-30, May 1-7, May 8-23, or May 24-July 22).

For peer review only

Supplementary Table 8. Adjusted mean symptom scores of depression, anxiety and PTSD by flu-like symptom burden in tertiles among individuals with a recent COVID-19 diagnosis, those who tested negative and those never tested for SARS-CoV-2.

	Multivariable adjusted mean value by tertile of flu-like symptoms[a]				
	**Lowest tertile**	**Mid tertile**	**Highest tertile**	**Beta (95% CI)**	**P-value**
**Depression**					
Patients with COVID-19	5.49	7.30	10.89	0.24 (0.17 to 0.30)	<.001
Tested negative for SARS-CoV-2	6.33	9.48	10.75	0.29 (0.25 to 0.33)	<.001
Not tested for SARS-CoV-2	6.04	9.79	11.34	0.34 (0.33 to 0.36)	<.001
**Anxiety**					
Patients with COVID-19	4.31	5.29	7.02	0.12 (0.06 to 0.17)	<.001
Tested negative for SARS-CoV-2	5.06	7.16	8.02	0.19 (0.16 to 0.23)	.03
Not tested for SARS-CoV-2	4.81	7.04	8.17	0.22 (0.20 to 0.23)	<.001
**PTSD**					
Patients with COVID-19	1.85	2.26	2.50	0.03 (0.01 to 0.05)	<.001
Tested negative for SARS-CoV-2	1.88	2.43	2.21	0.05 (0.04 to 0.06)	.02
Not tested for SARS-CoV-2	1.82	2.31	2.59	0.06 (0.06 to 0.07)	<.001

[a] Adjusted for age (continuous variable), gender (male, female, or other), educational level (compulsory education, high school/trade school/other education, bachelor's degree/diploma certificate, or master's/Ph.D. degree), income (continuous variable), current smoking (yes or no), previous diagnosis of a psychiatric disorder (yes or no), number of previously diagnosed somatic diseases (0, 1, or ≥ 2), and timing of responding to the baseline questionnaire (April 24-30, May 1-7, May 8-23, or May 24-July 22).

For peer review only - http://bmjopen.bmj.com/site/about/guidelines.xhtml

Reporting checklist for cross sectional study.

Based on the STROBE cross sectional guidelines.

Instructions to authors

Complete this checklist by entering the page numbers from your manuscript where readers will find each of the items listed below.

Your article may not currently address all the items on the checklist. Please modify your text to include the missing information. If you are certain that an item does not apply, please write "n/a" and provide a short explanation.

Upload your completed checklist as an extra file when you submit to a journal.

In your methods section, say that you used the STROBE cross sectionalreporting guidelines, and cite them as:

von Elm E, Altman DG, Egger M, Pocock SJ, Gotzsche PC, Vandenbroucke JP. The Strengthening the Reporting of Observational Studies in Epidemiology (STROBE) Statement: guidelines for reporting observational studies.

		Reporting Item	Page Number
**Title and abstract**			
Title	#1a	Indicate the study's design with a commonly used term in the title or the abstract	1
Abstract	#1b	Provide in the abstract an informative and balanced summary of what was done and what was found	3-4
**Introduction**			
Background / rationale	#2	Explain the scientific background and rationale for the investigation being reported	6
Objectives	#3	State specific objectives, including any prespecified hypotheses	6-7
**Methods**			
Study design	#4	Present key elements of study design early in the paper	7

For peer review only

Setting	#5	Describe the setting, locations, and relevant dates, including periods of recruitment, exposure, follow-up, and data collection	7
Eligibility criteria	#6a	Give the eligibility criteria, and the sources and methods of selection of participants.	7
	#7	Clearly define all outcomes, exposures, predictors, potential confounders, and effect modifiers. Give diagnostic criteria, if applicable	8-9
Data sources / measurement	#8	For each variable of interest give sources of data and details of methods of assessment (measurement). Describe comparability of assessment methods if there is more than one group. Give information separately for for exposed and unexposed groups if applicable.	8-9
Bias	#9	Describe any efforts to address potential sources of bias	19-20
Study size	#10	Explain how the study size was arrived at	7
Quantitative variables	#11	Explain how quantitative variables were handled in the analyses. If applicable, describe which groupings were chosen, and why	8-9
Statistical methods	#12a	Describe all statistical methods, including those used to control for confounding	9-11
Statistical methods	#12b	Describe any methods used to examine subgroups and interactions	10-11
Statistical methods	#12c	Explain how missing data were addressed	10
Statistical methods	#12d	If applicable, describe analytical methods taking account of sampling strategy	n/a
Statistical methods	#12e	Describe any sensitivity analyses	10
**Results**			
Participants	#13a	Report numbers of individuals at each stage of study—eg numbers potentially eligible, examined for eligibility, confirmed eligible, included in the study, completing	11-12

For peer review only

		follow-up, and analysed. Give information separately for for exposed and unexposed groups if applicable.	
Participants	#13b	Give reasons for non-participation at each stage	7 (addressed in the methods chapter)
Participants	#13c	Consider use of a flow diagram	Supplementary material: page 1, Supplementary Figure 1
Descriptive data	#14a	Give characteristics of study participants (eg demographic, clinical, social) and information on exposures and potential confounders. Give information separately for exposed and unexposed groups if applicable.	11-13
Descriptive data	#14b	Indicate number of participants with missing data for each variable of interest	7 (addressed in the methods chapter)
Outcome data	#15	Report numbers of outcome events or summary measures. Give information separately for exposed and unexposed groups if applicable.	14-17
Main results	#16a	Give unadjusted estimates and, if applicable, confounder-adjusted estimates and their precision (eg, 95% confidence interval). Make clear which confounders were adjusted for and why they were included	15-16
Main results	#16b	Report category boundaries when continuous variables were categorized	9 (addressed in the methods chapter)
Main results	#16c	If relevant, consider translating estimates of relative risk into absolute risk for a meaningful time period	14-16
Other analyses	#17	Report other analyses done—e.g., analyses of subgroups and interactions, and sensitivity analyses	14
**Discussion**			
Key results	#18	Summarise key results with reference to study objectives	18
Limitations	#19	Discuss limitations of the study, taking into account sources of potential bias or imprecision. Discuss both direction and magnitude of any potential bias.	19-20

For peer review only

Interpretation	#20	Give a cautious overall interpretation considering objectives, limitations, multiplicity of analyses, results from similar studies, and other relevant evidence.	18-20
Generalisability	#21	Discuss the generalisability (external validity) of the study results	20
**Other Information**			
Funding	#22	Give the source of funding and the role of the funders for the present study and, if applicable, for the original study on which the present article is based	21

Notes:

- 13b: 7 (addressed in the methods chapter)
- 13c: Supplementary material: page 1, Supplementary Figure 1
- 14b: 7 (addressed in the methods chapter)
- 16b: 9 (addressed in the methods chapter)

The STROBE checklist is distributed under the terms of the Creative Commons Attribution License CC-BY. This checklist was completed on 15. January 2021 using https://www.goodreports.org/, a tool made by the EQUATOR Network in collaboration with Penelope.ai

For peer review only

BMJ Open

Illness severity and risk of mental morbidities among patients recovering from COVID-19: a cross-sectional study in the Icelandic population

Journal:	*BMJ Open*
Manuscript ID	bmjopen-2021-049967.R1
Article Type:	Original research
Date Submitted by the Author:	29-Jun-2021
Complete List of Authors:	Sævarsdóttir, Karen ; University of Iceland School of Health Sciences, Centre of Public Health Sciences, Faculty of Medicine<br>Hilmarsdóttir, Hildur; University of Iceland School of Health Sciences, Centre of Public Health Sciences, Faculty of Medicine<br>Magnúsdóttir, Ingibjörg; University of Iceland School of Health Sciences, Centre of Public Health Sciences, Faculty of Medicine<br>Hauksdóttir, Arna; University of Iceland School of Health Sciences, Centre of Public Health Sciences, Faculty of Medicine<br>Thordardottir, Edda; University of Iceland School of Health Sciences, Center of Public Health Sciences, Faculty of Medicine<br>Guðjónsdóttir, Ásdís; University of Iceland School of Health Sciences, Center of Public Health Sciences, Faculty of Medicine<br>Tomasson , Gunnar ; University of Iceland School of Health Sciences, Center of Public Health Sciences, Faculty of Medicine; Landspitali<br>Rúnarsdóttir, Harpa; University of Iceland School of Health Sciences, Center of Public Health Sciences, Faculty of Medicine<br>Jónsdóttir, Harpa; University of Iceland School of Health Sciences, Center of Public Health Sciences, Faculty of Medicine; University of Iceland School of Health Sciences, Faculty of Psychology<br>Guðmundsdóttir, Berglind; University of Iceland School of Health Sciences, Faculty of Medicine<br>Pétursdóttir, Guðrún; University of Iceland, Institute for Sustainability Studies<br>Petersen, Pétur; University of Iceland School of Health Sciences, Faculty of Medicine<br>Kristinsson, Sigurður; University of Iceland School of Health Sciences, Faculty of Medicine; Landspitali<br>Love, Thorvardur ; University of Iceland School of Health Sciences, Faculty of Medicine; Landspitali<br>Hansdóttir, Sif; Landspitali<br>Harðardóttir, Hrönn; Landspitali<br>Gudmundsson, Gunnar; University of Iceland School of Health Sciences, Faculty of Medicine; Landspitali<br>Eythorsson, Elias; Landspitali<br>Guðmundsdóttir, Dóra ; The Directorate of Health<br>Sigbjörnsdóttir, Hildur; The Directorate of Health<br>Haraldsdóttir, Sigríður; The Directorate of Health<br>Möller, Alma; The Directorate of Health<br>Palsson, Runolfur; Landspitali; University of Iceland School of Health

	Sciences, Faculty of Medicine<br>Jakobsdóttir, Jóhanna; University of Iceland School of Health Sciences, Center of Public Health Sciences, Faculty of Medicine<br>Aspelund, Thor ; University of Iceland School of Health Sciences, Center of Public Health Sciences, Faculty of Medicine; Icelandic Heart Association<br>Valdimarsdottir, Unnur; University of Iceland School of Health Sciences, Center of Public Health Sciences, Faculty of Medicine; Karolinska Institute, Department of Medical Epidemiology and Biostatistics
**Primary Subject Heading**:	Public health
Secondary Subject Heading:	Epidemiology, Infectious diseases, Mental health
Keywords:	COVID-19, Epidemiology < INFECTIOUS DISEASES, Public health < INFECTIOUS DISEASES, MENTAL HEALTH

SCHOLARONE™
Manuscripts

I, the Submitting Author has the right to grant and does grant on behalf of all authors of the Work (as defined in the below author licence), an exclusive licence and/or a non-exclusive licence for contributions from authors who are: i) UK Crown employees; ii) where BMJ has agreed a CC-BY licence shall apply, and/or iii) in accordance with the terms applicable for US Federal Government officers or employees acting as part of their official duties; on a worldwide, perpetual, irrevocable, royalty-free basis to BMJ Publishing Group Ltd ("BMJ") its licensees and where the relevant Journal is co-owned by BMJ to the co-owners of the Journal, to publish the Work in this journal and any other BMJ products and to exploit all rights, as set out in our licence.

The Submitting Author accepts and understands that any supply made under these terms is made by BMJ to the Submitting Author unless you are acting as an employee on behalf of your employer or a postgraduate student of an affiliated institution which is paying any applicable article publishing charge ("APC") for Open Access articles. Where the Submitting Author wishes to make the Work available on an Open Access basis (and intends to pay the relevant APC), the terms of reuse of such Open Access shall be governed by a Creative Commons licence – details of these licences and which Creative Commons licence will apply to this Work are set out in our licence referred to above.

Other than as permitted in any relevant BMJ Author's Self Archiving Policies, I confirm this Work has not been accepted for publication elsewhere, is not being considered for publication elsewhere and does not duplicate material already published. I confirm all authors consent to publication of this Work and authorise the granting of this licence.

For peer review only

Illness severity and risk of mental morbidities among patients recovering from COVID-19: a cross-sectional study in the Icelandic population

Karen Sól Sævarsdóttir,*[1] medical student, Hildur Ýr Hilmarsdóttir,*[1] psychologist, Ingibjörg Magnúsdóttir,*[1] biostatistician, Arna Hauksdóttir,[1] professor of epidemiology, Edda Bjork Thordardottir,[1] assistant professor of epidemiology, Ásdís Braga Guðjónsdóttir,[1] physician, Gunnar Tómasson,[1,2] consultant in reumatology and assistant professor of medicine, Harpa Rúnarsdóttir,[1] data manager, Harpa Lind Jónsdóttir,[1,9] assistant professor of psychology, Berglind Guðmundsdóttir,[2] professor of psychology, Guðrún Pétursdóttir,[3] associate professor of physiology and director of Saemundur Frodi Center for Sustainability Studies, Pétur Henry Petersen,[2] professor of medicine, Sigurður Yngvi Kristinsson,[2,4] consultant in haematology and professor of medicine, Thorvardur Jon Love,[2,4] consultant of rheumatology and professor of medicine, Sif Hansdóttir,[4] consultant in pulmonology, Hrönn Harðardóttir,[4] consultant in pulmonology, Gunnar Gudmundsson,[2,4] consultant in pulmonology and professor of medicine, Elias Eythorsson,[4] internal medicine trainee, Dóra Guðrún Guðmundsdóttir,[5] director of health promotion, Hildur Sigbjörnsdóttir,[5] data manager, Sigríður Haraldsdóttir,[5] director of health statistics, Alma D. Möller,[5] surgeon general, Runólfur Pálsson,[2,4] professor of medicine and director of internal medicine and emergency services, Jóhanna Jakobsdóttir,*[1] assistant professor of biostatistics, Thor Aspelund,*[1,6] professor of biostatistics, and Unnur Valdimarsdottir,*[1,7,8] professor of epidemiology

*Equal contribution.

[1]Center of Public Health Sciences, Faculty of Medicine, School of Health Sciences, University of Iceland, Reykjavik, Iceland.

[2]Faculty of Medicine, School of Health Sciences, University of Iceland, Reykjavik, Iceland.

[3]Institute of Sustainability Studies, University of Iceland, Reykjavik, Iceland.

[4]Landspitali – The National University Hospital of Iceland, Reykjavik, Iceland.

[5]The Directorate of Health, Reykjavik, Iceland.

[6]The Icelandic Heart Association, Reykjavik, Iceland.

[7]Department of Medical Epidemiology and Biostatistics, Karolinska Institutet, Stockholm, Sweden.

[8]Department of Epidemiology, Harvard TH Chan School of Public Health, Boston, Massachusetts, USA.

[9]Faculty of Psychology, School of Health Sciences, University of Iceland, Reykjavik, Iceland.

Correspondence to:

Prof. Unnur Anna Valdimarsdóttir, unnurav@hi.is, (ORCID 0000-0001-5382-946X)

Center of Public Health Sciences, Faculty of Medicine, School of Health Sciences, University of Iceland, Sturlugata 8, 101 Reykjavik, Iceland.

Word count (manuscript): 3427

For peer review only

Abstract

Objective

To test if patients recovering from COVID-19 are at increased risk of mental morbidities and to what extent such risk is exacerbated by illness severity.

Design

Population-based cross-sectional study.

Setting

Iceland.

Participants

A total of 22,861 individuals were recruited through invitations to existing nationwide cohorts and a social media campaign from April 24 to July 22 2020, of which 373 were patients recovering from COVID-19.

Main outcome measures

Symptoms of depression (Patient Health Questionnaire [PHQ-9]), anxiety (General Anxiety Disorder Scale [GAD-7]), and PTSD (modified Primary Care PTSD Screen for DSM-5 [PC-PTSD-5]) above screening thresholds. Adjusting for multiple covariates and comorbidities, multivariable Poisson regression was used to assess the association between COVID-19 severity and mental morbidities.

Results

Compared to individuals without a diagnosis of COVID-19, patients recovering from COVID-19 had increased risk of depression (22.1% vs. 16.2%; aRR 1.48, 95% CI 1.20 to 1.82) and PTSD (19.5% vs.

For peer review only

15.6%; aRR 1.38, 95% CI 1.09 to 1.75) but not anxiety (13.1% vs. 11.3%; aRR 1.24, 95% CI 0.93 to 1.64). Elevated relative risks were limited to recovering COVID-19 patients 40 years or older and were particularly high among individuals with university education. Among recovering COVID-19 patients, symptoms of depression were particularly common among those in the highest, compared to the lowest tertile of flu-like symptom burden (47.1% vs. 5.8%; aRR 6.42, 95% CI 2.77 to 14.87), among patients confined to bed for seven days or longer compared to those never confined to bed (33.3% vs. 10.9%; aRR 3.67, 95% CI 1.97 to 6.86), and among patients hospitalized for COVID-19 compared to those never admitted to hospital (48.1% vs. 19.9%; aRR 2.72, 95% CI 1.67 to 4.44).

Conclusions

Severe disease course is associated with increased risk of depression and PTSD among patients recovering from COVID-19.

For peer review only

Strengths and limitations of this study

- To our knowledge, this is the first study addressing mental morbidities among recovering COVID-19 patients in a general population setting, including outpatients.
- This study includes more than 20% of all patients diagnosed with COVID-19 by RT-PCR during the first wave of the epidemic in Iceland, where the diagnostic rate was among the highest worldwide, allowing us to perform sensitivity analyses limited to individuals who all had been screened for SARS-CoV-2.
- The extensive and well-piloted questionnaire allowed us to obtain validated symptom assessment of mental morbidities along with detailed information on flu-like symptom burden for all individuals during the flu season in Iceland.
- The cross-sectional design is less informative on the directionality of the studied associations, and as the study is nested within a small, economically and socially secure society, the generalizability of the findings may be limited.
- It is possible that individuals with severe symptoms of depression and anxiety were more likely to report high levels of flu-like symptoms during the preceding two months when responding to the questionnaire, yielding a outcome-dependent misclassification.

For peer review only

For peer review only - http://bmjopen.bmj.com/site/about/guidelines.xhtml

INTRODUCTION

With almost 180 million reported cases and 3.9 million deaths globally,[1] the coronavirus disease 2019 (COVID-19) pandemic remains a major public health challenge worldwide. Beyond the extensive socioeconomic impact and suffering of patients during the illness, there are rising concerns about long-term somatic and psychological impact on recovering patients.[2-6]

Mental health consequences among patients in recent epidemics are well documented.[7, 8] With respect to COVID-19, a meta-analysis of 31 studies suggested a 45-47% pooled prevalence of mild to severe symptoms of depression and anxiety in patients with COVID-19, mostly inpatients.[9] Moreover, extreme rates of posttraumatic stress have been reported among COVID-19 inpatients in China.[10, 11] Previous studies are often small, limited to inpatients, and some lack a control group. Fewer studies have reported symptoms of mental morbidities after recovery from COVID-19 with existing data suggesting high levels of symptoms[12] and elevated rates of diagnosed psychiatric disorders among COVID-19 inpatients during the first weeks and months after hospital discharge.[13-15]

Flu-like symptoms, such as myalgia, cough and sore throat, have been associated with mental morbidities in the general population during the COVID-19 pandemic.[16, 17] To what extent flu-like symptoms are associated with psychological distress in recently recovered COVID-19 patients remains unknown. There are several mechanisms through which COVID-19 may be associated with mental morbidity. Firstly, uncertainty regarding the disease course may give rise to symptoms of anxiety and depression in infected patients.[18] Secondly, serious infection may induce a cytokine storm,[19, 20] leading to exacerbation of the illness and development of psychological symptoms such as depression.[19] The reverse may also be true that psychological symptoms may cause more severe COVID-19 illness through excessive or dysregulated inflammation.[21]

For peer review only

The first wave of COVID-19 in Iceland started on February 28. Through modest but systematic mitigation strategies[22] the incidence of COVID-19 had effectively been attenuated by the end of April 2020, with only sporadic cases occurring until the onset of the second wave of infections on July 22. During this period, more than 20% of the total population was tested using reverse transcription-polymerase chain reaction (RT-PCR); a total of 1820 tested positive for SARS-CoV-2 (0.5% of the total population), of whom 113 were hospitalized (6%), and ten died (0.5%).[23] Here we explore mental health indicators in patients recovering from COVID-19 in the first wave of the pandemic and the potential role of disease severity on the risk of mental morbidities.

METHODS

Study population and design

The COVID-19 National Resilience Cohort was established on April 24, 2020, with the overarching aim of understanding the long-term public health impact of the COVID-19 epidemic in Iceland.[24] Eligible were all Icelandic and English speaking individuals 18 years or older who had an Icelandic electronic ID (total population $\geq$18 years on January 1, 2020 was 282,770[25]). Recruitment was mainly through social media and public media coverage, though participants in ongoing cohort studies (The SAGA cohort [N = 31,795 women]; iStopMM [N = 80,730 men and women], and Health and well-being of Icelanders [N = 6,102 men and women]) received email or postal invitations to participate. In addition, all individuals in Iceland who tested positive by RT-PCR for SARS-CoV-2 through May 2020 (N = 1,800) received a text message invitation, on June 3, 2020. All participants signed an electronic informed consent and subsequently answered a web-based questionnaire. The study was approved by the National Bioethics Committee (NBC no. 20-073) and the Data Protection Authority.

We performed a cross-sectional analysis of participants responding to the baseline questionnaire from April 24 through July 22, 2020. A total of 22,680 individuals had participated at that time point.

For peer review only

We excluded individuals who did not state whether or not they had undergone a diagnostic test (n = 989) or been diagnosed with COVID-19 (n = 19), and those who did not answer all questions on flu-like symptoms (n = 762) or all items in any one of the three measures of depression, anxiety and posttraumatic stress (n = 49) (see supplementary Figure 1). The final analytic sample consisted of 20,861 participants.

Measures

Sociodemographic and health-related factors

Sociodemographic information included the date of response to the baseline questionnaire, gender, age, education, average monthly income during the past year, relationship status, and residency (capital vs. other regions). We defined relationship status as either being in a relationship (married, cohabiting, or in a relationship yet not cohabiting) or not (single, widowed or divorced). Highest level of education was defined as 1) compulsory; 2) upper secondary/vocational/other; 3) university exam (bachelor's degree or diploma certificate); or 4) master's or doctoral degree. Monthly income categories were defined as 1) low income, <1.653 GBP; 2) low-medium income, 1.653-2.755 GBP; 3) medium income, 2.756-3.858 GBP; 4) medium-high income, 3.859-5.511 GBP; 5) high income, >5.511 GBP (conversion rates according to The Central Bank of Iceland, April 24, 2020[26]).

Health-related information included current smoking status (yes/no), binge drinking during the past two months (defined as ≥4 drinks for women and ≥5 drinks for men or other genders[27]), a previous diagnosis of a psychiatric disorder (made by physicians or psychologists; yes/no), and previous diagnosis of somatic comorbidities including hypertension, diabetes, heart disease, lung disease, chronic kidney disease, cancer, and immunosuppressive state or immunosuppressive therapy (none, one, or two or more comorbid conditions). Additionally, participants reported their height (m) and weight (kg), which was used to estimate body mass index (BMI) as kg/m^2.

For peer review only - http://bmjopen.bmj.com/site/about/guidelines.xhtml

Diagnosis of COVID-19 and illness severity

We asked participants if they had been tested for COVID-19 regardless of testing method. If they responded yes, we asked if they had been diagnosed with COVID-19, and if so, how long ago they had been diagnosed, how long they were confined to bed due to COVID-19, and whether they had been hospitalized.

As the first wave of COVID-19 coincided with the flu season in Iceland, we asked all participants how many days they had experienced nine specific flu-like symptoms over the preceding two months. The symptoms, previously identified as relevant for COVID-19,[28, 29] were: fever, cough, sore throat, impaired sense of taste or smell, headache, myalgia (body aches), gastrointestinal symptoms (abdominal pain, nausea, vomiting, diarrhea), shortness of breath, fatigue and weakness. Response options were: “never”, “1-2 days”, “3-6 days”, “1-2 weeks”, and “more than 2 weeks”, which were scored from “0” (never) to “4” (≥2 weeks). We calculated flu-like symptom burden by summing all symptom scores indicated by each participant (range 0-36 points) and then divided scores into tertiles of the distribution within the COVID-19 cohort (lowest 0-11, mid 12-23, highest 24-36 points).

Symptoms of mental illness

We used the 9-item-Patient Health Questionnaire (PHQ-9) to measure symptoms of depression with the recommended cut-off of ≥10 points serving as a screening indicator for depression in an adult primary care sample.[30] We used the 7-item Generalized Anxiety Disorder scale (GAD-7) to assess symptoms of anxiety. A cut-off of ≥10 was employed as an indicator of moderate to severe symptoms of a generalized anxiety disorder in an adult primary care sample.[31] The 5-item Primary Care PTSD Screen for DSM-5 (PC-PTSD-5) was used to measure symptoms of posttraumatic stress,[32] with a modified version tailored to COVID-19 *(e.g., “Had nightmares about COVID-19?”)*. We scored the responses as “0” (never) or “1” (seldom, sometimes, often, very often) with a total

For peer review only

score ranging from 0-5. We defined COVID-19-specific PTSD as a PC-PTSD-DSM-5 score of ≥4, which has previously been reported as optimal.[32]

Statistical analysis

First, we contrasted the distribution of sociodemographic and health-related factors between individuals with and without a COVID-19 diagnosis. We then ran univariable and multivariable linear regression to detect potential differences in continuous symptoms of depression (PHQ-9), anxiety (GAD-7) and PTSD (PC-PTSD-5) across groups with and without a confirmed COVID-19 diagnosis. We used robust (modified) Poisson regression, with the classical sandwich estimator,[33] to determine the association between a confirmed diagnosis of COVID-19 and risk of depression, anxiety, or PTSD surpassing cut-offs of potential clinical significance. The association is presented as unadjusted and adjusted relative risks (aRRs) with 95% confidence intervals (CIs) and as prevalence differences. All multivariable models included age (continuous), gender (male, female, or other), educational level (classification described above), income (continuous), current smoking (yes/no), previous diagnosis of a psychiatric disorder (yes/no), number of previously diagnosed somatic diseases (0, 1, or ≥2), and the time period of responding to the baseline questionnaire (April 24-30, May 1-7, May 8-23, or May 24-July 22). We then performed these analyses stratified by all covariates and performed a likelihood ratio test for effect modification.

We performed several sensitivity analyses. As we were concerned for a potential overlap between physical symptoms of COVID-19 and some items on the mental health assessments (e.g. fatigue), we performed a sensitivity analysis excluding COVID-19 patients diagnosed within 14 days of responding to the questionnaire. We further, reran the analysis using individuals tested but not positive for COVID-19 as a control group. There was a considerable difference between individuals with and without a COVID-19 diagnosis with respect to the date of responding to the questionnaire (Table 1). Therefore, we repeated the multivariable Poisson regression stratified by questionnaire answer

For peer review only - http://bmjopen.bmj.com/site/about/guidelines.xhtml

date, divided into two groups (April 24-May 7, and May 8-July 22). Finally, as our primary Poisson regression analysis included individuals with complete responses to PHQ-9 (n = 18,822), GAD-7 (n = 19,163) and PC-PTSD-5 (n = 17,562), we repeated these analyses, including the entire analytic sample (n = 20,861), using multiple imputation by creating 10 imputed data sets with 10 iterations each.[34]

Limited to individuals diagnosed with COVID-19, we used multivariable Poisson regression to evaluate the association between indicators of COVID-19 severity and mental morbidities. Adjusting for the same covariates as listed above, we explored the association between time confined to bed due to COVID-19 (never vs. 1-6 days or 7 days or more), flu-like symptom burden (in tertiles) and whether or not patients had been hospitalized, and mental morbidities among recovering patients.

Next we compared the proportion of individuals with and without COVID-19 (tested and not tested for SARS-CoV-2) who reported each of nine flu-like symptoms for one week or more. We used robust Poisson regression to calculate unadjusted and multivariable-adjusted relative risks of each symptom among individuals tested vs. not tested for SARS-CoV-2, and then diagnosed vs. not diagnosed with COVID-19. The multivariable models included the same covariates as listed above. Using linear regression, we then tested the association between flu-like symptom burden and mental morbidities both among individuals with and without a diagnosis of COVID-19 (tested and not tested for SARS-CoV-2). We then tested whether flu-like symptoms mediated the association between a confirmed COVID-19 diagnosis and mental morbidities by adding flu-like symptoms to the multivariable models. All analyses were conducted in R (Version 3.6.2)

Patient and public involvement

No patients were involved in putting forward the research question or the outcome measures, nor were they involved in developing plans for design or implementation of the study. Dissemination of

For peer review only - http://bmjopen.bmj.com/site/about/guidelines.xhtml

the results to study participants and the Icelandic population will be obtained through a media outreach (e.g., press release and communication on our study website) on publication of this study.

RESULTS

Background characteristics

Of the 20,861 participants, 5,419 individuals had been tested for SARS-CoV-2, of whom 373 reported having been diagnosed with COVID-19 (97.9% more than two weeks before responding to the questionnaire; Table 1). Compared to other participants, individuals previously diagnosed with COVID-19 were younger (mean age: 48.3 vs. 54.8 years), had a higher educational level and income and were more likely to binge drink alcohol. They were furthermore less likely to be current smokers and have previously been diagnosed with psychiatric disorders or somatic diseases. The analytic sample had similar education[35] and residence distribution as the general population, while the median age was higher and women were overrepresented.[36]

Table 1: Characteristics of the study population, with and without a diagnosis of COVID-19.

	No. (%)		
	**Individuals not diagnosed with COVID-19 (n = 20488)**	**Individuals diagnosed with COVID-19 (n = 373)**	**P-value**
**Gender**			
Male	6133 (29.9)	121 (32.4)	.15
Female	14306 (69.8)	250 (67.0)	
Other	36 (0.2)	2 (0.5)	
Missing	13 (0.1)	0 (0)	
**Age**			
18-29 years	1191 (5.8)	51 (13.7)	<.001
30-39 years	1949 (9.5)	39 (10.5)	
40-49 years	3644 (17.8)	99 (26.5)	
50-59 years	5309 (25.9)	99 (26.5)	
60-69 years	5359 (26.2)	74 (19.8)	
70 years or older	3036 (14.8)	11 (2.9)	

For peer review only - http://bmjopen.bmj.com/site/about/guidelines.xhtml
For peer review only

**Highest educational level**			
Compulsory education	2865 (14.0)	30 (8.0)	.008
Upper secondary, vocational or other education	6303 (30.8)	117 (31.4)	
Bachelor's degree or diploma certificate	6517 (31.8)	135 (36.2)	
Master's or Ph.D. degree	4685 (22.9)	91 (24.4)	
Missing	118 (0.6)	0 (0)	
**Average monthly income[a]**			
Low income	3527 (17.2)	50 (13.4)	<.001
Low-medium income	5642 (27.5)	88 (23.6)	
Medium income	4993 (24.4)	82 (22.0)	
Medium-high income	3527 (17.2)	91 (24.4)	
High income	1825 (8.9)	47 (12.6)	
Missing	974 (4.8)	15 (4.0)	
**Residence**			
Capital area	13986 (68.3)	270 (72.4)	.18
Elsewhere in Iceland	6352 (31.0)	102 (27.3)	
Abroad	140 (0.7)	1 (0.3)	
Missing	10 (0.0)	0 (0)	
**Marital status**			
In a relationship	15735 (76.8)	302 (81.0)	.09
Single	4671 (22.8)	71 (19.0)	
Missing	82 (0.4)	0 (0)	
**BMI category**			
< 25, Normal weight	5931 (28.9)	112 (30.0)	.70
25-30, Overweight	7931 (38.7)	138 (37.0)	
> 30, Obese	6127 (29.9)	118 (31.6)	
Missing	499 (2.4)	5 (1.3)	
**Smoking status**			
No	17873 (87.2)	345 (92.5)	.002
Yes	2521 (12.3)	26 (7.0)	
Missing	94 (0.5)	2 (0.5)	
**Binge drinking**			
No	18255 (89.1)	312 (83.6)	.02
Yes	2233 (10.9)	61 (16.4)	
**Previous diagnosis of psychiatric disorder**			
No	14510 (70.8)	285 (76.4)	.02
Yes	5776 (28.2)	84 (22.5)	
Missing	202 (1.0)	4 (1.1)	
**Physical diseases**			

For peer review only

No comorbidities	11958 (58.4)	263 (70.5)	<.001
One comorbidity	5964 (29.1)	86 (23.1)	
2 or more comorbidities	2453 (12.0)	21 (5.6)	
Missing	113 (0.6)	3 (0.8)	
**Questionnaire answer date**			
April 24-30	7841 (38.3)	102 (27.3)	<.001
May 1-7	5413 (26.4)	76 (20.4)	
May 8-23	4999 (24.4)	53 (14.2)	
May 24-July 22	2235 (10.9)	142 (38.1)	
**Time since diagnosis**			
Less than 2 weeks	-	8 (2.1)	-
2-4 weeks	-	60 (16.1)	
More than 4 weeks	-	305 (81.8)	

[a] Income categories were defined as: Low income, <1.653 GBP; Low-medium income, 1.653-2.755 GBP; Medium income, 2.756-3.858 GBP; Medium-high income, 3.859-5.511 GBP; High income, >5.511 GBP (Conversion rates according to Central Bank of Iceland, April 24, 2020)

COVID-19 and mental morbidities

Individuals diagnosed with COVID-19 reported, compared to others, higher mean scores of depression (6.11 vs. 5.07; *P*<.001) and PTSD (1.90 vs. 1.72; *P*<.001), but not anxiety (4.52 vs. 4.12; *P* = 0.08; see supplementary Table 1). Similarly, multivariable-adjusted relative risks of depression (22.1% vs. 16.2%; aRR 1.48, 95% CI 1.20 to 1.82) and PTSD (19.5% vs. 15.6%; aRR 1.38, 95% CI 1.09 to 1.75) above symptom thresholds were increased among those recovering from COVID-19 compared to others, while not on anxiety (13.1% vs. 11.3%; aRR 1.24, 95% CI 0.93 to 1.64; Table 2). Risk elevations in mental morbidity in COVID-19 patients was limited to those 40 years or older and was highest among individuals 60 years or older (see supplementary Table 2). Individuals with higher educational level were more likely to suffer from symptoms of depression and anxiety after a diagnosis of COVID-19. When stratified by answer date, risk elevations for depression and anxiety in COVID-19 patients were lower for those who answered in the later response period (i.e., May 8-July 22), whereas it was higher for PTSD (see supplementary Table 2) and no statistically significant interactions were observed. Limiting the analysis to individuals who were diagnosed with COVID-19

For peer review only - http://bmjopen.bmj.com/site/about/guidelines.xhtml

more than two weeks before responding to the questionnaire and repeating the analysis using multiple imputation, yielded virtually identical results (see supplementary Table 3 and 4, respectively). Finally, limiting the analysis to individuals who were tested for COVID-19 generated a similar pattern yet slightly lower point estimate (see supplementary Table 5).

Table 2: The prevalence, crude and multivariable adjusted relative risks and absolute differences in symptoms of depression (PHQ-9), anxiety (GAD-7) and PTSD (PC-PTSD-5) surpassing screening thresholds among individuals with and without a confirmed diagnosis of COVID-19.

	No. (%)					
	Individuals not diagnosed with COVID-19	Individuals diagnosed with COVID-19	cRR (95% CI)	aRR (95% CI)[a]	Absolute difference Crude %	Absolute difference Adjusted [a] %
**Depression**	2992 (16.2)	75 (22.1)	1.36 (1.11 to 1.67)	1.48 (1.20 to 1.82)	5.9	8.4
**Anxiety**	2120 (11.3)	45 (13.1)	1.16 (0.88 to 1.53)	1.24 (0.93 to 1.64)	1.8	2.6
**PTSD**	2699 (15.6)	59 (19.5)	1.25 (0.99 to 1.57)	1.38 (1.09 to 1.75)	3.9	7.2

[a] Adjusted for age (continuous variable), gender (male, female, or other), educational level (compulsory education, high school/trade school/other education, bachelor's degree/diploma certificate, or master's/Ph.D. degree), income (continuous variable), current smoking (yes or no), previous diagnosis of a psychiatric disorder (yes or no), number of previously diagnosed somatic diseases (0, 1, or ≥ 2), and timing of responding to the baseline questionnaire (April 24-30, May 1-7, May 8-23, or May 24-July 22).

For peer review only - http://bmjopen.bmj.com/site/about/guidelines.xhtml

COVID-19 illness severity and mental morbidities

In Table 3, we show the prevalence and adjusted relative risks of mental morbidities among individuals with a diagnosis of COVID-19 by number of days confined to bed, hospitalization and severity of COVID-19 symptoms. We observed dose-dependent associations between these indices of COVID-19 severity and risk of mental morbidities. Risk elevations by days confined to bed and hospitalization due to COVID-19 were statistically significant for depression and anxiety, while flu-like symptom burden was associated with all measures of psychological morbidity.

Table 3: Prevalence and adjusted relative risks (95% confidence intervals) of mental morbidities among recently recovered patients from COVID-19 by disease and symptom severity.

		Depression		Anxiety		PTSD	
	n	% ≥10 PHQ-9	aRR [a] (95%CI)	% ≥10 GAD-7	aRR [a] (95%CI)	% ≥4 PC-PTSD-5	aRR [a] (95%CI)
**Confined to bed due to COVID-19**							
Never	136	10.9	Ref.	9.9	Ref.	15.6	Ref.
1-6 days	131	24.2	2.12 (1.11 to 4.02)	11.3	1.13 (0.55 to 2.32)	18.2	0.94 (0.53 to 1.69)
7 days or more	105	33.3	3.67 (1.97 to 6.86)	19.4	2.58 (1.29 to 5.15)	26.2	1.65 (0.92 to 2.96)
**Hospitalized for COVID-19**							
No	341	19.9	Ref.	12.7	Ref.	18.3	Ref.
Yes	32	48.1	2.72 (1.67 to 4.44)	17.2	1.74 (0.85 to 3.57)	32.0	2.05 (0.97 to 4.32)
**COVID-19 symptom severity**							
Lowest tertile	115	5.8	Ref.	4.9	Ref.	9.6	Ref.
Mid tertile	161	18.8	2.70 (1.16 to 6.28)	12.1	2.15 (0.81 to 5.70)	20.1	1.76 (0.88 to 3.55)
Highest tertile	97	47.1	6.42 (2.77 to 14.87)	23.9	3.91 (1.45 to 10.51)	30.7	2.70 (1.30 to 5.59)

[a] Adjusted for age (continuous variable), gender (male, female, or other), educational level (compulsory education, high school/trade school/other education, bachelor's degree/diploma certificate, or master's/Ph.D. degree), income (continuous variable), current smoking (yes or no), previous diagnosis of a psychiatric disorder (yes or no), number of previously diagnosed somatic diseases (0, 1, or ≥ 2), and timing of responding to the baseline questionnaire (April 24-30, May 1-7, May 8-23, or May 24-July 22).

For peer review only - http://bmjopen.bmj.com/site/about/guidelines.xhtml

Flu-like symptoms and mental morbidities

In Figure 1, we present the proportions of individuals with flu-like symptoms lasting at least one week during the preceding two months among recovering COVID-19 patients, those who tested negative and those never tested for SARS-CoV-2 (more detailed in supplementary Table 6). Individuals who tested negative reported 29-91% higher prevalence of symptoms than those never tested for SARS-CoV-2 (see supplementary Table 7), while individuals with a confirmed COVID-19 diagnosis had, compared to all others, dramatically increased adjusted risk ratios of all symptoms: 3.49 for gastrointestinal symptoms, 3.66 for myalgia, 4.16 for fatigue, 4.58 for sore throat, 4.64 for headache, 9.26 for shortness of breath, 19.10 for fever, and 32.52 for impaired sense of taste or smell (see supplementary Table 8).

We found that flu-like symptom burden was positively associated with symptom levels of depression, anxiety and PTSD, regardless of COVID-19 diagnosis (Figure 2 and supplementary Table 9). A stepwise increase across tertiles in flu-like symptom burden was associated with a rise in mean levels of depression, anxiety and PTSD among patients recovering from COVID-19, individuals who tested negative and those never tested for SARS-CoV-2. Recovering COVID-19 patients reporting low to medium flu-like symptom burden (low and mid tertiles) presented with lower levels of depression and anxiety compared to those with same levels of flu-like symptom burden but without a diagnosis of COVID-19 (Figure 2 and supplementary Table 9).

Finally, when flu-like symptom burden was added to the multivariable models (presented in Table 2), the direction of the adjusted relative risks of depression (0.63, 95% CI 0.51 to 0.77) and PTSD (0.73, 95% CI, 0.57 to 0.93) reversed in patients recovering from COVID-19 compared to others, indicating that flu-like symptoms mediated the risks of mental morbidity in this otherwise pre-pandemic healthy population.

For peer review only - http://bmjopen.bmj.com/site/about/guidelines.xhtml

DISCUSSION

The findings of this study suggest that patients recovering from COVID-19 may experience elevated risks of depression and PTSD, particularly if recovering from a severe disease. We found that mental morbidities among recovering COVID-19 patients were strongly associated with older age, higher educational level, greater flu-like symptom burden, extended time confined to bed and hospitalization due to COVID-19.

To our knowledge, this is the first study addressing mental morbidities among recovering COVID-19 patients in a general population setting, including outpatients. Our findings are in line with the vast literature reporting high rates of mental morbidities among current or discharged COVID-19 inpatients.[9, 10, 12, 13] Several mechanisms may play a role in the increased risk of mental health morbidities among patients recovering from a severe COVID-19 illness. These include worries and fear of infecting others, shame or stigma associated with being infected with COVID-19,[37] and uncertainty of the prognosis.[18] Indeed, the diagnosis of potentially life-threatening diseases, e.g. cancer, has previously been associated with a dramatic rise in risks of psychiatric disorders[38] and suicide.[39] In addition, dysregulated immune response, e.g. the documented cytokine storm associated with COVID-19,[40] may independently affect risks of psychiatric disorders in COVID-19 patients,[41] as has been documented in patients with other infections.[42] In line with Wang et al.,[43] we found that individuals with a higher educational level are more likely than those with a lower educational level to suffer from symptoms of depression and anxiety after a COVID-19 diagnosis. These findings are intriguing and require further investigation as previous studies have also provided contradicting results.[44]

Our mediation analysis suggests that flu-like symptom burden mediated the risk elevations in psychiatric morbidities among patients recovering from COVID-19. Moreover, flu-like symptoms among individuals without a confirmed COVID-19 diagnosis were also strongly associated with

For peer review only - http://bmjopen.bmj.com/site/about/guidelines.xhtml

symptoms of mental morbidities, suggesting that the association is not limited to COVID-19. Indeed, COVID-19 patients reporting low-medium flu-like symptom burden, had lower levels of depression and anxiety than individuals without a diagnosis of COVID-19 but with similar levels of flu-like symptoms. While the healthy pre-pandemic profile of the COVID-19 population may be an indicator of psychological resilience during recovery from COVID-19, it is also possible that the systematic, daily surveillance calls to all COVID-19 patients from the specialized COVID-19 outpatient clinic at the National University Hospital had a positive mental health impact on patients.

This population-based study includes more than 20% of all patients diagnosed with COVID-19 by RT-PCR during the first wave of the epidemic in Iceland. The diagnostic rate in Iceland was among the highest worldwide during the first wave of the epidemic,[45, 46] allowing us to perform sensitivity analyses limited to individuals who all had been screened for SARS-CoV-2. The extensive and well-piloted questionnaire allowed us to obtain validated symptom assessment of mental morbidities along with detailed information on flu-like symptom burden for all individuals during the flu season in Iceland, together with a wide range of potential confounders in the association between COVID-19 and mental morbidities.

However, our study is cross-sectional and therefore less informative on the directionality of the studied associations. Indeed, a newly published study using data from the UK Biobank suggests that individuals with a history of pre-pandemic psychiatric disorders are more likely to be hospitalized and die from COVID-19.[47] Thus, it is possible that patients who were already experiencing psychological symptoms suffered the most severe COVID-19-related symptomology yielding the observed associations. However, we included previous diagnoses of psychiatric disorders in all our models which should, to some extent, alleviate this concern. Nevertheless, it is possible that individuals with severe symptoms of depression and anxiety were more likely to report high levels of flu-like symptoms during the preceding two months when responding to the questionnaire, yielding

For peer review only - http://bmjopen.bmj.com/site/about/guidelines.xhtml

a detrimental outcome-dependent misclassification. Indeed, the instrument to assess flu-like symptom burden included similar items (e.g. fatigue) as are included in the PHQ-9 instrument which, to some extent, could explain the association between COVID-19 and depression. Yet, more objective assessments of illness severity, e.g. the number of days confined to bed or hospitalized due to COVID-19 -- both of which were strongly associated with mental morbidities among patients -- might to a lesser extent be influenced by the participants' mental status.

Participants recovering from COVID-19 may differ in terms of their mental health status from COVID-19 patients who did not participate in our study. While we were not able to disentangle the direction of such selection, our observed prevalence of depression and anxiety among recovering COVID-19 patients resembles prevalence rates recently reported in a meta-analysis of diverse COVID-19 patient populations elsewhere.[9] Finally, our study is nested within a small, economically and socially secure society with virtually free and high-quality health care for all, thus the findings may not be generalizable to other settings.

In conclusion, the relatively healthy first-wave COVID-19 population in Iceland presented with increased risk of depression and PTSD in the early weeks of recovery from their illness, particularly those recovering from a severe disease. These findings motivate further follow-up studies of mental health among patients recovering from COVID-19 and other serious infections, and heightened clinical surveillance of those recovering from a severe illness.

For peer review only - http://bmjopen.bmj.com/site/about/guidelines.xhtml

Contributors: UV, TA, and JJ had full access to all of the data in the study and take responsibility for the integrity of the data and the accuracy of the data analysis. UV, AH, KSS, IM, JJ, TA conceived and design the study. UV, AH, KSS, IM, JJ, TA analysed the data, and all authors (KSS, HYH, IM, AH, EBT, ÁBG, GT, HR, HLJ, BG, GP, PHP, SYK, TJL, SH, HH, GG, EE, DGG, HS, SH, ADM, RP, JJ, TA, UV) interpreted the data. UV, KSS, HYH, IM drafted the manuscript. All authors provided critical revision of the manuscript for important intellectual content. UV, AH, HR, GT, TA, JJ, SYK, TJL, SH, DGG, ADM provided administrative, technical, or material support. The corresponding author attests that all listed authors meet authorship criteria and that no others meeting the criteria have been omitted.

Funding: This study was supported by grants from the Icelandic government and NordForsk (Mental morbidity trajectories in COVID-19 across risk populations of five nations, grant 105668, Dr. UV). The funding sources had no role in the design and implementation of the study; in data collection, management, analysis, and interpretation of the data; preparation, review, or approval of the manuscript; and decision to submit the manuscript for publication.

Competing interests: All authors complete the ICMJE uniform disclosure form at www.icmje.org/coi_disclosure.pdf and declare no support from any organisation for the submitted work (except those described above); no financial relationships with any organisations that might have an interest in the submitted work in the previous three years; no other relationships or activities that could appear to have influenced the submitted work.

Ethical approval: The study was approved by the National Bioethics Committee of Iceland, Reykjavik (NBC no. 20-073) and the Data Protection Authority.

Data sharing: The original data and R codes are available on request upon approval of the National Bioethics Committee and the Data Protection Authority.

For peer review only - http://bmjopen.bmj.com/site/about/guidelines.xhtml

The lead author/guarantor of the study (UV) affirms that this manuscript is an honest, accurate, and transparent account of the study being reported; that no important aspects of the study have been omitted; and that any discrepancies from the study as planned (and, if relevant, registered) have been explained.

The Corresponding Author (UV) has the right to grant on behalf of all authors and does grant on behalf of all authors, an exclusive licence on a worldwide basis to the BMJ Publishing Group Ltd to permit this article (if accepted) to be published in BMJ editions and any other BMJPGL products and sublicences such use and exploit all subsidiary rights, as set out in our licence.

For peer review only

References

1. World Health Organization [WHO]. WHO Coronavirus Disease (COVID-19) Dashboard. 2021. Available from: https://covid19.who.int/
2. Carfì A, Bernabei R, Landi F. Persistent Symptoms in Patients After Acute COVID-19. *JAMA* 2020;324:603-5. doi: 10.1001/jama.2020.12603
3. Carvalho-Schneider C, Laurent E, Lemaignen A, et al. Follow-up of adults with non-critical COVID-19 two months after symptoms' onset. *Clin Microbiol Infect* 2020. doi: 10.1016/j.cmi.2020.09.052
4. Halpin SJ, McIvor C, Whyatt G, et al. Postdischarge symptoms and rehabilitation needs in survivors of COVID-19 infection: A cross-sectional evaluation. *J Med Virol* 2020. doi: 10.1002/jmv.26368
5. Tolba M, Abo Omirah M, Hussein A, et al. Assessment and characterisation of post-COVID-19 manifestations. *Int J Clin Pract* 2020:e13746. doi: 10.1111/ijcp.13746
6. Xiong Q, Xu M, Li J, et al. Clinical sequelae of COVID-19 survivors in Wuhan, China: a single-centre longitudinal study. *Clin Microbiol Infect* 2020. doi: 10.1016/j.cmi.2020.09.023
7. Moldofsky H, Patcai J. Chronic widespread musculoskeletal pain, fatigue, depression and disordered sleep in chronic post-SARS syndrome; a case-controlled study. *BMC Neurol* 2011;11:37. doi: 10.1186/1471-2377-11-37
8. Ahmed H, Patel K, Greenwood DC, et al. Long-term clinical outcomes in survivors of severe acute respiratory syndrome and Middle East respiratory syndrome coronavirus outbreaks after hospitalisation or ICU admission: A systematic review and meta-analysis. *J Rehabil Med* 2020;52:jrm00063. doi: 10.2340/16501977-2694
9. Deng J, Zhou F, Hou W, et al. The prevalence of depression, anxiety, and sleep disturbances in COVID-19 patients: a meta-analysis. *Ann N Y Acad Sci* 2020. doi: 10.1111/nyas.14506
10. Bo HX, Li W, Yang Y, et al. Posttraumatic stress symptoms and attitude toward crisis mental health services among clinically stable patients with COVID-19 in China. *Psychol Med* 2020:1-2. doi: 10.1017/s0033291720000999
11. Krishnamoorthy Y, Nagarajan R, Saya GK, et al. Prevalence of psychological morbidities among general population, healthcare workers and COVID-19 patients amidst the COVID-19 pandemic: A systematic review and meta-analysis. *Psychiatry Res* 2020;293:113382. doi: 10.1016/j.psychres.2020.113382
12. Mazza MG, De Lorenzo R, Conte C, et al. Anxiety and depression in COVID-19 survivors: Role of inflammatory and clinical predictors. *Brain Behav Immun* 2020;89:594-600. doi: 10.1016/j.bbi.2020.07.037
13. Taquet M, Luciano S, Geddes JR, et al. Bidirectional associations between COVID-19 and psychiatric disorder: retrospective cohort studies of 62 354 COVID-19 cases in the USA. *Lancet Psychiatry* 2020. doi: 10.1016/s2215-0366(20)30462-4
14. Huang C, Huang L, Wang Y, et al. 6-month consequences of COVID-19 in patients discharged from hospital: a cohort study. *Lancet* 2021;397:220-32. doi: 10.1016/s0140-6736(20)32656-8
15. Todt BC, Szlejf C, Duim E, et al. Clinical outcomes and quality of life of COVID-19 survivors: A follow-up of 3 months post hospital discharge. *Respir Med* 2021;184:106453. doi: 10.1016/j.rmed.2021.106453
16. Wang C, Pan R, Wan X, et al. Immediate Psychological Responses and Associated Factors during the Initial Stage of the 2019 Coronavirus Disease (COVID-19) Epidemic among the

For peer review only

General Population in China. *Int J Environ Res Public Health* 2020;17:1729. doi: 10.3390/ijerph17051729
17. Wang C, Pan R, Wan X, et al. A longitudinal study on the mental health of general population during the COVID-19 epidemic in China. *Brain Behav Immun* 2020;87:40-8. doi: 10.1016/j.bbi.2020.04.028
18. Sahoo S, Mehra A, Dua D, et al. Psychological experience of patients admitted with SARS-CoV-2 infection. *Asian J Psychiatr* 2020;54:102355. doi: 10.1016/j.ajp.2020.102355
19. Dantzer R, O'Connor JC, Freund GG, et al. From inflammation to sickness and depression: when the immune system subjugates the brain. *Nature Reviews Neuroscience* 2008;9:46-56. doi: 10.1038/nrn2297
20. Huang C, Wang Y, Li X, et al. Clinical features of patients infected with 2019 novel coronavirus in Wuhan, China. *The Lancet* 2020;395:497-506. doi: 10.1016/S0140-6736(20)30183-5
21. Bennett FC, Molofsky AV. The immune system and psychiatric disease: a basic science perspective. *Clinical & Experimental Immunology* 2019;197:294-307. doi: https://doi.org/10.1111/cei.13334
22. Helgason D, Eythorsson E, Olafsdottir LB, et al. Beating the odds with systematic individualized care: Nationwide prospective follow-up of all patients with COVID-19 in Iceland. *J Intern Med* 2020. doi: 10.1111/joim.13135
23. Icelandic Directorate of Health and the Department of Civil Protection and Emergency Management. COVID-19 in Iceland – Statistics 28 Feb - 14 June 2020. 2020. Available from: https://www.covid.is/data-old
24. COVID-19 National Resilience Cohort. About the study. 2020. Available from: https://lidanicovid.is/about-the-study/
25. Statistics Iceland. Population by sex and age 1841-2020. 2020. Available from: https://px.hagstofa.is/pxen/pxweb/en/Ibuar/Ibuar__mannfjoldi__1_yfirlit__yfirlit_mannfjolda/MAN00101.px/?rxid=f6dc8cfa-1b31-43cb-8f39-cec23335bd55
26. Central Bank of Iceland. Official exchange rate. 2020. Available from: https://www.cb.is/statistics/official-exchange-rate/
27. Centers for Disease Control and Prevention. Binge Drinking. 2019. Available from: https://www.cdc.gov/alcohol/fact-sheets/binge-drinking.htm
28. Chen N, Zhou M, Dong X, et al. Epidemiological and clinical characteristics of 99 cases of 2019 novel coronavirus pneumonia in Wuhan, China: a descriptive study. *Lancet* 2020;395:507-13. doi: 10.1016/s0140-6736(20)30211-7
29. Wang D, Hu B, Hu C, et al. Clinical Characteristics of 138 Hospitalized Patients With 2019 Novel Coronavirus-Infected Pneumonia in Wuhan, China. *JAMA* 2020;323:1061-9. doi: 10.1001/jama.2020.1585
30. Kroenke K, Spitzer RL, Williams JB. The PHQ-9: Validity of a brief depression severity measure. *J Gen Intern Med* 2001;16:606-13. doi: 10.1046/j.1525-1497.2001.016009606.x
31. Spitzer RL, Kroenke K, Williams JBW, et al. A brief measure for assessing generalized anxiety disorder: The GAD-7. *Arch Intern Med* 2006;166:1092-7. doi: 10.1001/archinte.166.10.1092
32. Prins A, Bovin MJ, Smolenski DJ, et al. The Primary Care PTSD Screen for DSM-5 (PC-PTSD-5): Development and Evaluation Within a Veteran Primary Care Sample. *J Gen Intern Med* 2016;31:1206-11. doi: 10.1007/s11606-016-3703-5
33. Zou G. A modified poisson regression approach to prospective studies with binary data. *Am J Epidemiol* 2004;159:702-6. doi: 10.1093/aje/kwh090

For peer review only - http://bmjopen.bmj.com/site/about/guidelines.xhtml

34. van Buuren S, Groothuis-Oudshoorn K. mice: Multivariate Imputation by Chained Equations in R. *J Stat Softw* 2011;45:1-67. doi:
35. Statistics Iceland. Educational attainment of the population by levels according to ISCED 2011 2003-2019. 2020. Available from: https://px.hagstofa.is/pxen/pxweb/en/Samfelag/Samfelag__skolamal__5_menntunarstada/SKO00007.px/table/tableViewLayout1/?rxid=e20daaf9-d20a-4c69-be63-13b67f196152
36. Statistics Iceland. Population by urban nuclei, sex and age 1 January 2001-2021. 2021. doi:
37. Liu D, Baumeister RF, Veilleux JC, et al. Risk factors associated with mental illness in hospital discharged patients infected with COVID-19 in Wuhan, China. *Psychiatry Res* 2020;292:113297. doi: 10.1016/j.psychres.2020.113297
38. Lu D, Andersson TM, Fall K, et al. Clinical Diagnosis of Mental Disorders Immediately Before and After Cancer Diagnosis: A Nationwide Matched Cohort Study in Sweden. *JAMA Oncol* 2016;2:1188-96. doi: 10.1001/jamaoncol.2016.0483
39. Fang F, Fall K, Mittleman MA, et al. Suicide and cardiovascular death after a cancer diagnosis. *N Engl J Med* 2012;366:1310-8. doi: 10.1056/NEJMoa1110307
40. Huang C, Wang Y, Li X, et al. Clinical features of patients infected with 2019 novel coronavirus in Wuhan, China. *Lancet* 2020;395:497-506. doi: 10.1016/s0140-6736(20)30183-5
41. Dantzer R, O'Connor JC, Freund GG, et al. From inflammation to sickness and depression: when the immune system subjugates the brain. *Nat Rev Neurosci* 2008;9:46-56. doi: 10.1038/nrn2297
42. Raison CL, Capuron L, Miller AH. Cytokines sing the blues: inflammation and the pathogenesis of depression. *Trends Immunol* 2006;27:24-31. doi: 10.1016/j.it.2005.11.006
43. Wang Y, Di Y, Ye J, et al. Study on the public psychological states and its related factors during the outbreak of coronavirus disease 2019 (COVID-19) in some regions of China. *Psychol Health Med* 2021;26:13-22. doi: 10.1080/13548506.2020.1746817
44. Hossain MM, Tasnim S, Sultana A, et al. Epidemiology of mental health problems in COVID-19: a review. *F1000Res* 2020;9:636-. doi: 10.12688/f1000research.24457.1
45. Hasell J O-OE, Mathieu E, Ritchie H, Beltekian D, Roser M. To understand the global pandemic, we need global testing – the Our World in Data COVID-19 Testing dataset. March 31, 2020. Available from: https://ourworldindata.org/covid-testing
46. Gudbjartsson DF, Norddahl GL, Melsted P, et al. Humoral Immune Response to SARS-CoV-2 in Iceland. *N Engl J Med* 2020;383:1724-34. doi: 10.1056/NEJMoa2026116
47. Yang H, Chen W, Hu Y, et al. Pre-pandemic psychiatric disorders and risk of COVID-19: a UK Biobank cohort analysis. *Lancet Healthy Longevity* 2020;1:e69-e79. doi: 10.1016/S2666-7568(20)30013-1

Figure legends

Figure 1: The proportion of individuals with and without (tested and not tested) a confirmed diagnosis of COVID-19 with flu-like symptoms of one week or more during the last two months before responding to the baseline questionnaire.

For peer review only - http://bmjopen.bmj.com/site/about/guidelines.xhtml

Figure 2: Adjusted symptom scores of depression, anxiety and PTSD by flu-like symptom burden among individuals with a recent COVID-19 diagnosis, those who tested negative and those never tested for SARS-CoV-2.

For peer review only

For peer review only - http://bmjopen.bmj.com/site/about/guidelines.xhtml

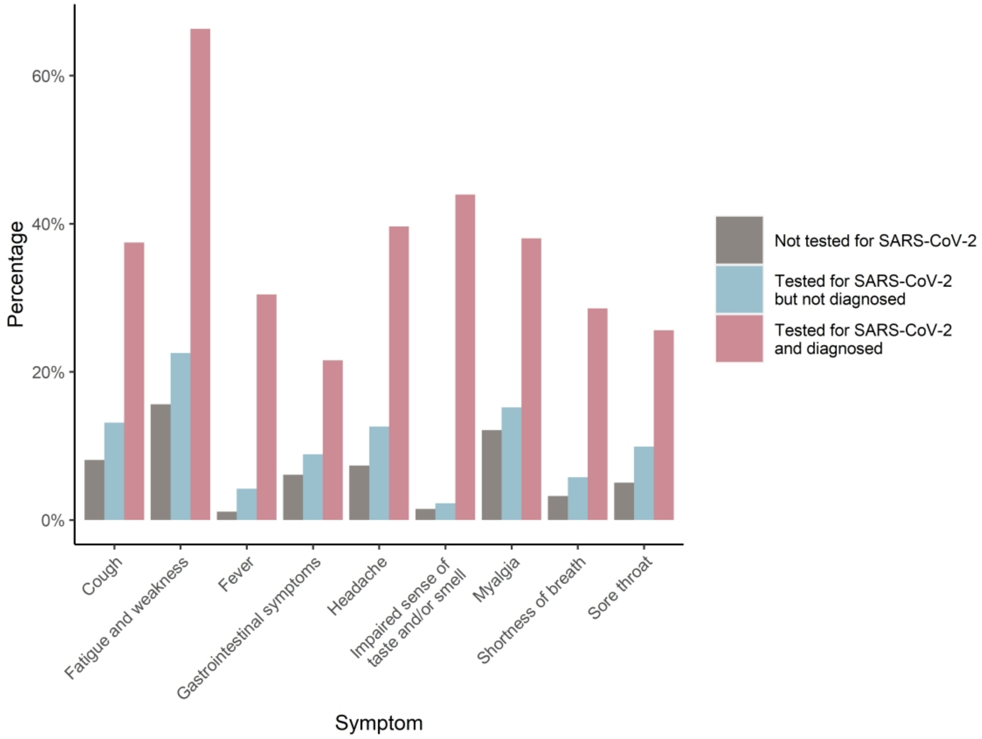

Figure 1 / The proportion of individuals with and without (tested and not tested) a confirmed diagnosis of COVID-19 with flu-like symptoms of one week or more during the last two months before responding to the baseline questionnaire.

203x152mm (600 x 600 DPI)

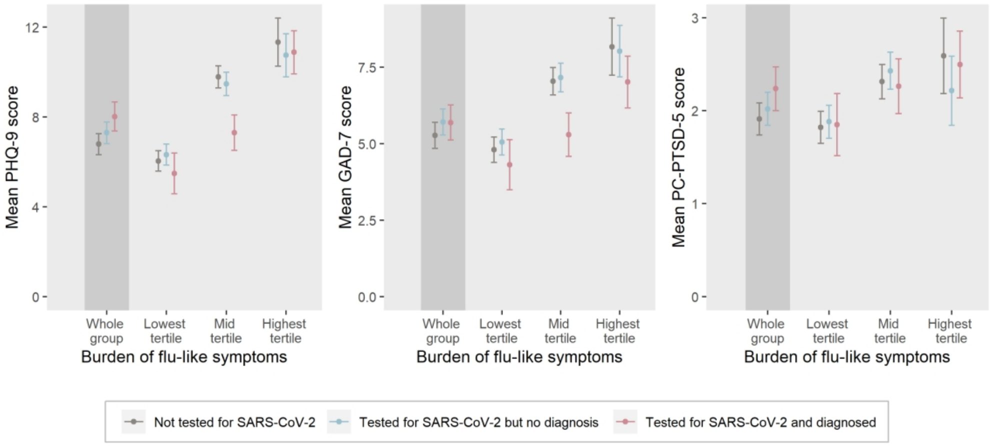

Figure 2 / Adjusted symptom scores of depression, anxiety and PTSD by flu-like symptom burden among individuals with a recent COVID-19 diagnosis, those who tested negative and those never tested for SARS-CoV-2.

254x127mm (300 x 300 DPI)

Supplementary material

Supplementary Figure 1. The analytic sample within the COVID-19 National Resilience Cohort.

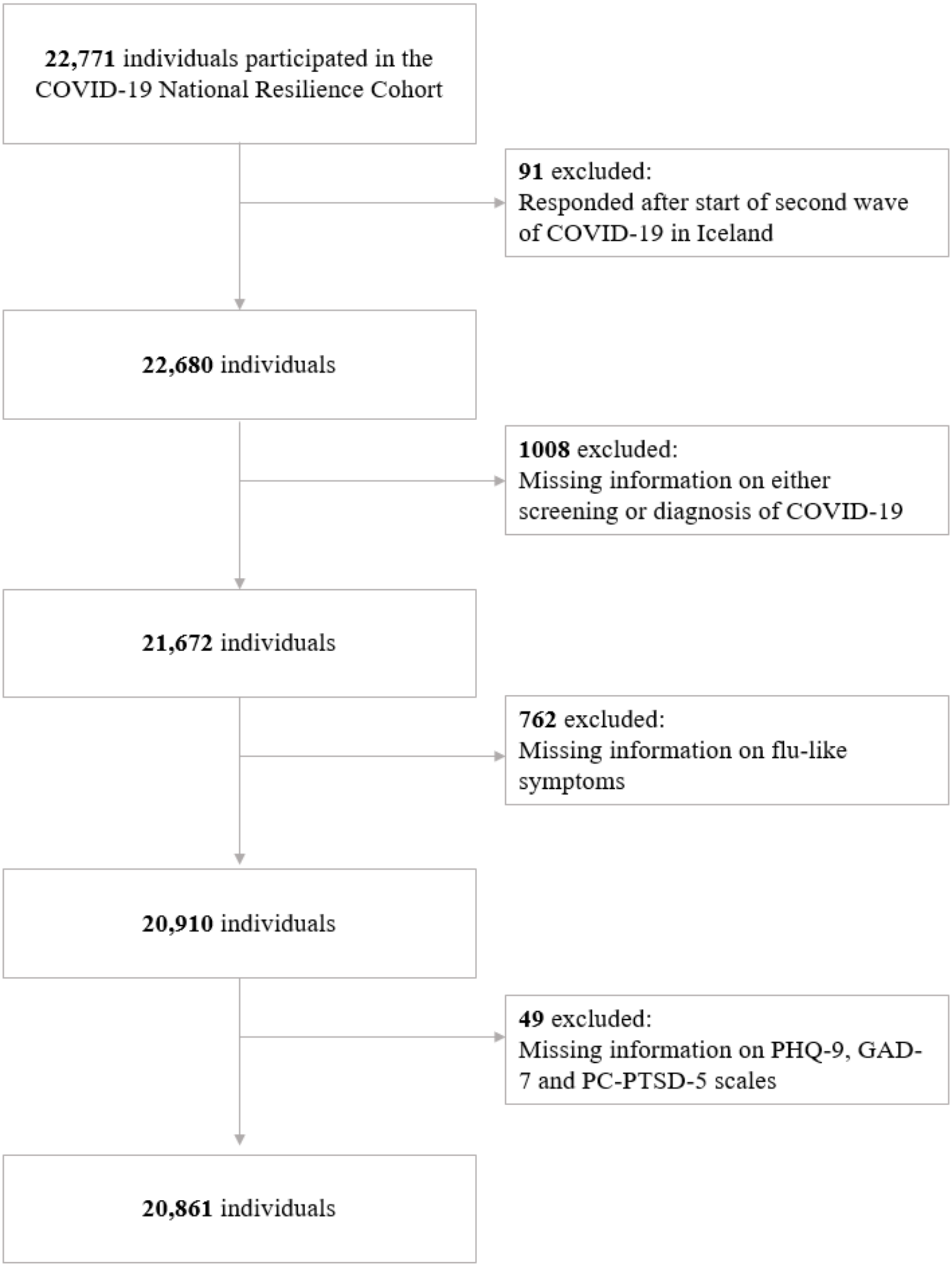

Supplementary Table 1. Mean and median scores on PHQ-9, GAD-7, and PC-PTSD-5 among individuals with and without a recent COVID-19 diagnosis.

	Mean scores		Median scores		P-value	Adj. P-value
	**Individuals not diagnosed with COVID-19**	**Individuals diagnosed with COVID-19**	**Individuals not diagnosed with COVID-19**	**Individuals diagnosed with COVID-19**		
**Depression**	5.07	6.11	4	5	<.001	<.001
**Anxiety**	4.12	4.52	3	4	.07	.08
**PTSD**	1.72	1.90	2	2	.007	<.001

[a] Adjusted for age (continuous variable), gender (male, female, or other), educational level (compulsory education, high school/trade school/other education, bachelor's degree/diploma certificate, or master's/Ph.D. degree), income (continuous variable), current smoking (yes or no), previous diagnosis of a psychiatric disorder (yes or no), number of previously diagnosed somatic diseases (0, 1, or ≥ 2), and timing of responding to the baseline questionnaire (April 24-30, May 1-7, May 8-23, or May 24-July 22).

For peer review only

Supplementary Table 2. The prevalence, crude and multivariable adjusted relative risks and absolute differences in symptoms of depression (PHQ-9), anxiety (GAD-7) and PTSD (PC-PTSD-5) surpassing screening thresholds among individuals with and without a confirmed diagnosis of COVID-19. The data are presented with stratification of all covariates used in the multivariable adjusted models along with the p-values for the effect modification.

	No. (%)						
	Individuals not diagnosed with COVID-19	Individuals diagnosed with COVID-19	cRR (95% CI)	aRR (95% CI)[a]	Absolute difference Crude %	Absolute difference Adjusted [a] %	P-value for effect modification
**Depression**	2992 (16.2)	75 (22.1)	1.36 (1.11 to 1.67)	1.48 (1.20 to 1.82)	5.9	8.4	
**Female**	2421 (18.8)	56 (24.8)	1.32 (1.05 to 1.66)	1.43 (1.13 to 1.81)	6.0	8.1	0.110
**Male**	559 (10.0)	17 (15.2)	1.52 (0.97 to 2.37)	1.54 (0.96 to 2.47)	5.2	7.2	--
**18-39 years**	954 (33.3)	23 (27.1)	0.81 (0.57 to 1.16)	0.94 (0.65 to 1.36)	-6.2	-1.8	0.025
**40-59 years**	1367 (16.8)	40 (22.3)	1.33 (1.01 to 1.75)	1.72 (1.31 to 2.27)	5.5	14.7	--
**≥60 years**	671 (8.9)	12 (15.8)	1.77 (1.05 to 2.98)	2.57 (1.52 to 4.36)	6.9	21.8	--
**Compulsory education**	540 (21.5)	6 (23.1)	1.07 (0.53-2.17)	1.24 (0.61-2.53)	1.6	4.7	<.001
**Upper secondary, vocational or other education**	996 (17.7)	15 (14.7)	0.83 (0.52-1.33)	0.83 (0.51-1.33)	-2.9	-2.5	--
**Bachelor's degree or diploma certificate**	935 (15.7)	30 (24.2)	1.54 (1.12-2.12)	1.56 (1.16-2.08)	8.5	10.2	--
**Master's or Ph.D. degree**	521 (11.9)	24 (27.3)	2.28 (1.61-3.24)	2.95 (2.03-4.29)	15.3	34.5	--
**Low income**	966 (29.8)	15 (31.3)	1.05 (0.69-1.60)	0.97 (0.61-1.55)	1.5	-0.8	0.059
**Low-medium income**	956 (18.1)	21 (25.3)	1.40 (0.96-2.04)	1.36 (0.97-1.89)	7.2	8.3	--
**Medium income**	637 (13.4)	18 (23.4)	1.75 (1.16-2.64)	1.66 (1.09-2.55)	10.0	12.6	--
**Medium-high income**	316 (9.3)	13 (15.1)	1.63 (0.98-2.72)	2.04 (1.24-3.35)	5.8	13.1	--
**High income**	117 (6.6)	8 (17.3)	2.64 (1.37-5.08)	2.90 (1.56-5.37)	10.8	15.3	--

**Not current smoker**	2351 (14.5)	71 (22.0)	1.52 (1.23-1.87)	1.53 (1.23-1.89)	7.6	8.0	0.234
**Current smoker**	641 (28.3)	4 (22.2)	0.79 (0.33-1.87)	0.91 (0.36-2.33)	-6.1	-1.6	--
**No previous diagnosis of psychiatric disorder**	1053 (8.0)	38 (14.6)	1.83 (1.36-2.47)	1.55 (1.13-2.12)	6.6	5.6	0.176
**Previous diagnosis of psychiatric disorder**	1939 (37.0)	37 (46.8)	1.27 (1.00-1.61)	1.36 (1.04-1.77)	9.9	13.6	--
**No previously diagnosed somatic diseases**	1776 (16.2)	54 (22.4)	1.38 (1.09-1.75)	1.52 (1.19-1.95)	6.2	7.7	0.923
**1 previously diagnosed somatic disease**	785 (14.6)	17 (20.7)	1.42 (0.92-2.18)	1.38 (0.91-2.10)	6.1	6.2	--
**≥ 2 previously diagnosed somatic diseases**	431 (19.8)	4 (23.5)	1.19 (0.50-2.81)	1.88 (0.70-5.06)	3.7	14.7	--
**Response date: April 24-May 7**	2206 (18.3)	45 (27.4)	1.50 (1.16-1.93)	1.62 (1.24-2.11)	9.1	12.6	0.531
**Response date: May 8-July 22**	786 (12.2)	30 (17.0)	1.40 (1.00-1.95)	1.24 (0.89-1.71)	4.9	3.2	--
**Anxiety**	2120 (11.3)	45 (13.1)	1.16 (0.88 to 1.53)	1.24 (0.93 to 1.64)	1.8	2.6	
**Female**	1759 (13.4)	34 (14.8)	1.11 (0.81 to 1.51)	1.17 (0.84 to 1.62)	1.4	2.2	0.007
**Male**	353 (6.2)	10 (8.9)	1.43 (0.78 to 2.60)	1.39 (0.76 to 2.54)	2.6	3.0	--
**18-39 years**	770 (26.2)	13 (14.9)	0.57 (0.34 to 0.95)	0.61 (0.36 to 1.04)	-11.3	-9.3	0.049
**40-59 years**	951 (11.5)	27 (14.7)	1.28 (0.90 to 1.82)	1.70 (1.20 to 2.41)	3.2	10.5	--
**≥60 years**	399 (5.2)	5 (6.9)	1.31 (0.56 to 3.06)	2.13 (0.91 to 5.00)	1.7	7.5	--
**Compulsory education**	378 (14.7)	2 (8.0)	0.54 (0.14-2.06)	0.75 (0.21-2.69)	-6.7	-2.7	0.009
**Upper secondary, vocational or other education**	690 (12.0)	11 (10.5)	0.87 (0.50-1.54)	0.84 (0.48-1.48)	-1.5	-1.4	--
**Bachelor's degree or diploma certificate**	658 (10.9)	17 (13.4)	1.23 (0.79-1.93)	1.18 (0.74-1.86)	2.5	2.0	--
**Master's or Ph.D. degree**	394 (8.9)	15 (17.2)	1.94 (1.21-3.11)	2.47 (1.53-3.99)	8.4	19.6	--
**Low income**	719 (21.6)	6 (12.5)	0.58 (0.27-1.23)	0.52 (0.24-1.12)	-9.1	-8.9	0.082
**Low-medium income**	657 (12.2)	17 (19.8)	1.62 (1.06-2.50)	1.44 (0.95-2.20)	7.6	6.6	--

**Medium income**	432 (8.9)	11 (14.5)	1.62 (0.93-2.82)	1.58 (0.89-2.81)	5.5	6.1	--
**Medium-high income**	223 (6.5)	9 (10.3)	1.60 (0.85-3.01)	2.05 (1.03-4.09)	3.9	8.0	--
**High income**	89 (5.0)	2 (4.3)	0.86 (0.22-3.38)	0.97 (0.25-3.73)	-0.7	-0.1	--
**Not current smoker**	1641 (10.0)	43 (13.2)	1.33 (1.00-1.76)	1.31 (0.98-1.76)	3.3	3.0	0.193
**Current smoker**	479 (20.6)	2 (10.5)	0.51 (0.14-1.90)	0.51 (0.13-1.98)	-10.1	-6.1	--
**No previous diagnosis of psychiatric disorder**	709 (5.3)	24 (9.2)	1.73 (1.18-2.55)	1.46 (0.97-2.19)	3.9	2.5	0.130
**Previous diagnosis of psychiatric disorder**	1411 (26.1)	21 (25.6)	0.98 (0.68-1.42)	1.03 (0.70-1.52)	-0.5	0.8	--
**No previously diagnosed somatic diseases**	1320 (11.9)	34 (13.9)	1.17 (0.85-1.60)	1.22 (0.88-1.71)	2.0	2.2	0.651
**1 previously diagnosed somatic disease**	519 (9.5)	10 (12.3)	1.30 (0.72-2.34)	1.31 (0.71-2.41)	2.9	3.0	--
**≥ 2 previously diagnosed somatic diseases**	281 (12.6)	1 (5.6)	0.44 (0.07-2.97)	0.75 (0.22-2.52)	-7.0	-2.7	--
**Response date: April 24-May 7**	1594 (13.0)	28 (16.7)	1.28 (0.91-1.80)	1.37 (0.97-1.94)	3.7	4.7	0.622
**Response date: May 8-July 22**	526 (8.0)	17 (9.7)	1.21 (0.76-1.91)	0.93 (0.57-1.52)	1.7	-0.6	--
**PTSD**	2699 (15.6)	59 (19.5)	1.25 (0.99 to 1.57)	1.38 (1.09 to 1.75)	3.9	7.2	
**Female**	2198 (18.6)	46 (22.7)	1.22 (0.94 to 1.58)	1.32 (1.02 to 1.71)	4.1	6.7	<.001
**Male**	493 (9.1)	12 (12.1)	1.33 (0.78 to 2.27)	1.60 (0.92 to 2.77)	3.0	7.1	--
**18-39 years**	597 (24.2)	11 (15.3)	0.63 (0.37 to 1.09)	0.77 (0.45 to 1.32)	-8.9	-6.2	0.003
**40-59 years**	1268 (16.7)	31 (19.3)	1.15 (0.84 to 1.59)	1.36 (0.99 to 1.88)	2.6	7.8	--
**≥60 years**	834 (11.6)	17 (24.3)	2.09 (1.38 to 3.18)	2.67 (1.78 to 4.01)	12.7	27.9	--
**Compulsory education**	367 (16.1)	7 (33.3)	2.08 (1.13-3.83)	2.41 (1.19-4.89)	17.2	24.8	0.239
**Upper secondary, vocational or other education**	809 (15.5)	20 (21.1)	1.36 (0.92-2.02)	1.44 (0.96-2.18)	5.6	9.1	--
**Bachelor's degree or diploma certificate**	912 (16.4)	23 (21.1)	1.29 (0.89-1.86)	1.34 (0.93-1.94)	4.7	4.8	--

**Master's or Ph.D. degree**	611 (14.7)	9 (11.5)	0.79 (0.42-1.46)	1.01 (0.55-1.84)	-3.1	0.2	--
**Low income**	602 (21.0)	10 (22.7)	1.08 (0.62-1.87)	1.11 (0.64-1.93)	1.7	2.2	0.756
**Low-medium income**	773 (15.8)	17 (23.6)	1.49 (0.98-2.27)	1.53 (0.98-2.37)	7.8	9.9	--
**Medium income**	726 (16.1)	12 (17.4)	1.08 (0.64-1.82)	1.16 (0.68-1.98)	1.3	2.8	--
**Medium-high income**	403 (12.3)	14 (17.9)	1.46 (0.90-2.36)	1.77 (1.14-2.76)	5.6	16.4	--
**High income**	195 (11.4)	6 (15.0)	1.32 (0.62-2.79)	1.53 (0.72-3.26)	3.6	6.7	--
**Not current smoker**	2244 (14.7)	51 (17.9)	1.22 (0.95-1.56)	1.28 (0.99-1.65)	3.2	4.5	0.054
**Current smoker**	455 (22.6)	8 (44.4)	1.97 (1.17-3.32)	2.83 (1.57-5.10)	21.9	37.4	--
**No previous diagnosis of psychiatric disorder**	1540 (12.2)	42 (17.6)	1.45 (1.10-1.92)	1.55 (1.17-2.06)	5.5	7.5	0.137
**Previous diagnosis of psychiatric disorder**	1159 (25.3)	17 (26.2)	1.04 (0.69-1.56)	1.11 (0.73-1.68)	0.9	3.0	--
**No previously diagnosed somatic diseases**	1611 (15.8)	41 (19.4)	1.23 (0.93-1.62)	1.44 (1.09-1.90)	3.6	7.6	0.325
**1 previously diagnosed somatic disease**	751 (14.8)	12 (16.0)	1.08 (0.64-1.82)	1.11 (0.66-1.87)	1.2	1.8	--
**≥ 2 previously diagnosed somatic diseases**	337 (16.7)	6 (35.3)	2.11 (1.10-4.04)	2.03 (0.94-4.40)	18.6	17.5	--
**Response date: April 24-May 7**	1996 (18.1)	32 (22.5)	1.25 (0.92-1.70)	1.24 (0.91-1.70)	4.5	5.8	0.256
**Response date: May 8-July 22**	703 (11.4)	27 (16.8)	1.47 (1.04-2.09)	1.41 (0.99-2.01)	5.4	6.0	--

[a] Adjusted for age (continuous variable), gender (male, female, or other), educational level (compulsory education, high school/trade school/other education, bachelor's degree/diploma certificate, or master's/Ph.D. degree), income (continuous variable), current smoking (yes or no), previous diagnosis of a psychiatric disorder (yes or no), number of previously diagnosed somatic diseases (0, 1, or ≥ 2), and timing of responding to the baseline questionnaire (April 24-30, May 1-7, May 8-23, or May 24-July 22). Adjustments are not made for the variable that is being stratified by.

For peer review only - http://bmjopen.bmj.com/site/about/guidelines.xhtml

Supplementary Table 3. The prevalence, crude and multivariable adjusted relative risks and absolute differences in symptoms of depression (PHQ-9), anxiety (GAD-7) and PTSD (PC-PTSD-5) surpassing screening thresholds among individuals with and without a confirmed diagnosis of COVID-19 (*diagnosed at least two weeks before responding)*.

	No. (%)					
	Individuals not diagnosed with COVID-19	Individuals diagnosed with COVID-19	cRR (95% CI)	aRR (95% CI)[a]	Absolute difference Crude %	Absolute difference Adjusted [a] %
**Depression**	2992 (16.2)	73 (22.0)	1.36 (1.11 to 1.67)	1.49 (1.20 to 1.84)	5.8	8.6
**Female**	2421 (18.8)	55 (24.9)	1.32 (1.05 to 1.67)	1.45 (1.14 to 1.83)	6.1	8.4
**Male**	559 (10.0)	16 (14.7)	1.47 (0.93 to 2.33)	1.52 (0.93 to 2.49)	4.7	6.9
**18-39 years**	954 (33.3)	22 (26.8)	0.81 (0.56 to 1.16)	0.95 (0.65 to 1.39)	-6.5	-1.5
**40-59 years**	1367 (16.8)	39 (22.3)	1.32 (1.00 to 1.75)	1.72 (1.30 to 2.28)	5.5	14.7
**≥60 years**	671 (8.9)	12 (16.0)	1.79 (1.06 to 3.02)	2.59 (1.53 to 4.39)	7.1	22.0
**Anxiety**	2120 (11.3)	45 (13.1)	1.16 (0.88 to 1.54)	1.25 (0.94 to 1.67)	1.8	2.9
**Female**	1759 (13.4)	33 (14.7)	1.10 (0.80 to 1.51)	1.17 (0.84 to 1.64)	1.3	2.3
**Male**	353 (6.2)	10 (9.1)	1.46 (0.80 to 2.67)	1.46 (0.80 to 2.64)	2.9	3.5
**18-39 years**	770 (26.2)	13 (15.5)	0.59 (0.36 to 0.98)	0.64 (0.38 to 1.09)	-10.7	-8.6
**40-59 years**	951 (11.5)	26 (14.4)	1.26 (0.88 to 1.80)	1.68 (1.18 to 2.41)	2.9	10.2
**≥60 years**	399 (5.2)	5 (6.9)	1.32 (0.57 to 3.10)	2.15 (0.91 to 5.03)	1.7	7.5
**PTSD**	2699 (15.6)	56 (18.9)	1.21 (0.95 to 1.54)	1.35 (1.06 to 1.73)	3.3	6.7
**Female**	2198 (18.6)	43 (21.7)	1.17 (0.89 to 1.53)	1.27 (0.97 to 1.67)	3.1	5.7
**Male**	493 (9.1)	12 (12.4)	1.36 (0.79 to 2.32)	1.64 (0.95 to 2.83)	3.3	7.5
**18-39 years**	597 (24.2)	10 (14.3)	0.59 (0.33 to 1.05)	0.72 (0.41 to 1.28)	-9.9	-7.4
**40-59 years**	1268 (16.7)	29 (18.5)	1.11 (0.79 to 1.54)	1.31 (0.94 to 1.84)	1.8	6.7
**≥60 years**	834 (11.6)	17 (24.6)	2.12 (1.40 to 3.23)	2.70 (1.80 to 4.05)	13.0	28.3

[a] Adjusted for age (continuous variable), gender (male, female, or other), educational level (compulsory education, high school/trade school/other education, bachelor's degree/diploma certificate, or master's/Ph.D. degree), income (continuous variable), current smoking (yes or no), previous diagnosis of a psychiatric disorder (yes or no), number of previously diagnosed somatic diseases (0, 1, or ≥ 2), and timing of responding to the baseline questionnaire (April 24-30, May 1-7, May 8-23, or May 24-July 22). Adjustments are not made for the variable that is being stratified by.

Supplementary Table 4. The prevalence, crude and multivariable adjusted relative risks and absolute differences in symptoms of depression (PHQ-9), anxiety (GAD-7) and PTSD (PC-PTSD-5) surpassing screening thresholds among individuals with and without a confirmed diagnosis of COVID-19 (*using multiple imputation*).

	No. (%)					
	Individuals not diagnosed with COVID-19	Individuals diagnosed with COVID-19	cRR (95% CI)	aRR (95% CI)[a]	Absolute difference Crude %	Absolute difference Adjusted[a] %
**Depression**	3487 (17.0)	83 (22.3)	1.31 (1.08 to 1.59)	1.44 (1.18 to 1.75)	5.3	8.1
**Female**	2819 (19.7)	64 (25.6)	1.29 (1.05 to 1.61)	1.43 (1.14 to 1.78)	5.9	8.3
**Male**	655 (10.7)	17 (14.1)	1.32 (0.85 to 2.08)	1.35 (0.85 to 2.16)	3.4	4.6
**18-39 years**	1105 (35.2)	26 (28.9)	0.82 (0.59 to 1.14)	0.95 (0.67 to 1.33)	-6.3	-1.8
**40-59 years**	1565 (17.5)	445(22.5)	1.29 (0.99 to 1.68)	1.68 (1.29 to 2.18)	5.0	14.8
**≥60 years**	817 (9.7)	12 (14.8)	1.52 (0.90 to 2.58)	2.23 (1.31 to 3.78)	5.1	17.2
**Anxiety**	2328 (11.3)	45 (12.1)	1.06 (0.81 to 1.40)	1.15 (0.87 to 1.53)	0.8	1.8
**Female**	1940 (13.5)	34 (13.6)	1.01 (0.73 to 1.38)	1.08 (0.78 to 1.50)	0.1	1.0
**Male**	378 (6.2)	10 (8.3)	1.34 (0.73 to 2.45)	1.34 (0.74 to 2.43)	2.1	2.6
**18-39 years**	830 (26.5)	13 (14.4)	0.55 (0.33 to 0.91)	0.61 (0.36 to 1.03)	-12.1	-10.0
**40-59 years**	1037 (11.6)	27 (13.6)	1.18 (0.83 to 1.68)	1.57 (1.11 to 2.23)	2.0	9.0
**≥60 years**	461 (5.5)	5 (5.9)	1.08 (0.46 to 2.53)	1.69 (0.72 to 3.97)	0.4	4.6
**PTSD**	2915 (15.8)	62 (19.2)	1.22 (0.97 to 1.53)	1.36 (1.08 to 1.71)	3.4	6.7
**Female**	2375 (18.7)	48 (22.1)	1.18 (0.92 to 1.52)	1.29 (1.00 to 1.66)	3.4	6.1
**Male**	532 (9.2)	13 (12.4)	1.34 (0.80 to 2.25)	1.57 (0.93 to 2.67)	3.2	6.9
**18-39 years**	642 (24.5)	11 (14.9)	0.61 (0.35 to 1.05)	0.71 (0.41 to 1.22)	-9.6	-7.9
**40-59 years**	1360 (16.8)	33 (19.3)	1.15 (0.84 to 1.57)	1.37 (1.00 to 1.87)	2.5	7.9
**≥60 years**	913 (11.8)	18 (23.1)	1.96 (1.30 to 2.96)	2.47 (1.65 to 3.69)	11.3	23.9

[a] Adjusted for age (continuous variable), gender (male, female, or other), educational level (compulsory education, high school/trade school/other education, bachelor's degree/diploma certificate, or master's/Ph.D. degree), income (continuous variable), current smoking (yes or no), previous diagnosis of a psychiatric disorder (yes or no), number of previously diagnosed somatic diseases (0, 1, or ≥ 2), and timing of responding to the baseline questionnaire (April 24-30, May 1-7, May 8-23, or May 24-July 22). Adjustments are not made for the variable that is being stratified by.

Supplementary Table 5. The prevalence, crude and multivariable adjusted relative risks and absolute differences in symptoms of depression (PHQ-9), anxiety (GAD-7) and PTSD (PC-PTSD-5) surpassing screening thresholds among individuals with and without a confirmed diagnosis of COVID-19 (*analysis limited to individuals who were tested for COVID-19*).

	No. (%)					
	Individuals that have been tested for COVID-19 but not diagnosed	Individuals that have been tested for COVID-19 and diagnosed	cRR (95% CI)	aRR (95% CI)[a]	Absolute difference Crude %	Absolute difference Adjusted[a] %
**Depression**	885 (19.4)	75 (22.1)	1.14 (0.92 to 1.40)	1.31 (1.06 to 1.62)	2.7	6.1
**Female**	712 (21.7)	56 (24.8)	1.14 (0.90 to 1.45)	1.30 (1.02 to 1.65)	3.1	6.2
**Male**	168 (13.2)	17 (15.2)	1.15 (0.72 to 1.82)	1.22 (0.75 to 1.97)	2.0	3.7
**18-39 years**	275 (37.6)	23 (27.1)	0.72 (0.50 to 1.03)	0.85 (0.58 to 1.23)	-10.5	-5.2
**40-59 years**	413 (17.8)	40 (22.3)	1.26 (0.94 to 1.68)	1.64 (1.23 to 2.17)	4.5	13.6
**≥60 years**	197 (13.1)	12 (15.8)	1.21 (0.71 to 2.07)	1.86 (1.09 to 3.18)	2.7	16.4
**Anxiety**	640 (13.8)	45 (13.1)	0.95 (0.72 to 1.26)	1.08 (0.81 to 1.44)	-0.7	1.1
**Female**	531 (15.9)	34 (14.8)	0.94 (0.68 to 1.29)	1.04 (0.75 to 1.45)	-1.1	0.6
**Male**	106 (8.2)	10 (8.9)	1.08 (0.58 to 2.01)	1.11 (0.60 to 2.04)	0.7	1.0
**18-39 years**	237 (31.3)	13 (14.9)	0.48 (0.29 to 0.80)	0.52 (0.31 to 0.89)	-16.4	-13.4
**40-59 years**	286 (12.1)	27 (14.7)	1.21 (0.84 to 1.75)	1.62 (1.13 to 2.31)	2.6	9.7
**≥60 years**	117 (7.7)	5 (6.9)	0.90 (0.38 to 2.12)	1.56 (0.66 to 3.70)	-0.8	5.1
**PTSD**	747 (17.8)	59 (19.5)	1.09 (0.86 to 1.39)	1.26 (0.99 to 1.60)	1.7	5.4
**Female**	626 (21.1)	46 (22.7)	1.08 (0.83 to 1.40)	1.19 (0.91 to 1.56)	1.6	4.5
**Male**	119 (9.7)	12 (12.1)	1.25 (0.71 to 2.17)	1.52 (0.86 to 2.67)	2.4	6.5
**18-39 years**	168 (27.6)	11 (15.3)	0.55 (0.32 to 0.97)	0.68 (0.39 to 1.18)	-12.3	-9.6
**40-59 years**	376 (17.4)	31 (19.3)	1.11 (0.80 to 1.54)	1.31 (0.94 to 1.83)	1.9	6.9
**≥60 years**	203 (14.2)	17 (24.3)	1.70 (1.11 to 2.63)	2.22 (1.46 to 3.37)	10.1	24.6

[a] Adjusted for age (continuous variable), gender (male, female, or other), educational level (compulsory education, high school/trade school/other education, bachelor's degree/diploma certificate, or master's/Ph.D. degree), income (continuous variable), current smoking (yes or no), previous diagnosis of a psychiatric disorder (yes or no), number of previously diagnosed somatic diseases (0, 1, or ≥ 2), and timing of responding to the baseline questionnaire (April 24-30, May 1-7, May 8-23, or May 24-July 22). Adjustments are not made for the variable that is being stratified by.

Supplementary Table 6. Raw prevalence of flu-like symptoms during the preceding two months among individuals with and without a recent COVID-19 diagnosis.

	No. (%)		
	**Individuals not diagnosed with COVID-19 (n = 20488)**	**Individuals diagnosed with COVID-19 (n = 373)**	**P-value**
**No reported symptoms**			
Yes	3023 (14.8)	12 (3.2)	<.001
No	17465 (85.2)	361 (96.8)	
**Cough**			
Never	13181 (64.3)	122 (32.7)	<.001
1-2 days	3658 (17.9)	57 (15.3)	
3-6 days	1734 (8.5)	55 (14.7)	
1-2 weeks	754 (3.7)	54 (14.5)	
Over 2 weeks	1161 (5.7)	85 (22.8)	
**Fatigue and weakness**			
Never	7535 (36.8)	32 (8.6)	<.001
1-2 days	6044 (29.5)	39 (10.5)	
3-6 days	3350 (16.4)	54 (14.5)	
1-2 weeks	1276 (6.2)	59 (15.8)	
Over 2 weeks	2283 (11.1)	189 (50.7)	
**Fever**			
Never	17362 (84.7)	127 (34.0)	<.001
1-2 days	1907 (9.3)	58 (15.5)	
3-6 days	831 (4.1)	74 (19.8)	
1-2 weeks	227 (1.1)	53 (14.2)	
Over 2 weeks	161 (0.8)	61 (16.4)	
**Gastrointestinal symptoms**			
Never	12589 (61.4)	162 (43.4)	<.001
1-2 days	4565 (22.3)	69 (18.5)	
3-6 days	1941 (9.5)	61 (16.4)	
1-2 weeks	585 (2.9)	42 (11.3)	
Over 2 weeks	808 (3.9)	39 (10.5)	
**Headache**			
Never	8583 (41.9)	73 (19.6)	<.001
1-2 days	6754 (33.0)	57 (15.3)	
3-6 days	3381 (16.5)	95 (25.5)	
1-2 weeks	816 (4.0)	62 (16.6)	
Over 2 weeks	954 (4.7)	86 (23.1)	

For peer review only

	No. (%)		
	**Individuals not diagnosed with COVID-19 (n = 20488)**	**Individuals diagnosed with COVID-19 (n = 373)**	**P-value**
**Impaired sense of taste and/or smell**			
Never	19514 (95.2)	140 (37.5)	<.001
1-2 days	411 (2.0)	25 (6.7)	
3-6 days	220 (1.1)	44 (11.8)	
1-2 weeks	111 (0.5)	55 (14.7)	
Over 2 weeks	232 (1.1)	109 (29.2)	
**Myalgia**			
Never	12253 (59.8)	105 (28.2)	<.001
1-2 days	3512 (17.1)	54 (14.5)	
3-6 days	2072 (10.1)	72 (19.3)	
1-2 weeks	708 (3.5)	81 (21.7)	
Over 2 weeks	1943 (9.5)	61 (16.4)	
**Shortness of breath**			
Never	17111 (83.5)	169 (45.3)	<.001
1-2 days	1809 (8.8)	42 (11.3)	
3-6 days	775 (3.8)	55 (14.7)	
1-2 weeks	312 (1.5)	41 (11.0)	
Over 2 weeks	481 (2.3)	66 (17.7)	
**Sore throat**			
Never	12987 (63.4)	153 (41.0)	<.001
1-2 days	4323 (21.1)	62 (16.6)	
3-6 days	1901 (9.3)	63 (16.9)	
1-2 weeks	632 (3.1)	39 (10.5)	
Over 2 weeks	645 (3.1)	56 (15.0)	

For peer review only

Supplementary Table 7. Prevalence, crude and adjusted relative risks with 95% confidence intervals of flu-like symptoms lasting for 1 week or more, during the preceding two months, among individuals who tested negative for SARS-CoV-2 and those who were never tested.

Symptoms	No. (%) Individuals not tested for SARS-CoV-2 (n = 15442)	No. (%) Individuals tested negative for SARS-CoV-2 (n = 5046)	cRR (95% CI)	aRR (95% CI)[a]
Cough	1249 (8.1)	666 (13.2)	1.60 (1.46 to 1.76)	1.59 (1.45 to 1.75)
Fatigue and weakness	2420 (15.7)	1139 (22.3)	1.45 (1.36 to 1.55)	1.37 (1.29 to 1.46)
Fever	176 (1.1)	212 (4.2)	3.71 (3.02 to 4.56)	3.49 (2.84 to 4.29)
Gastrointestinal symptoms	946 (6.1)	447 (8.9)	1.48 (1.33 to 1.66)	1.37 (1.23 to 1.53)
Headache	1134 (7.3)	636 (12.6)	1.74 (1.58 to 1.91)	1.60 (1.46 to 1.75)
Impaired sense of taste or smell	229 (1.5)	114 (2.3)	1.53 (1.21 to 1.93)	1.64 (1.30 to 2.07)
Myalgia	1881 (12.2)	770 (15.3)	1.26 (1.16 to 1.36)	1.29 (1.19 to 1.39)
Shortness of breath	503 (3.3)	290 (5.7)	1.88 (1.62 to 2.17)	1.84 (1.59 to 2.13)
Sore throat	778 (5.0)	499 (9.9)	1.98 (1.77 to 2.21)	1.91 (1.71 to 2.14)

[a] Adjusted for age (continuous variable), gender (male, female, or other), educational level (compulsory education, high school/trade school/other education, bachelor's degree/diploma certificate, or master's/Ph.D. degree), income (continuous variable), current smoking (yes or no), previous diagnosis of a psychiatric disorder (yes or no), number of previously diagnosed somatic diseases (0, 1, or ≥ 2), and timing of responding to the baseline questionnaire (April 24-30, May 1-7, May 8-23, or May 24-July 22).

For peer review only

Supplementary Table 8. Prevalence, crude and adjusted relative risks with 95% confidence intervals of flu-like symptoms lasting for 1 week or more, during the preceding two months, among individuals with and without a confirmed diagnosis of COVID-19.

	No. (%)			
**Symptoms**	**Individuals not diagnosed with COVID-19 (n = 20488)**	**Individuals diagnosed with COVID-19 (n = 373)**	**cRR (95% CI)**	**aRR (95% CI)[a]**
Cough	1915 (9.4)	139 (37.3)	4.15 (3.60 to 4.76)	5.02 (4.35 to 5.81)
Fatigue and weakness	3559 (17.4)	248 (66.5)	3.93 (3.63 to 4.26)	4.16 (3.77 to 4.60)
Fever	388 (1.9)	114 (30.6)	16.77 (13.93 to 20.19)	19.10 (15.49 to 23.48)
Gastrointestinal symptoms	1393 (6.8)	81 (21.7)	3.29 (2.69 to 4.06)	3.49 (2.82 to 4.32)
Headache	1770 (8.6)	148 (39.7)	4.67 (4.10 to 5.37)	4.64 (3.99 to 5.39)
Impaired sense of taste or smell	343 (1.7)	164 (44.0)	27.04 (23.10 to 31.82)	32.52 (27.22 to 38.86)
Myalgia	2651 (12.9)	142 (38.1)	3.03 (2.65 to 3.49)	3.66 (3.16 to 4.23)
Shortness of breath	793 (3.9)	107 (28.7)	7.53 (6.05 to 9.03)	9.26 (7.61 to 11.25)
Sore throat	1277 (6.2)	95 (25.5)	4.17 (3.46 to 5.00)	4.58 (3.78 to 5.54)

[a] Adjusted for age (continuous variable), gender (male, female, or other), educational level (compulsory education, high school/trade school/other education, bachelor's degree/diploma certificate, or master's/Ph.D. degree), income (continuous variable), current smoking (yes or no), previous diagnosis of a psychiatric disorder (yes or no), number of previously diagnosed somatic diseases (0, 1, or ≥ 2), and timing of responding to the baseline questionnaire (April 24-30, May 1-7, May 8-23, or May 24-July 22).

For peer review only

Supplementary Table 9. Adjusted mean symptom scores of depression, anxiety and PTSD by flu-like symptom burden in tertiles among individuals with a recent COVID-19 diagnosis, those who tested negative and those never tested for SARS-CoV-2.

	Multivariable adjusted mean value by tertile of flu-like symptoms[a]				
	**Lowest tertile**	**Mid tertile**	**Highest tertile**	**Beta (95% CI)**	**P-value**
**Depression**					
Patients with COVID-19	5.49	7.30	10.89	0.24 (0.17 to 0.30)	<.001
Tested negative for SARS-CoV-2	6.33	9.48	10.75	0.29 (0.25 to 0.33)	<.001
Not tested for SARS-CoV-2	6.04	9.79	11.34	0.34 (0.33 to 0.36)	<.001
**Anxiety**					
Patients with COVID-19	4.31	5.29	7.02	0.12 (0.06 to 0.17)	<.001
Tested negative for SARS-CoV-2	5.06	7.16	8.02	0.19 (0.16 to 0.23)	.03
Not tested for SARS-CoV-2	4.81	7.04	8.17	0.22 (0.20 to 0.23)	<.001
**PTSD**					
Patients with COVID-19	1.85	2.26	2.50	0.03 (0.01 to 0.05)	<.001
Tested negative for SARS-CoV-2	1.88	2.43	2.21	0.05 (0.04 to 0.06)	.02
Not tested for SARS-CoV-2	1.82	2.31	2.59	0.06 (0.06 to 0.07)	<.001

[a] Adjusted for age (continuous variable), gender (male, female, or other), educational level (compulsory education, high school/trade school/other education, bachelor's degree/diploma certificate, or master's/Ph.D. degree), income (continuous variable), current smoking (yes or no), previous diagnosis of a psychiatric disorder (yes or no), number of previously diagnosed somatic diseases (0, 1, or ≥ 2), and timing of responding to the baseline questionnaire (April 24-30, May 1-7, May 8-23, or May 24-July 22).

Reporting checklist for cross sectional study.

Based on the STROBE cross sectional guidelines.

Instructions to authors

Complete this checklist by entering the page numbers from your manuscript where readers will find each of the items listed below.

Your article may not currently address all the items on the checklist. Please modify your text to include the missing information. If you are certain that an item does not apply, please write "n/a" and provide a short explanation.

Upload your completed checklist as an extra file when you submit to a journal.

In your methods section, say that you used the STROBE cross sectionalreporting guidelines, and cite them as:

von Elm E, Altman DG, Egger M, Pocock SJ, Gotzsche PC, Vandenbroucke JP. The Strengthening the Reporting of Observational Studies in Epidemiology (STROBE) Statement: guidelines for reporting observational studies.

		Reporting Item	Page Number
**Title and abstract**			
Title	#1a	Indicate the study's design with a commonly used term in the title or the abstract	1
Abstract	#1b	Provide in the abstract an informative and balanced summary of what was done and what was found	3-4
**Introduction**			
Background / rationale	#2	Explain the scientific background and rationale for the investigation being reported	6
Objectives	#3	State specific objectives, including any prespecified hypotheses	7
**Methods**			
Study design	#4	Present key elements of study design early in the paper	7-8

For peer review only

Setting	#5	Describe the setting, locations, and relevant dates, including periods of recruitment, exposure, follow-up, and data collection	7-8
Eligibility criteria	#6a	Give the eligibility criteria, and the sources and methods of selection of participants.	7-8
	#7	Clearly define all outcomes, exposures, predictors, potential confounders, and effect modifiers. Give diagnostic criteria, if applicable	8-10
Data sources / measurement	#8	For each variable of interest give sources of data and details of methods of assessment (measurement). Describe comparability of assessment methods if there is more than one group. Give information separately for for exposed and unexposed groups if applicable.	8-10
Bias	#9	Describe any efforts to address potential sources of bias	19-20
Study size	#10	Explain how the study size was arrived at	7-8
Quantitative variables	#11	Explain how quantitative variables were handled in the analyses. If applicable, describe which groupings were chosen, and why	8-10
Statistical methods	#12a	Describe all statistical methods, including those used to control for confounding	10-11
Statistical methods	#12b	Describe any methods used to examine subgroups and interactions	10-11
Statistical methods	#12c	Explain how missing data were addressed	11
Statistical methods	#12d	If applicable, describe analytical methods taking account of sampling strategy	n/a
Statistical methods	#12e	Describe any sensitivity analyses	10-11
**Results**			
Participants	#13a	Report numbers of individuals at each stage of study—eg numbers potentially eligible, examined for eligibility, confirmed eligible, included in the study, completing	12-14

For peer review only

		follow-up, and analysed. Give information separately for for exposed and unexposed groups if applicable.	
Participants	#13b	Give reasons for non-participation at each stage	7-8 (addressed in the methods chapter)
Participants	#13c	Consider use of a flow diagram	Supplementary material: page 1, Supplementary Figure 1
Descriptive data	#14a	Give characteristics of study participants (eg demographic, clinical, social) and information on exposures and potential confounders. Give information separately for exposed and unexposed groups if applicable.	12-14
Descriptive data	#14b	Indicate number of participants with missing data for each variable of interest	7-8 (addressed in the methods chapter)
Outcome data	#15	Report numbers of outcome events or summary measures. Give information separately for exposed and unexposed groups if applicable.	14-17
Main results	#16a	Give unadjusted estimates and, if applicable, confounder-adjusted estimates and their precision (eg, 95% confidence interval). Make clear which confounders were adjusted for and why they were included	14-16
Main results	#16b	Report category boundaries when continuous variables were categorized	9-10 (addressed in the methods chapter)
Main results	#16c	If relevant, consider translating estimates of relative risk into absolute risk for a meaningful time period	14-16
Other analyses	#17	Report other analyses done—e.g., analyses of subgroups and interactions, and sensitivity analyses	14-15
**Discussion**			
Key results	#18	Summarise key results with reference to study objectives	18
Limitations	#19	Discuss limitations of the study, taking into account sources of potential bias or imprecision. Discuss both direction and magnitude of any potential bias.	19-20

For peer review only

Interpretation	#20	Give a cautious overall interpretation considering objectives, limitations, multiplicity of analyses, results from similar studies, and other relevant evidence.	18-20
Generalisability	#21	Discuss the generalisability (external validity) of the study results	20
**Other Information**			
Funding	#22	Give the source of funding and the role of the funders for the present study and, if applicable, for the original study on which the present article is based	21

Notes:

- 13b: 7-8 (addressed in the methods chapter)
- 13c: Supplementary material: page 1, Supplementary Figure 1
- 14b: 7-8 (addressed in the methods chapter)
- 16b: 9-10 (addressed in the methods chapter)

The STROBE checklist is distributed under the terms of the Creative Commons Attribution License CC-BY. This checklist was completed on 15. January 2021 using https://www.goodreports.org/, a tool made by the EQUATOR Network in collaboration with Penelope.ai

For peer review only
